# Design, Synthesis and Biological Investigation of 2-Anilino Triazolopyrimidines as Tubulin Polymerization Inhibitors with Anticancer Activities

**DOI:** 10.3390/ph15081031

**Published:** 2022-08-21

**Authors:** Romeo Romagnoli, Paola Oliva, Filippo Prencipe, Stefano Manfredini, Federica Budassi, Andrea Brancale, Salvatore Ferla, Ernest Hamel, Diana Corallo, Sanja Aveic, Lorenzo Manfreda, Elena Mariotto, Roberta Bortolozzi, Giampietro Viola

**Affiliations:** 1 Department of Chemical, Pharmaceutical and Agricultural Sciences, University of Ferrara, 44121 Ferrara, Italy; 2 Department of Chemical and Pharmaceutical Sciences, University of Trieste, 34127 Trieste, Italy; 3 Department of Life Sciences and Biotechnology, University of Ferrara, 44121 Ferrara, Italy; 4 Medicinal Chemistry Department, Integrated Drug Discovery, Aptuit-An Evotec Company, 37135 Verona, Italy; 5 School of Pharmacy and Pharmaceutical Sciences, Cardiff University, King Edward VII Avenue, Cardiff CF10 3NB, UK; 6 Faculty of Medicine, Health and Life Science, Swansea University Medical School, Grove Building, Swansea University, Swansea SA2 8PP, UK; 7 Molecular Pharmacology Branch, Developmental Therapeutics Program, Division of Cancer Treatment and Diagnosis, Frederick National Laboratory for Cancer Research, National Cancer Institute, National Institutes of Health, Frederick, MD 21702, USA; 8 Laboratory of Target Discovery and Biology of Neuroblastoma, Istituto di Ricerca Pediatrica (IRP), Fondazione Città della Speranza, 35128 Padova, Italy; 9 Department of Woman’s and Child’s Health, Hemato-Oncology Lab, University of Padova, 35131 Padova, Italy; 10 Laboratory of Experimental Pharmacology, Istituto di Ricerca Pediatrica (IRP), Fondazione Città della Speranza, 35128 Padova, Italy

**Keywords:** colchicine binding site, antitumor activity, [1,2,4]triazolo[1,5-*a*]pyrimidine, apoptosis, microtubule-targeting agents

## Abstract

A further investigation aiming to generate new potential antitumor agents led us to synthesize a new series of twenty-two compounds characterized by the presence of the 7-(3′,4′,5′-trimethoxyphenyl)-[1,2,4]triazolo[1,5-*a*]pyrimidine pharmacophore modified at its 2-position. Among the synthesized compounds, three were significantly more active than the others. These bore the substituents *p*-toluidino (**3d**), *p*-ethylanilino (**3h**) and 3′,4′-dimethylanilino (**3f**), and these compounds had IC_50_ values of 30–43, 160–240 and 67–160 nM, respectively, on HeLa, A549 and HT-29 cancer cells. The *p*-toluidino derivative **3d** was the most potent inhibitor of tubulin polymerization (IC_50_: 0.45 µM) and strongly inhibited the binding of colchicine to tubulin (72% inhibition), with antiproliferative activity superior to CA-4 against A549 and HeLa cancer cell lines. In vitro investigation showed that compound **3d** was able to block treated cells in the G2/M phase of the cell cycle and to induce apoptosis following the intrinsic pathway, further confirmed by mitochondrial depolarization and caspase-9 activation. In vivo experiments conducted on the zebrafish model showed good activity of **3d** in reducing the mass of a HeLa cell xenograft. These effects occurred at nontoxic concentrations to the animal, indicating that **3d** merits further developmental studies.

## 1. Introduction

Eukaryotic cells possess a complex cytoskeletal system. One of its major components is the microtubule network, composed of polymeric protein structures formed by the assembly of αβ-tubulin heterodimers, which are in a dynamic equilibrium with the microtubules. These are critical for several fundamental cellular functions, including cell division, where microtubules form the mitotic spindle required for proper chromosomal separation [1,2,3,4]. Since microtubules are critical in the proliferation of cancer cells, the design of new and improved molecules that exert their effects by interfering with microtubule dynamics continues to receive substantial attention [5,6,7]. 

Among the seven distinct classes of tubulin-binding agents that have been identified on the basis of their different mechanisms of action and binding sites on tubulin, only three groups of antimitotic agents are used clinically for cancer treatment [8,9]. First are the vinca alkaloids, typified by vinblastine, vincristine, vinorelbine and vinflunine, that destabilize microtubules by inhibiting polymerization of tubulin into microtubules [10,11,12]. Second is the halichondrin B analogue eribulin, which also inhibits tubulin polymerization. Third, are compounds that bind in the taxoid site and stabilize microtubules. This group includes paclitaxel, the structurally related compounds docetaxel and cabazitaxel and the epothilone analogue ixabepilone [13,14,15]. These drugs are in clinical use to treat both adult and pediatric cancers [16,17,18,19]. Taxoid site compounds, eribulin and vinca alkaloids all interact with β-tubulin, but at different binding sites [12,14]. Nevertheless, the clinical success of these three classes of compounds has stimulated intensive research aimed at discovering additional microtubule-targeting drugs with clinical potential [20,21,22,23]. 

**Figure 1:**
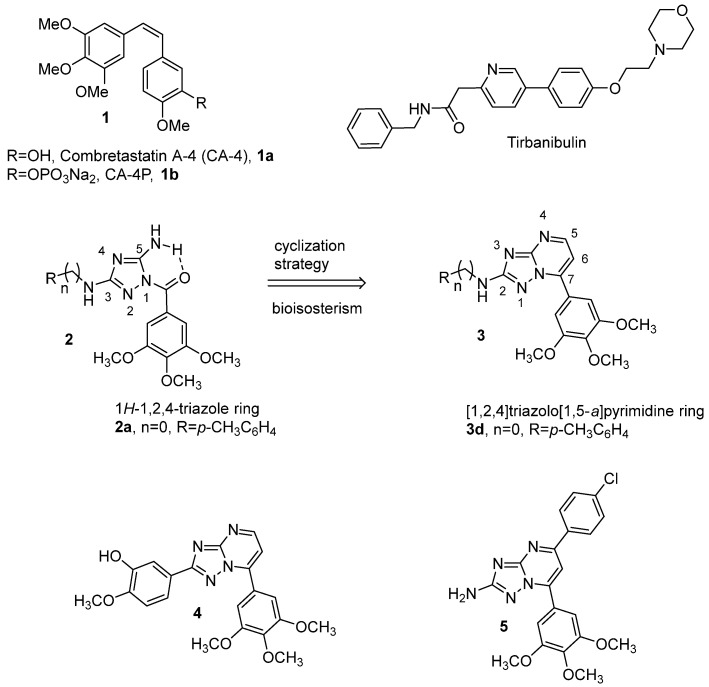
Structures of CA-4 (**1a**), CA-4P (**1b**), tirbanibulin, general structure (**2**) of 1-(3′,4′,5′-trimethoxybenzoyl)-5-amino-1,2,4-triazoles and [1,2,4]triazolo[1,5-*a*]pyrimidines reported in this manuscript (**3**) and reported in the literature (**4** and **5**).

The colchicine site agents that inhibit tubulin assembly belong to another important class of tubulin interactive compounds, due to the discovery of numerous natural products and synthetic compounds with wide structural heterogeneity that target this site [24,25,26,27]. The colchicine site is located on β-tubulin at its interface with α-tubulin and is distinct from the vinca and eribulin sites [25,26]. One of the most notable of these compounds, both for its potency and structural simplicity, is the naturally occurring polymethoxylated *cis*-stilbene derivative combretastatin A-4 (CA-4, **1a**, Figure 1) [28,29]. Moreover, several studies have documented that this compound also acts as a powerful vascular disrupting agent [30], particularly for the neovasculature of tumors. To improve its water solubility, the corresponding phosphate derivative of combretastatin A-4 (CA-4P, also known as fosbretabulin disodium, **1b**) was synthesized as a water-soluble pro-drug of CA-4 [31]. The European Medicines Agency (EMA) approved combretastatin CA-4P as an orphan drug for the treatment of anaplastic thyroid cancer and ovarian cancer, and CA-4P is in phase III clinical trials in combination with bevacizumab for recurrent ovarian cancer or with everolimus in neuroendocrine tumors [32,33].

While the vinca alkaloids and the taxoids have achieved notable success in the treatment of human cancers, no colchicine site inhibitor has yet been approved for cancer chemotherapy in human patients [34,35,36]. KXO1 (tirbanibulin or KX2–391), a dual Src and tubulin polymerization inhibitor acting at the colchicine site of β-tubulin [37], was approved by the FDA in December 2020 for the topical treatment of actinic or solar keratosis, the most common chronic and precancerous skin disease that occurs on skin damaged by long-term exposure to ultraviolet radiation [38].

Emerging multidrug resistance (MDR) due to the overexpression of membrane-bound drug efflux proteins, such as ATP binding cassette (ABC) transporters, along with aberrant expression of the βIII-tubulin isoform, has limited the use of taxoids and vinca alkaloids in clinical practice [39,40,41,42]. Resistance to different types of microtubule-targeting agents was recently suggested to be related to their binding sites and that βIII-tubulin-mediated drug resistance might be circumvented by colchicine site inhibitors [43,44,45,46,47]. These observations emphasize the need to discover novel scaffolds active at the colchicine site and amenable to rapid derivatization because such compounds could be readily prepared by rapid and concise synthetic procedures [48,49]. In addition, several colchicine site binders have been shown to be effective against p53-mutant cell lines and to circumvent P-glycoprotein-mediated multidrug resistance. This lack of susceptibility to MDR pumps further emphasizes the importance of novel inhibitors of tubulin assembly that have activity against drug-resistant tumors [50,51,52].

In a previous article, we designed and synthesized a new class of antimitotic agents with general structure **2** based on the 3-arylamino-5-amino-1,2,4 triazole moiety [53]. The structure–activity relationship study was carried out on the phenyl ring of the anilino moiety present on the C-3 position of a triazole scaffold. Compound **2a**, with a para-methyl substitution on the phenyl ring, exhibited potent antiproliferative effects in the low nanomolar range on several cancer cell lines. This compound was almost twice as potent as CA-4 against inhibition of tubulin polymerization, displaying an IC_50_ value of 0.75 μM. The X-ray crystal structure of compound **2a** showed the formation of an intramolecular hydrogen bond between the carbonyl oxygen of the 3′,4′,5′-trimethoxybenzoyl function and the hydrogen of the amino moiety at the 1- and 5-position, respectively, of the 1,2,4-triazole ring. In the present study, a cyclization approach was adopted to form an additional six-membered fused pyrimidine ring that incorporated these elements, the carbonyl moiety and the amino group, to obtain the bicyclic [1,2,4]triazolo[1,5-*a*]pyrimidine framework **3**. This scaffold hopping approach strategy [54,55] also revealed a potential bioisosteric relationship between the 1-(3′,4′,5′-trimethoxybenzoyl)-5-amino-1,2,4-triazole structure and the 7-(3′,4′,5′-trimethoxyphenyl)[1,2,4]triazolo[1,5-*a*]pyrimidine system. In addition, several studies have shown that the carbonyl moiety is a metabolically labile site and that carbonyl reduction to a secondary alcohol is one of the major metabolic processes used by liver microsomes [56]. A promising approach to overcome this metabolic instability is by incorporating the carbonyl moiety into a five- or six-membered heterocyclic ring [57,58,59,60].

The [1,2,4]triazolo [1,5-*a*]pyrimidine scaffold incorporating the 1,2,4-triazole nucleus (Figure 1) was previously identified by us and others as a promising nucleus for the design of new microtubule-destabilizing agents [61,62,63,64,65]. Two different series of compounds that retain the 3,4,5-trimethoxyphenyl ring at the 7-position of the triazolopyrimidine core variably functionalized at its 2-position were evaluated for their antitumor activity [62,63,64]. 

Yang et al. reported a novel series of 2,7-diaryl-[1,2,4]triazolo[1,5-*a*]pyrimidine derivatives substituted at the 2- and 7-positions as tubulin polymerization inhibitors [62,63]. By inversion of the C-2/C-7 substituents on the triazolopyrimidine ring, compound **4**, characterized by the presence of 4′-methoxy-3′-hydroxyphenyl and 3′,4′,5′-trimethoxyphenyl moieties at the 2- and 7-positions, respectively, of the triazolopyrimidine scaffold display potent and selective antiproliferative activity (IC_50_: 60 nM) against HeLa cells [63]. The same compound showed reduced inhibitory activity against a panel of four cancer cell lines with IC_50_ values of 3–18 μM. Mechanism studies indicate that **4** exerts antiproliferative effects by inhibition of tubulin assembly, with 3-fold greater potency than CA-4, and these data, together with data obtained with compound **5** recently identified by Mohamed et al. [64], confirm that the 3′,4′,5′-trimethoxyphenyl ring located at the 7-position of the triazolopyrimidine system contributes to maximal activity. This latter derivative, as an analogue of **4** where the aryl ring was moved from the 2- to the 5-position of the 7-(3′,4′,5′-trimethoxyphenyl) triazolopyrimidine scaffold and replaced by an amino group, exhibits significant antiproliferative activity (IC_50_: 0.53 μM) against the HCT-116 cancer cell line, with four-fold less activity than CA-4 as a tubulin polymerization inhibitor (IC_50_: 3.84 and 1.1 μM, respectively) [64].

**Figure 2:**
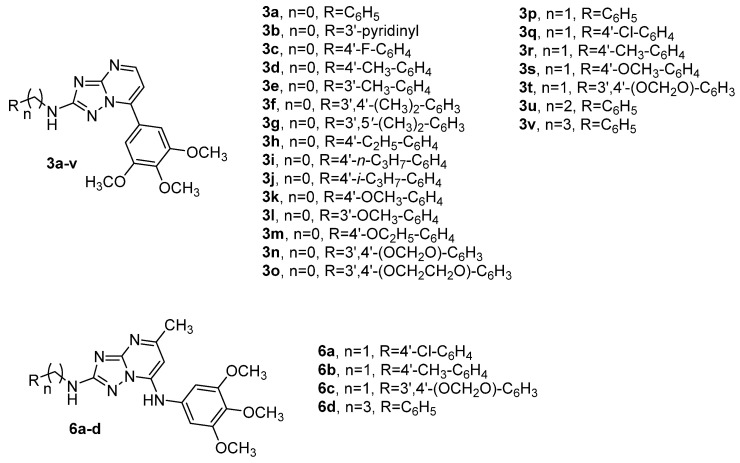
Chemical structures of newly synthesized compounds **3a–v** based on the 7-(3′,4′,5′-trimethoxyphenyl)[1,2,4]triazolo[1,5-*a*]pyrimidine scaffold and general molecular formula **6a–d** of triazolopyrimidines published by us (ref. [65]).

Thus, once the 7-(3′,4′,5′-trimethoxyphenyl)-[1,2,4]triazolo[1,5-*a*]pyrimidine motif was identified as the minimum structural requirement for antimitotic activity, in the new class of designed **compounds 3a–v** reported in this article, modifications were focused on varying the substituent at the 2-position of the triazolopyrimidine ring to maximize activity against cancer cell lines (Figure 2). For the anilino derivative **3a**, we evaluated the replacement of phenyl with a bioisosteric pyrimidin-2-yl ring, to yield **3b**. For compounds **3c–o**, characterized by the presence of aromatic amines at the 2-position of the triazolopyrimidine scaffold, modifications were focused on varying the substituents on the phenyl ring of the anilino moiety, by introduction electron-withdrawing (F) or electron-releasing (alkyl or alkoxy) groups. 

We also investigated a further structural modification by the synthesis of compounds **3p–v**, characterized by the presence of a methylene (-CH_2_)-), ethylene (-CH_2_CH_2_-) or propylene (-(CH_2_)_3_-) spacer between the nitrogen at the 2-position of the triazolopyrimidine scaffold and the phenyl ring, to furnish compounds **3p–t**, **3u** and **3v**, respectively. By the preparation of this latter small series, we evaluated if an increased flexibility of the substituent at the 2-position of the triazolopyrimidine scaffold was correlated with further enhancement of antiproliferative activity. The design of the linker chain in derivatives **3p–t** was based on preserving flexibility similar to that of a series of compounds with general structure **6** previously published by us [65], characterized by a common 7-(3′,4′,5′-trimethoxyanilino)-[1,2,4]triazolo[1,5-*a*]pyrimidine scaffold and modified at its 2-position. Three benzylamino derivatives, **6a** (4′-Cl), **6b** (4′-Me) and **6c** (3′,4′-methylendioxy), along with the 3′-phenylpropylamino derivative **6d**, were the most active compounds identified in this series. 

## 2. Results and Discussion

### 2.1. Chemistry

**Scheme 1:**
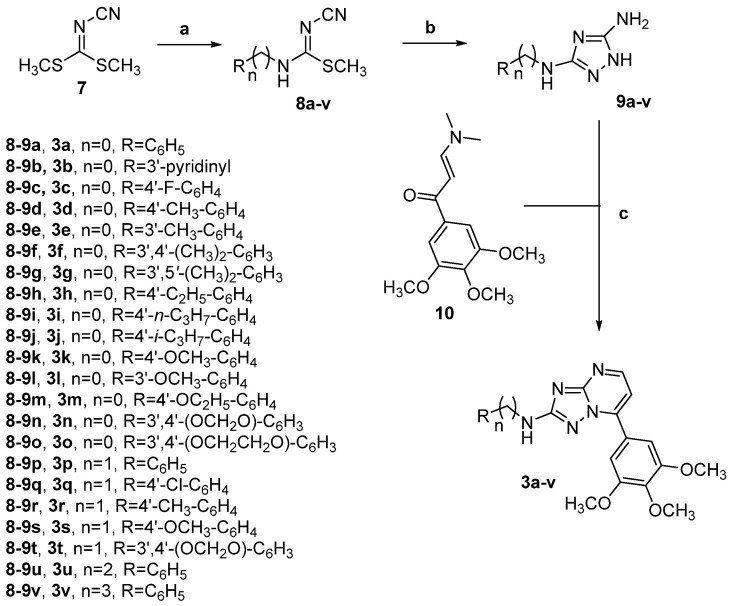
Reagents. **a**: Appropriate ArNH_2_, *i*-PrOH, reflux, 18 h for the preparation of compounds **8a–o** or appropriate ArCH_2_NH_2_/C_6_H_5_(CH_2_)_2_NH_2_/C_6_H_5_(CH_2_)_3_NH_2_, *i*-PrOH, room temperature, 18 h for the synthesis of derivatives **8p–v**, 75–92%; **b**: NH_2_NH_2_.H_2_O, MeOH, reflux, 18 h, 68–93%; **c**: AcOH, 80 °C, 2 h, 47–66%.

The 2-substituted-7-(3′,4′,5′-trimethoxyphenyl)[1,2,4]triazolo[1,5-*a*]pyrimidines **3a–v** were synthesized using a three-step synthetic procedure described in Scheme 1. The condensation of dimethyl cyanodithioimidocarbonate **7** with the appropriate amine (aniline or substituted aniline, benzylamine or substituted benzylamine, 2′-phenylethylamine or 3′-phenylpropylamine) resulted in the formation of imidates **8a–v**, which were cyclized into the corresponding 3-substituted 5-amino-1*H*-[1,2,4]-triazole derivatives **9a–v** in the presence of hydrazine hydrate in refluxing methanol. 

Finally, the cyclization reaction of compounds **9a–v** with enaminone **10** in glacial acetic acid at 80 °C for 2 h resulted in 2-substituted-7-(3′,4′,5′-trimethoxyphenyl)-[1,2,4]triazolo[1,5-*a*]pyrimidine derivatives **3a–v** in good yield. As reported in the literature, enaminone **10** was obtained by the condensation of 3′,4′,5′-trimethoxyacetophenone with dimethylformamide dimethyl acetal (DMF-DMA) as the solvent/reagent at 120 °C for 6 h [66]. The ^1^H NMR and ^13^C NMR spectra of derivatives **3a–v** are presented in the Supplementary Materials.

### 2.2. Biological Activity and Molecular Docking Studies

#### 2.2.1. In Vitro Antiproliferative Activities

The series of 2-substituted 7-(3′,4′,5′-trimethoxyphenyl)[1,2,4]triazolo[1,5-*a*]pyrimidine derivatives **3a–v** was screened for antiproliferative activity against a panel of four different human cancer cell lines and compared with the reference compound CA-4 (**1a**) (Table 1). The cell lines used were a breast cancer cell (MDA-MB-231), a cervix carcinoma (HeLa), a non-small cell lung carcinoma (A549) and a colon adenocarcinoma (HT-29). CA-4 had single-digit nanomolar activity (IC_50_: 4–5 nM) against MDA-MB-231 and HeLa cancer cell lines, while A549 and HT-29 cells were more resistant to CA-4, with IC_50_ values of 180 and 3100 nM, respectively. Eight of twenty-two compounds were found to be more active than CA-4 against HT-29 cells, while only derivative **3d** was more potent (4-fold) than CA-4 against A549 cells. The compounds were generally less active against the MDA-MB-231 cells.

From a structure–activity point of view, the presence of a 3,4,5-trimethoxyphenyl ring at position 7 of the triazolopyrimidine scaffold combined with the *p*-toluidino moiety at position 2 were important structural features for this class of compounds. The *p*-toluidino derivative **3d** was the most active compound of the series, showing similar activity toward the HeLa, A549 and HT29 cancer cell lines (IC_50_: 38, 43 and 30 nM, respectively), with ten-fold reduced antiproliferative activity (IC_50_: 0.43 μM) toward MDA-MB-231 cells. The activity of **3d** was 4- and 100-fold greater than that of the reference compound CA-4 against A549 and HT-29 cells, respectively, but was one and two orders of magnitude less potent than CA-4 against MDA-MB-231 and HeLa cells, respectively. Compound **3d** was 4–30-fold more potent as an antiproliferative agent than the corresponding 5-methyl-2-(*p*-toluidino)-7-(3′,4′,5′-trimethoxyanilino)-[1,2,4]triazolo[1,5-*a*]pyrimidine counterpart previously published by us (IC_50_ 0.43–0.030 μM and 0.52–1.55 μM, respectively) [65]. 

While the aniline derivative **3a** showed antiproliferative activity ranging from 0.80 to 2.27 μM against the four cancer cell lines, increasing the length of the alkyl spacer between the phenyl ring and the nitrogen at the 2-position of the triazolopyrimidine scaffold from one (**3p**) to two (**3u**) to three (**3v**) methylene units caused a profound loss of activity compared with **3a**, with IC_50_ values generally > 10 μM. 

Replacement of the unsubstituted phenyl ring of compound **3a** with the bioisosteric pyridin-3-yl moiety (compound **3b**) also dramatically reduced antiproliferative activity against three of the four cancer cell lines (IC_50_ > 10 μM), with a 3-fold reduction in potency against HT-29 cells (IC_50_: 1.02 and 3.42 μM, respectively).

The substitution pattern on the phenyl ring of the arylamino moiety at the 2-position of the [1,2,4]triazolo[1,5-*a*]pyrimidine nucleus ring played an important role in antiproliferative activity. While the introduction of the electron-withdrawing fluorine atom at the para-position of the phenyl group, to furnish **3c**, was detrimental for activity relative to the unsubstituted phenyl derivative **3a**, the weak electron-releasing *p*-methyl group, to furnish compound **3d**, enhanced biological activity and resulted in the most active compound of the whole series.

The position of the methyl on the phenyl ring was critical for the antiproliferative activity of **3d**. Significant loss in activity (10–46-fold) occurred when the methyl group was moved from the para- to the meta-position, to obtain the isomeric derivative **3e**. The reduction in potency was most dramatic against MDA-MB-231 cells (IC_50_ > 10 μM).

Relative to the activity of the *p*-toluidino derivative **3d**, the insertion of an additional methyl group at the meta-position of the *p*-tolyl moiety, to furnish the *m*,*p*-xylyl derivative **3f**, reduced antiproliferative activity 2–5-fold on three cancer cells, and the reduction in potency was most pronounced against MDA-MB-231 cells (IC_50_ >10 μM), while the isomeric *m*,*m*′-xylyl analogue **3g** was completely inactive (IC_50_ > 10 μM). Lengthening the alkyl chain from methyl (**3d**) to ethyl (**3h**) resulted in a 5–7-fold reduction in antiproliferative activity, most pronounced with the MDA-MB-231 cells (IC_50_ > 10 μM). 

The data showed that the homologation of the alkyl chain from ethyl to *n*-propyl or isopropyl (compounds **3i** and **3j**, respectively) at the para-position of the phenyl ring was not tolerated and provided a strong reduction in activity against all four cancer cell lines.

For the *p*-toluidine derivative **3d**, replacement of the methyl moiety by the stronger electron-releasing methoxy group, to furnish the *p*-anisidino analogue **3k**, was detrimental for antiproliferative activity, which was reduced 12–124-fold as compared to **3d**. In contrast to the two isomeric toluidine derivatives **3d** and **3e**, comparing the biological activities of the para- and meta-anisidino derivatives **3k** and **3l**, respectively, the highest antiproliferative activity was observed with the methoxy moiety at the meta-position of the phenyl ring. As observed for the *p*-toluidino derivative **3d**, with the *p*-anisidino **3k**, homologation of the alkyl chain from methoxy to ethoxy (compound **3m**) was detrimental for antiproliferative activity against all four cancer cell lines (IC_50_ > 10 μM).

Replacement of the *p*-methoxy with a 3′,4′-methylendioxy moiety (compounds **3k** and **3n**, respectively) produced a 1.5- to 4-fold increase in antiproliferative activity against three of the four cancer cell lines, while **3k** and **3n** were equipotent against MDA-MB-231 cells. For derivative **3n**, the introduction of an additional methylene unit between the two oxygens, to yield the 3′,4′-ethylenedioxy homologue **3o**, had contrasting effects, producing a reduction in potency against MDA-MB-231 and A549 cells, while **3o** was 2- and 3-fold more active than **3n** against HT-29 and HeLa cancer cells, respectively.

With the anilino derivatives **3a** (phenyl), **3d** (*p*-tolyl), **3k** (*p*-methoxyphenyl) and **3n** (3′,4′-methylendioxyphenyl), the corresponding benzylamino analogues **3p**, **3r**, **3s** and **3t**, respectively, showed a dramatic drop in potency (IC_50_ > 10 µM). 

In conclusion, SAR studies have underlined that the presence of small substituents such as methyl or ethyl at the para-position as well as two methyl groups at the meta- and para-positions (compounds **3d**, **3h** and **3f**, respectively) on the phenyl ring of the aniline moiety are critical for optimal antiproliferative activity. Electron-releasing substituents at the para-position of the phenyl ring showed antiproliferative activity in the order of Me > Et > OMe ≫ OEt = *n*-C_3_H_7_ = *i*-C_3_H_7_.

#### 2.2.2. In Vitro Inhibition of Tubulin Polymerization and Colchicine Binding

To investigate whether the antiproliferative activities of tested compounds derived from an interaction with microtubules, the most active molecules **3d**, **3f**, **3h** and **3l** were selected to determine their inhibition of tubulin polymerization and for effects on the binding of [^3^H]colchicine to tubulin (Table 2). For comparison, CA-4 was examined in contemporaneous experiments.

In the assembly assay, compound **3d** was found to be the most active (IC_50_: 0.45 μM), and it was almost twice as potent as CA-4 (IC_50_: 0.75 μM), while **3f** was equipotent to CA-4. When comparing the inhibition of tubulin polymerization versus the growth inhibitory effect, we found that compound **3d,** although it was 2-fold more active than CA-4 as an inhibitor of tubulin assembly, was 10- and 100-fold less potent than CA-4 as an antiproliferative agent against HeLa and MDA-MB-231 cells, respectively. The reduced potency of **3d** on these two cancer cell lines can possibly be rationalized by any mechanism limiting the accessibility of this molecule to the cellular tubulin. Compounds **3h** and **3l** showed weak antitubulin polymerization activity (IC_50_: 1.9 and 2.2 μM, respectively), which is consistent with their low antiproliferative activities.

In competition experiments, in reaction mixtures containing 0.5 μM tubulin and 5.0 μM [^3^H]colchicine, compound **3d** strongly inhibited the binding of [^3^H]colchicine to tubulin, with 72% inhibition occurring when **3d** and the radiolabeled drug were both at 5.0 μM in the reaction mixture. Compound **3d** was less potent than CA-4, which in these experiments inhibited colchicine binding by 98%, while **3f**, **3h** and **3l** inhibited colchicine binding by only 21, 18 and 39%, respectively. 

In this small series of four compounds, the results obtained demonstrated that the antiproliferative activity was correlated with the inhibition of tubulin polymerization. Moreover, inhibition of tubulin assembly was correlated more closely with antiproliferative activity than with inhibition of [^3^H]colchicine binding.

#### 2.2.3. Molecular Modeling Studies

To rationalize the experimental data obtained for compounds **3d**, **3f**, **3h** and **3l**, a series of molecular docking simulations were performed. 

**Figure 3:**
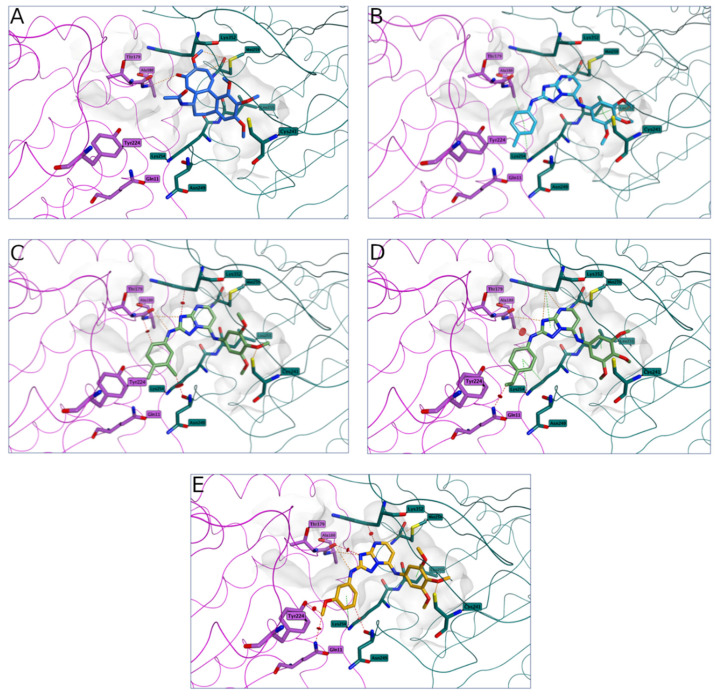
Colchicine binding conformation (**A**) and proposed binding for compounds **3d** (**B**), **3f** (**C**), **3h** (**D**) and **3l** (**E**) in the colchicine site. All the derivatives presented the trimethoxyphenyl ring in proximity to βCys241, while the substituted phenyl group at position 2 of the central core was sited at the interface between the two tubulin subunits, pointing toward a loop in the α-subunit (αSer178-αThr179). Compound **3d** presented two main interactions with βCys241, different anchoring contacts with the surrounding residues and no clashes with the tubulin structure. The rest of the compounds, even if conserving different interactions, including the important interaction with βCys241, presented different clashes with the surrounding residues, suggesting a non-optimal occupation of the colchicine site and indicating a reduced affinity for tubulin. The carbon atoms of the tubulin α unit residues are shown in lilac, while the carbon atoms of the β unit residues are represented in teal. Hydrogen bonds are shown as orange dashed lines, hydrophobic interactions as green dashed lines and distance clashes as red dashed lines.

The four derivatives occupy the tubulin colchicine site in a similar manner, mimicking the binding of the co-crystallized colchicine (Figure 3A–E), with the [1,2,4]triazolo[1,5-*a*]pyrimidine core lying on the central part of the binding site, the trimethoxyphenyl ring in position 7 placed in proximity of βCys241 and the differently substituted phenyl rings in position 2 sited at the interface between the two tubulin subunits, pointing toward a loop in the α-subunit (αSer178-αThr179). 

According to this binding site occupation prediction, the four derivatives would bind in this area of tubulin in a similar manner. Analyzing in more detail the interactions and any potential clash between the four molecules and the tubulin structure, some interesting observations were made.

The trimethoxyphenyl ring of **3d** presented two main interactions with βCys241 (Figure 3B), forming an important key contact point for inhibition of tubulin polymerization, and established different anchoring contacts with the surrounding residues (βLys254, βLeu255, βMet259, βLys352, αAla180), and these contacts further stabilized the compound–protein complex. Moreover, no clashes between the compound and the protein were present, indicating that **3d** had an optimal occupation of the colchicine site. This could then translate to **3d** being the best compound of the series in inhibiting tubulin polymerization and competing for the colchicine site. 

Introduction of a second methyl group in the *m*,*p*-xylyl derivative **3f** did not seem to affect the interactions with the tubulin residues, including interaction with βCys241 (Figure 3C), but the additional methyl group at the meta-position did present a possible sterical clash with βAsn249, and this could affect the compound–protein complex, reducing its stability. Moreover, to accommodate the second methyl group, **3f** had a slightly different binding orientation as compared with compound **3d**, and this caused other possible steric clashes with the surrounding residues (βAsn249, βLys352, αAla180). The presence of these potential issues suggests that **3f** could not have an optimal occupation of the colchicine site, hence a reduced affinity towards tubulin, and therefore a detrimental effect in the inhibition of tubulin polymerization. 

A similar effect was also observed for **3h** (*p*-ethyl, Figure 3D) and **3l** (*m*-methoxy, Figure 3E), where the longer ethyl chain in position 4′ and the presence of the methoxy groups in position 3′ for compounds **3h** and **3l**, respectively, caused some potential steric clashes with αGln11 and with αGln11/αTyr224, respectively. These negative structural features forced the compounds to adjust their binding, possibly reducing compound–protein complex stability. This could suggest a lower affinity for the binding site, negatively affecting the inhibition of tubulin polymerization. 

#### 2.2.4. Compound **3d** Induced G2/M Arrest of the Cell Cycle

**Figure 4:**
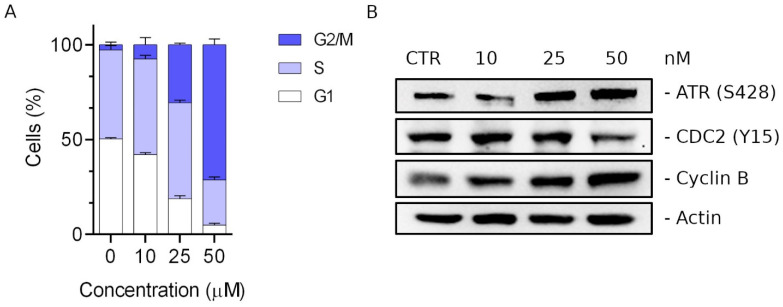
(Panel (**A**)) Quantitative analysis of cell cycle phase distribution in HeLa cells after a 24 h treatment with **3d**, at the concentrations of 10, 25 or 50 nM. Cells were stained with PI to analyze the DNA content by flow cytometry. Data are presented as means of three independent experiments ± SEM. (Panel (**B**)) Effects of compound **3d** on cell cycle regulatory proteins and DNA damage checkpoint proteins. HeLa cells were treated for 24 h with the indicated concentration of **3d**, and expression of ATR, cdc2 (Y15) and cyclin B was detected by Western blot analysis.

With the aim of evaluating the effects of compound **3d** on the cell cycle, we analyzed HeLa cells after treatment for 24 h at 10, 25 or 50 nM. As shown in Figure 4A, compound **3d** caused a significant G2/M arrest in a concentration-dependent manner. The increase in G2/M cells was evident at 25 nM and accompanied by a concomitant reduction in both G1 and S phase cells.

We then evaluated by Western blot analysis the effects of **3d** on the expression of several proteins regulating the mitotic checkpoint. When damage to the mitotic spindle occurs, the mitotic spindle assembly checkpoint is activated, causing an accumulation of cyclin B and the dephosphorylation of cdc2 [67,68]. We found (Figure 4B) that treatment with **3d** induced an increase in cyclin B expression and, at the same time, a significant reduction in cdc2 phosphorylation (Y15). 

The prolonged arrest in metaphase induces the activation of a DNA damage signal cascade that involves primarily ATR kinase, which becomes phosphorylated at Ser428. As shown in Figure 4B, we found a considerable increase in ATR phosphorylation (Ser428) [69], suggesting that **3d** induced the activation of the DNA damage signaling response with consequent accumulation of cyclin B and a block of cells in the G2/M phase.

#### 2.2.5. Compound **3d** Induced Apoptosis in HeLa Cells through Mitochondrial Depolarization

**Figure 5:**
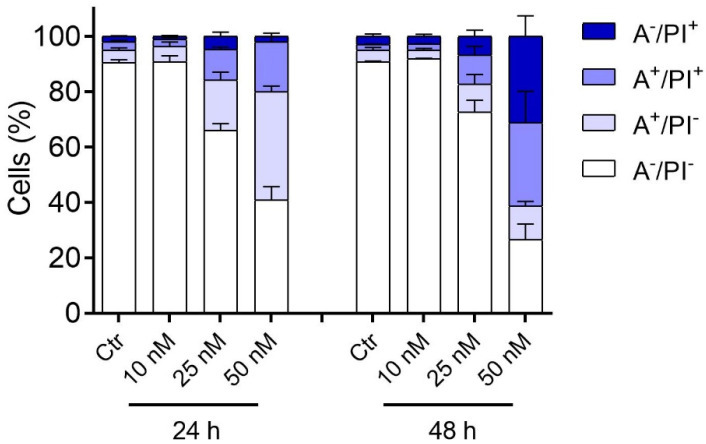
Apoptotic effects caused by **3d**. HeLa cells were treated with the indicated concentrations of **3d** for either 24 or 48 h and then were analyzed by flow cytometry after double staining with annexin-V-FITC and PI. A^−^/PI^−^ Alive cells; A^+^/PI^−^ Early apoptotic cells; A^+^/PI^+^ Late apoptotic cells; A^−^/PI^+^ Necrotic cells. In the histograms, data are represented as mean ± SEM of three independent experiments.

With the aim of evaluating the type of cell death induced by the treatment of compound **3d**, we treated HeLa cells with different concentrations of **3d,** and, after 24 or 48 h, we labeled the cells with both annexin-V conjugated with FITC and propidium iodide (PI). The cells were then subjected to flow cytometric analysis, and the results are shown in Figure 5. Compound **3d** induced, in a concentration- and time-dependent manner, apoptosis in the treated cells. The effect was evident at 25 nM and stronger still at 50 nM. With **3d** at 50 nM and at long incubation times, a fair percentage of cells (about 25%) became necrotic, being positive only for PI (A^−^/PI^+^).

Since many drugs acting as inhibitors of tubulin polymerization induce apoptosis through mitochondrial depolarization [70,71,72,73,74], we wanted to investigate whether **3d**-induced apoptosis was accompanied by a reduction in mitochondrial potential. To do this, we treated the Hela cells with the compound, and after 24 or 48 h we labeled the cells with the fluorescent dye JC-1.

**Figure 6:**
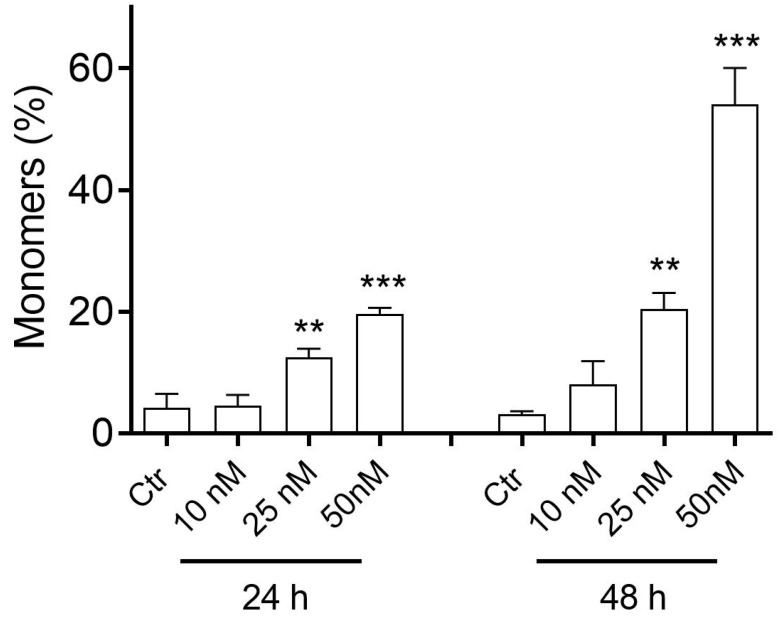
Assessment of mitochondrial depolarization after treatment with **3d**. HeLa cells were treated with the indicated concentrations of compound for 24 or 48 h. Data are represented as mean ± SEM of three independent experiments (** *p* < 0.01, *** *p* < 0.001).

In physiological conditions, JC-1 aggregates and emits red fluorescence, while, in depolarization conditions, the dye disaggregates and forms monomers that emit green fluorescence. As can be seen from Figure 6, after a 24 h treatment, a significant increase in the percentage of monomers was observed, and this percentage increased further at 48 h, in good agreement with the appearance of apoptotic cells described above.

#### 2.2.6. Compound **3d** Induces Caspase-9 Activation and Causes a Decrease in the Expression of Bcl-2 Protein

**Figure 7:**
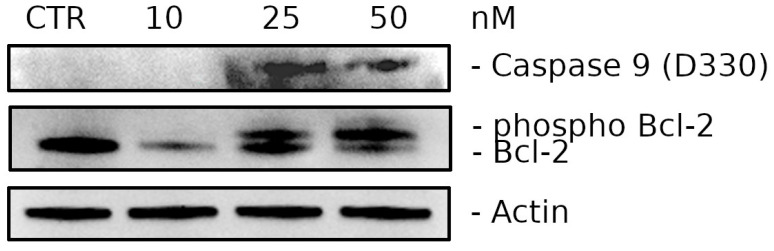
Western blot analysis of Caspase-9 (D330) (Cleaved form) and Bcl-2 levels. HeLa cells were treated for 24 h with **3d** at the indicated concentrations.

To further study the **3d**-induced apoptosis process we evaluated the expression of caspase-9. As shown in Figure 7, we observed at both 25 and 50 nM **3d** the appearance of the cleaved fragment of caspase-9. In this context, many studies have demonstrated that activation of initiator pro-caspase-9 causes a decrease in Bcl-2 expression, thus indicating the occurrence of apoptosis [75].

Western blot analysis showed that treatment with **3d** induced a reduction in Bcl-2 expression starting at 10 nM and, at higher concentrations, we also observed the appearance of the phosphorylated form of Bcl-2, a phenomenon frequently observed with antimitotic drugs and probably linked to mitotic death [76,77].

#### 2.2.7. Effects of **3d** Treatments on Zebrafish Embryos

**Figure 8:**
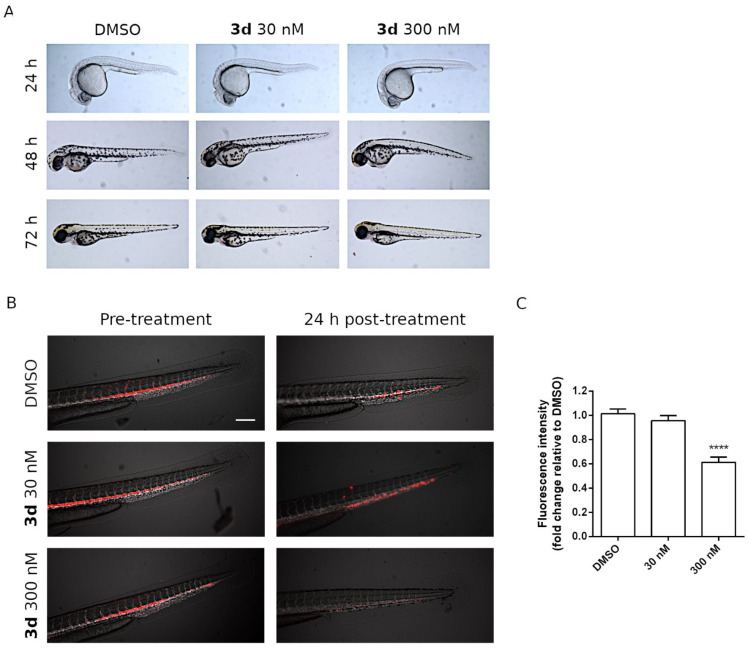
Effects of **3d** treatment on the zebrafish xenotransplantation model (**A**). Compound **3d** did not induce abnormal phenotypes or developmental anomalies in zebrafish embryos after 24, 48 and 72 h of incubation. DMSO-treated embryos were used as control. (**B**) Representative images of Tg(fli1:EGFP) zebrafish embryos (blood vessels shown in white) transplanted with DiI + HeLa cells (red). The embryos were treated with the indicated concentrations of **3d** for 24 h and then the fluorescence intensity was quantified as depicted in panel (**C**). Data are expressed as mean ± SD (**** *p* < 0.001). Scale bar, 200 μm. Effects of **3d** treatment on zebrafish embryos. No abnormal phenotypes or developmental defects were seen in comparison to DMSO-treated embryos (as a normal control) after 24, 48 and 72 h. (**B**) Effects of **3d** treatment on the zebrafish xenotransplantation model. Representative images of Tg(fli1:EGFP) zebrafish embryos (blood vessels shown in white) transplanted with DiI + HeLa cells (red). Embryos were treated for 24 h with DMSO (control group), 30 nM **3d** or 300 nM **3d**. (**C**) Histograms represent the fluorescence intensity of the tumor xenografts, indicating total HeLa cells present in each embryo after a 24 h treatment with **3d** at the indicated concentrations. Data are expressed as mean ± SD (**** *p* < 0.001). Scale bar, 200 μm.

To evaluate the toxicity of **3d** in vivo, wild-type embryos were exposed to **3d** diluted in fish water to 30 and 300 nM. Three replicates were performed, and, for each one, 20 embryos were used per drug concentration (60 embryos in total). The embryos were exposed to chemicals from the shield stage (6 h post-fertilization (hpf)) to larval stage (72 hpf), and phenotypical observations were recorded every 24 h. As shown in Figure 8A, no morphological abnormalities or embryonic lethality were observed in embryos treated with the 30 nM dose. The compound at 300 nM generated a small yolk sac edema in the majority of 48 hpf embryos (red arrow), and the edema was completely re-absorbed within the next 24 h in all treated animals, suggesting that **3d** was well tolerated by the embryos. 

#### 2.2.8. In Vivo Antitumor Activity of Compound **3d** in a Zebrafish Xenograft Model

To evaluate the effects of **3d** on tumor maintenance and dissemination in vivo, we took advantage of a zebrafish xenograft model. About 200 DiI-labeled HeLa cells were injected within the duct of Cuvier of 48 hpf zebrafish embryos. After xenotransplantation, the animals were treated with **3d** at 30 or 300 nM and circulating tumor cells were visualized and quantified in real time through fluorescence microscopy.

Upon 24 h treatment, HeLa cells were engrafted into the trunk and tail regions of DMSO-treated zebrafish embryos (controls). Interestingly, **3d** significantly reduced the number of disseminated cancer cells (Figure 8B), in agreement with in vitro cytotoxic effects of this compound on HeLa cancer cells. Notably, as shown in Figure 8C, the fluorescence intensity decreased in a dose-dependent manner, reaching statistical significance at 300 nM, indicating the effectiveness of **3d** in eradicating DiI-positive tumor cells.

## 3. Materials and Methods

### 3.1. Chemistry

The ^1^H and ^13^C NMR spectra were recorded on a Varian 400 Mercury Plus spectrometer. Chemical shifts (δ) are given in ppm upfield, and the spectra were recorded in appropriate deuterated solvents, as indicated. Mass spectra were recorded by an ESI single quadrupole mass spectrometer (Waters ZQ 2000; Waters Instruments, UK), and the values are expressed as [M+1]^+^. Melting points (mp) were determined on a Buchi-Tottoli apparatus and are uncorrected. All products reported showed ^1^H and ^13^C NMR spectra in agreement with the assigned structures. The purity of tested compounds was determined by combustion elemental analyses conducted by the Microanalytical Laboratory of the Chemistry Department of the University of Ferrara with a Yanagimoto MT-5 CHN recording elemental analyzer. All tested compounds yielded data consistent with a purity of at least 95% as compared with the theoretical values. Reaction courses and product mixtures were routinely monitored by TLC on silica gel (precoated F254 Merck plates), and compounds were visualized with aqueous KMnO_4_. Flash chromatography was performed using 230–400 mesh silica gel and the indicated solvent system. Organic solutions were dried over anhydrous Na_2_SO_4_.

#### 3.1.1. General Procedure A for the Synthesis of Compounds **8a–v**

To a solution of dimethyl cyanodithioimidocarbonate **7** (292 mg, 2 mmol) in isopropropanol (10 mL) was added the appropriate amine (2 mmol, 1 equiv.), and the mixture was refluxed for 18 h for the synthesis of compounds **8a–o**, while the mixture was stirred at room temperature (18 h) for the preparation of compounds **8p–v**. After this time, the solvent was removed under reduced pressure, the resulting residue was washed with diethyl ether (10 mL) and filtered to furnish the final compound **8a–v** used for the next reaction without any purification. For the characterization of compounds **8a**, **8c–f**, **8h** and **8j–n** see reference [53]. The characterization of compounds **8p–v** was described previously [78].

##### (Z)-Methyl *N*′-Cyano-*N*-(pyridin-3-yl)carbamimidothioate (**8b**)

Following general procedure A, compound **8b** was obtained as a white solid, yield 89%, mp 188–190 °C; ^1^H-NMR (DMSO-*d_6_*) δ: 2.62 (s, 3H), 7.10 (d, J = 8.0 Hz, 1H), 7.26 (m, 1H), 7.69 (s, 1H), 8.62 (d, J = 8.0 Hz, 1H), 10.0 (s, 1H). MS (ESI): [M+1]^+^ = 193.2.

##### (Z)-Methyl *N*′-Cyano-*N*-(3,5-dimethylphenyl)carbamimidothioate (**8g**)

Following general procedure A, compound **8g** was obtained as a white solid, yield 78%, mp 142–144 °C; ^1^H-NMR (DMSO-*d_6_*) δ: 2.22 (s, 6H), 2.44 (s, 3H), 6.36 (s, 1H), 7.10 (s, 2H), 10.0 (bs, 1H). MS (ESI): [M+1]^+^ = 220.3.

##### (Z)-Methyl *N*-(4-n-Propylphenyl)-*N*′-cyanocarbamimidothioate (**8i**)

Following general procedure A, compound **8i** was obtained as a white solid, yield 84%, mp 154–156 °C; ^1^H-NMR (DMSO-*d_6_*) δ: 0.93 (t, J = 7.2 Hz, 3H), 1.60 (m, 2H), 2.53 (s, 3H), 2.62 (t, J = 7.2 Hz, 2H), 7.20 (d, J = 8.4 Hz, 2H), 7.24 (d, J = 8.4 Hz, 2H), 10.1 (bs, 1H). MS (ESI): [M+1]^+^ = 234.3.

##### (Z)-Methyl *N*′-Cyano-*N*-(2,3-dihydrobenzo[b][1,4]dioxin-6-yl)carbamimidothioate (**8o**)

Following general procedure A, compound **8o** was obtained as a white solid, yield 92%, mp 202–204 °C; ^1^H-NMR (DMSO-*d_6_*) δ: 2.48 (s, 3H), 4.2 (s, 4H), 7.10 (d, J = 8.0 Hz, 1H), 6.84 (s, 2H), 6.94 (s, 1H), 10.0 (s, 1H). MS (ESI): [M+1]^+^ = 250.2

#### 3.1.2. General Procedure B for the Synthesis of Compounds **9a–v**

To a stirred suspension of compound **8a–v** (2 mmol) in methanol (10 mL) was added hydrazine monohydrate (0.2 mL, 4 mmol, 2 equiv.), and the mixture was heated under reflux for 18 h. After this time, the volatiles were removed, and the residue suspended with diethyl ether (10 mL) was sonicated for 10 min. The resultant solid was collected by filtration and then used for the next reaction without any purification. For the characterization of compounds **9a**, **9c–f**, **9h** and **9j–n** see reference [53].

##### *N*^3^-(Pyridin-3-yl)-1*H*-1,2,4-triazole-3,5-diamine (**9b**)

Following general procedure B, compound **9b** was obtained as a grey solid. Yield 68%, mp 178–179 °C; ^1^H-NMR (*d_6_*-DMSO) δ: 5.76 (bs, 2H), 7.31 (dd, J = 7.7 and 4.8 Hz, 1H), 7.71 (dt, J = 7.8 and 1.8 Hz, 1H), 8.12–8.21 (m. 1H), 8.33 (s, 1H), 8.52 (d, J = 1.8 Hz. 1H), 11.0 (br. s., 1H). MS (ESI): [M+1]^+^ = 177.2.

##### *N*^3^-(3,5-Dimethylphenyl)-1*H*-1,2,4-triazole-3,5-diamine (**9g**)

Following general procedure B, compound **9g** was obtained as a white solid. Yield 88%, mp 188–190 °C; ^1^H-NMR (*d_6_*-DMSO) δ: 2.15 (s, 6H), 5.76 (bs, 2H), 6.34 (s, 1H), 7.09 (s, 2H), 8.40 (s, 1H), 11.0 (bs, 1H). MS (ESI): [M+1]^+^ = 204.3.

##### *N*^3^-(4-n-Propylphenyl)-1*H*-1,2,4-triazole-3,5-diamine (**9i**)

Following general procedure B, compound **9i** was obtained as a white solid. Yield 82%, mp 186–188 °C; ^1^H-NMR (*d_6_*-DMSO) δ: 0.88 (t, J = 7.0 Hz, 3H), 1.48 (m, 2H), 2.41 (t, J = 7.6 Hz, 2H), 5.80 (bs, 2H), 6.94 (d, J = 8.2 Hz, 2H), 7.36 (d, J = 8.2 Hz, 2H), 8.43 (bs, 1H), 11.1 (bs, 1H). MS (ESI): [M+1]^+^ = 218.3.

##### *N*^3^-(2,3-Dihydrobenzo[b][1,4]dioxin-6-yl)-1*H*-1,2,4-triazole-3,5-diamine (**9o**)

Following general procedure B, compound **9o** was obtained as a white solid. Yield 82%, mp 202–204 °C; ^1^H-NMR (*d_6_*-DMSO) δ: 4.10–4.16 (m, 4H), 5.73 (bs, 2H), 6.60 (d, J = 8.8 Hz, 1H), 6.82 (dd, J = 8.8 and 2.4 Hz, 1H), 7.15 (d, J = 2.4 Hz, 1H), 8.31 (s, 1H), 10.8 (bs, 1H). MS (ESI): [M+1]^+^ = 234.2.

##### *N*^3^-Benzyl-1*H*-1,2,4-triazole-3,5-diamine (**9p**)

Following general procedure B, compound **9p** was obtained as a white solid. Yield 75%, mp 203–205 °C; ^1^H-NMR (*d_6_*-DMSO) δ: 4.21 (d, J = 6.4 Hz, 2H), 5.32 (bs, 2H), 6.12 (t, J = 6.4 Hz, 1H), 7.18–7.20 (m, 1H), 7.27–7.30 (m, 4H), 10.6 (bs, 1H). MS (ESI): [M+1]^+^ = 190.2.

##### *N*^3^-(4-Chlorobenzyl)-1*H*-1,2,4-triazole-3,5-diamine (**9q**)

Following general procedure B, compound **9q** was obtained as a white solid. Yield 88%, mp 176–178 °C; ^1^H-NMR (*d_6_*-DMSO) δ: 4.20 (d, J = 6.4 Hz, 2H), 5.30 (bs, 2H), 5.86 (t, J = 6.4 Hz, 1H), 7.29-7.38 (m, 4H), 10.8 (bs, 1H). MS (ESI): [M+1]^+^ = 224.7.

##### *N*^3^-(4-Methylbenzyl)-1*H*-1,2,4-triazole-3,5-diamine (**9r**)

Following general procedure B, compound **9r** was obtained as a white solid. Yield 87%, mp 188–190 °C; ^1^H-NMR (*d_6_*-DMSO) δ: 2.25 (s, 3H), 4.14 (d, J = 6.2 Hz, 2H), 5.27 (bs, 2H), 5.85 (t, J = 6.2 Hz, 1H), 7.05 (d, J = 8.0 Hz, 2H), 7.16 (d, J = 8.0 Hz, 2H), 10.7 (bs, 1H). MS (ESI): [M+1]^+^ = 203.9.

##### *N*^3^-(4-Methoxybenzyl)-1*H*-1,2,4-triazole-3,5-diamine (**9s**)

Following general procedure B, compound **9s** was obtained as a white solid. Yield 88%, mp 198–200 °C; ^1^H-NMR (*d_6_*-DMSO) δ: 3.71 (s, 3H), 4.11 (d, J = 6.4 Hz, 2H), 5.28 (bs, 2H), 5.92 (t, J = 6.4 Hz, 1H), 6.82 (d, J = 8.6 Hz, 2H), 7.20 (d, J = 8.6 Hz, 2H), 10.8 (bs, 1H). MS (ESI): [M+1]^+^ = 220.2.

##### *N*^3^-(Benzo[d][1,3]dioxol-5-ylmethyl)-1*H*-1,2,4-triazole-3,5-diamine (**9t**)

Following general procedure B, compound **9t** was obtained as a white solid. Yield 93%, mp 204–206 °C; ^1^H-NMR (*d_6_*-DMSO) δ: 4.12 (d, J = 6.4 Hz, 2H), 4,24 (bs, 1H), 5.38 (bs, 2H), 5.97 (s, 2H), 6.74 (dd, J = 8.2 and 2.2 Hz, 1H), 6.80 (d, J = 8.2 Hz, 1H), 6.88 (d, J = 2.2 Hz, 1H), 10.8 (bs, 1H). MS (ESI): [M+1]^+^ = 234.2.

##### *N*^3^-(2-Phenylethyl)-1*H*-1,2,4-triazole-3,5-diamine (**9u**)

Following general procedure B, compound **9u** was obtained as a white solid. Yield 90%, mp 206–208 °C; ^1^H-NMR (*d_6_*-DMSO) δ: 2.82 (t, J = 7.2 Hz, 2H), 3.35–3.37 (m, 2H), 5.30 (bs, 2H), 6.22 (t, J = 6.4 Hz, 1H), 7.19–7.22 (m, 2H), 7.27–7.30 (m, 3H), 10.6 (bs, 1H). MS (ESI): [M+1]^+^ = 204.2.

##### *N*^3^-(3-Phenylpropyl)-1*H*-1,2,4-triazole-3,5-diamine (**9v**)

Following general procedure B, compound **9v** was obtained as a white solid. Yield 92%, mp 188–190 °C; ^1^H-NMR (*d_6_*-DMSO) δ: 1.74 (m, 2H), 2.55 (t, J = 7.2 Hz, 2H), 2.95–2.99 (m, 2H), 5.32 (bs, 2H), 6.14 (bs, 1H), 7.14–7.18 (m, 3H), 7.22–7.25 (m, 2H), 10.6 (bs, 1H). MS (ESI): [M+1]^+^ = 218.3.

#### 3.1.3. General Procedure C for the Synthesis of Compounds **3a–v**

To a solution of (*E*)-3-(dimethylamino)-1-(3,4,5-trimethoxyphenyl)prop-2-en-1-one **10** (132 mg, 0.5 mmol) in glacial acetic acid (3 mL) was added the appropriate 3-substituted 5-amino-1,2,4-triazole **9a–v** (0.75 mmol, 1.5 equiv.), and the resulting mixtures were stirred for 2 h at 80 °C and then evaporated to dryness in vacuo. The residue was portioned between dichloromethane and a 2 M solution of Na_2_CO_3_ in water, the organic phase was separated, washed with brine, dried over sodium sulfate, filtered and concentrated. The crude residue was subjected to column chromatography on silica gel using a mixture of ethyl acetate–methanol as eluent or recrystallized from diethyl ether, yielding the appropriate 2-substituted-7-(3′,4′5′-trimethoxyphenyl)[1,2,4]triazolo[1,5-*a*]pyrimidines **3a–v**. 

##### *N*-Phenyl-7-(3,4,5-trimethoxyphenyl)-[1,2,4]triazolo[1,5-*a*]pyrimidin-2-amine (**3a**)

Following general procedure C, the crude residue was purified by crystallization with diethyl ether to furnish **3a** as a yellow solid. Yield: 49%, mp 235–236 °C; ^1^H-NMR (DMSO-*d_6_*) δ: 3.79 (s, 3H), 3.91 (s, 6H), 6.94 (t, J = 8.4 Hz, 1H), 7.27 (t, J = 8.4 Hz, 2H), 7.52 (d, J = 4.8 Hz, 1H), 7.71 (s, 2H), 7.73 (t, J = 8.4 Hz, 2H), 8.66 (d, J = 4.8 Hz, 1H), 9.89 (s, 1H); ^13^C-NMR (DMSO-*d_6_*) δ: 56.60 (2C), 60.71, 107.70 (2C), 108.21, 117.39 (2C), 121.15, 125.36, 129.17 (2C), 140.69, 141.18, 145.52, 153.17 (2C), 153.36, 155.71, 163.57. MS (ESI): [M+1]^+^ = 378.4. Anal. calcd for C_20_H_19_N_5_O_3_. C, 63.65; H, 5.07; N, 18.56; found: C, 63.48; H, 4.88; N, 18.34.

##### *N*-(Pyridin-3-yl)-7-(3,4,5-trimethoxyphenyl)-[1,2,4]triazolo[1,5-*a*]pyrimidin-2-amine (**3b**)

Following general procedure C, the crude residue was purified by crystallization with diethyl ether to furnish **3b** as a brown solid. Yield: 47%, mp 172–174 °C; ^1^H-NMR (DMSO-*d_6_*) δ: 3.79 (s, 3H), 3.81 (s, 6H), 7.31 (dd, J = 8.4 and 8.0 Hz, 1H), 7.55 (d, J = 4.8 Hz, 1H), 7.56 (s, 2H), 8.08–8.09 (m, 1H), 8.10–8.14 (m, 1H), 8.70 (d, J = 4.8 Hz, 1H), 8.97 (d, J = 2.8 Hz, 1H), 10.1 (s, 1H); ^13^C-NMR (DMSO-*d_6_*) δ: 56.62 (2C), 60.72, 107.69 (2C), 108.54, 123.78, 123.94, 125.27, 137.88, 139.51, 140.72, 142.18, 145.77, 153.19 (2C), 153.66, 155.75, 163.23. MS (ESI): [M+1]^+^ = 379.3. Anal. calcd for C_19_H_18_N_6_O_3_. C, 60.31; H, 4.79; N, 22.21; found: C, 60.12; H, 4.67; N, 22.03.

##### *N*-(4-Fluorophenyl)-7-(3,4,5-trimethoxyphenyl)-[1,2,4]triazolo[1,5-*a*]pyrimidin-2-amine (**3c**)

Following general procedure C, the crude residue was purified by crystallization with diethyl ether to furnish **3c** as a white solid. Yield: 58%, mp 234–235 °C; ^1^H-NMR (DMSO-*d_6_*) δ: 3.78 (s, 3H), 3.90 (s, 6H), 7.12 (t, J = 8.8 Hz, 2H), 7.50 (d, J = 4.8 Hz, 1H), 7.66 (s, 2H), 7.66–7.73 (m, 2H), 8.66 (d, J = 4.8 Hz, 1H), 9.91 (s, 1H); ^13^C-NMR (DMSO-*d_6_*) δ: 56.62 (2C), 60.71, 107.66 (2C), 108.30, 115.60 and 115.82 (J_2CF_ = 22.1 Hz, 2C), 118.75 and 118.82 (J_3CF_ = 6.9 Hz, 2C), 125.35, 137.67, 140.67, 145.57, 153.19 (2C), 153.36, 155.74, 155.95 and 158.31 (J_1CF_ = 236 Hz, 1C), 163.54. MS (ESI): [M+1]^+^ = 396.2. Anal. calcd for C_20_H_18_FN_5_O_3_. C, 60.75; H, 4.59; N, 17.71; found: C, 60.56; H, 4.43; N, 17.48.

##### *N*-(p-Tolyl)-7-(3,4,5-trimethoxyphenyl)-[1,2,4]triazolo[1,5-*a*]pyrimidin-2-amine (**3d**)

Following general procedure C, the crude residue was crystallized with diethyl ether to furnish **3d** as a yellow solid. Yield: 58%, mp 208–209 °C; ^1^H-NMR (DMSO-*d_6_*) δ: 2.23 (s, 3H), 3.79 (s, 3H), 3.91 (s, 6H), 7.06 (d, J = 8.4 Hz, 2H), 7.50 (d, J = 4.8 Hz, 1H), 7.59 (d, J = 8.4 Hz, 2H), 7.68 (s, 2H), 8.64 (d, J = 4.8 Hz, 1H), 9.57 (s, 1H); ^13^C-NMR (DMSO-*d_6_*) δ: 20.77, 56.60 (2C), 60.71, 107.68 (2C), 108.09, 117.43 (2C), 125.39, 129.56 (2C), 129.90, 138.69, 140.66, 145.42, 153.16 (2C), 153.23, 155.74, 163.81. MS (ESI): [M+1]^+^ = 392.4. Anal. calcd for C_21_H_21_N_5_O_3_. C, 64.44; H, 5.41; N, 17.89; found: C, 64.31; H, 5.18; N, 17.77.

##### *N*-(m-Tolyl)-7-(3,4,5-trimethoxyphenyl)-[1,2,4]triazolo[1,5-*a*]pyrimidin-2-amine (**3e**)

Following general procedure C, the crude residue was purified by crystallization with diethyl ether to furnish **3e** as a yellow solid. Yield: 62%, mp 229–230 °C; ^1^H-NMR (DMSO-*d_6_*) δ: 2.26 (s, 3H), 3.78 (s, 3H), 3.89 (s, 6H), 6.74 (d, J = 8.0 Hz, 1H), 7.14 (t, J = 8.0 Hz, 1H), 7.46 (d, J = 2.0 Hz, 1H), 7.48 (d, J = 4.8 Hz, 1H), 7.60 (d, J = 8.0 Hz, 1H), 7.65 (s, 2H), 8.66 (d, J = 4.8 Hz, 1H), 9.81 (s, 1H); ^13^C-NMR (DMSO-*d_6_*) δ: 21.79, 56.65 (2C), 60.71, 107.64 (2C), 108.22, 114.57, 117.97, 121.94, 125.44, 128.98, 138.32, 140.65, 141.14, 145.58, 153.17 (2C), 153.31, 155.67, 163.61. MS (ESI): [M+1]^+^ = 392.6. Anal. calcd for C_21_H_21_N_5_O_3_. C, 64.44; H, 5.41; N, 17.89; found: C, 64.23; H, 5.31; N, 17.69.

##### *N*-(3,4-Dimethylphenyl)-7-(3,4,5-trimethoxyphenyl)-[1,2,4]triazolo[1,5-*a*]pyrimidin-2-amine (**3f**)

Following general procedure C, the crude residue was crystallized with diethyl ether to furnish **3f** as a yellow solid. Yield: 54%, mp 189–190 °C; ^1^H-NMR (DMSO-*d_6_*) δ: 2.14 (s, 3H), 2.16 (s, 3H), 3.78 (s, 3H), 3.89 (s, 6H), 7.02 (d, J = 8.4 Hz, 1H), 7.41 (d, J = 1.2 Hz, 1H), 7.47 (d, J = 4.8 Hz, 1H), 7.54 (dd, J = 8.4 and 1.2 Hz, 1H), 7.65 (s, 2H), 8.64 (d, J = 4.8 Hz, 1H), 9.68 (s, 1H); ^13^C-NMR (DMSO-*d_6_*) δ: 19.08, 20.24, 56.64 (2C), 60.70, 107.73 (2C), 108.08, 114.86, 118.81, 125.47, 128.72, 130.01, 138.74, 138.97, 140.62, 145.47, 153.17 (2C), 155.71, 158.64, 163.73. MS (ESI): [M+1]^+^ = 406.4. Anal. calcd for C_22_H_23_N_5_O_3_. C, 65.17; H, 5.72; N, 17.27; found: C, 64.92; H, 5.53; N, 17.05.

##### *N*-(3,5-Dimethylphenyl)-7-(3,4,5-trimethoxyphenyl)-[1,2,4]triazolo[1,5-*a*]pyrimidin-2-amine (**3g**)

Following general procedure C, the crude residue was purified by flash chromatography, using ethyl acetate as eluent, to furnish **3g** as a yellow solid. Yield: 58%, mp 228–230 °C; ^1^H-NMR (DMSO-*d_6_*) δ: 2.21 (s, 6H), 3.77 (s, 3H), 3.87 (s, 6H), 6.55 (s, 1H), 7.32 (s, 2H), 7.44 (d, J = 4.8 Hz, 1H), 7.60 (s, 2H), 8.65 (d, J = 4.8 Hz, 1H), 9.72 (s, 1H); ^13^C-NMR (DMSO-*d_6_*) δ: 21.15 (2C), 56.13 (2C), 60.13, 107.22 (2C), 107.86, 114.64 (2C), 122.27, 124.98, 137.51 (2C), 140.01, 140.53, 145.10, 152.61 (2C), 152.71, 155.06, 163.07. MS (ESI): [M+1]^+^ = 406.4. Anal. calcd for C_22_H_23_N_5_O_3_. C, 65.17; H, 5.72; N, 17.27; found: C, 64.97; H, 5.52; N, 17.11.

##### *N*-(4-Ethylphenyl)-7-(3,4,5-trimethoxyphenyl)-[1,2,4]triazolo[1,5-*a*]pyrimidin-2-amine (**3h**)

Following general procedure C, the crude residue crystallized with diethyl ether furnished **3h** as a yellow solid. Yield: 63%, mp 199–200 °C; ^1^H-NMR (DMSO-*d_6_*) δ: 1.14 (t, J = 8.4 Hz, 3H), 2.52 (q, J = 8.4 Hz, 2H), 3.79 (s, 3H), 3.91 (s, 6H), 7.09 (d, J = 8.4 Hz, 2H), 7.50 (d, J = 4.8 Hz, 1H), 7.61 (d, J = 8.4 Hz, 2H), 7.69 (s, 2H), 8.64 (d, J = 4.8 Hz, 1H), 9.77 (s, 1H); ^13^C-NMR (DMSO-*d_6_*) δ: 16.34, 27.93, 56.59 (2C), 60.72, 107.68 (2C), 108.09, 117.56 (2C), 125.38, 128.16, 128.36 (2C), 136.52, 138.89, 140.66, 145.41, 153.16 (2C), 155.75, 163.71. MS (ESI): [M+1]^+^ = 406.3. Anal. calcd for C_22_H_23_N_5_O_3_. C, 65.17; H, 5.72; N, 17.27; found: C, 64.91; H, 5.54; N, 17.12.

##### *N*-(4-Propylphenyl)-7-(3,4,5-trimethoxyphenyl)-[1,2,4]triazolo[1,5-*a*]pyrimidin-2-amine (**3i**)

Following general procedure C, the crude residue crystallized with diethyl ether furnished **3i** as a yellow solid. Yield: 64%, mp 185–187 °C; ^1^H-NMR (DMSO-*d_6_*) δ: 0.86 (t, J = 7.2 Hz, 3H), 1.52–1.55 (m, 2H), 2.52 (q, J = 7.2 Hz, 2H), 3.78 (s, 3H), 3.91 (s, 6H), 7.07 (d, J = 8.4 Hz, 2H), 7.50 (d, J = 4.8 Hz, 1H), 7.60 (d, J = 8.4 Hz, 2H), 7.69 (s, 2H), 8.64 (d, J = 4.8 Hz, 1H), 9.77 (s, 1H); ^13^C-NMR (DMSO-*d_6_*) δ: 14.04, 24.75, 37.05, 56.59 (2C), 60.71, 107.68 (2C), 108.08, 117.50 (2C), 125.38, 128.95 (2C), 134.88, 138.90, 140.66, 145.40, 147.92, 153.16 (2C), 155.75, 163.71. MS (ESI): [M+1]^+^ = 420.4. Anal. calcd for C_23_H_25_N_5_O_3_. C, 65.85; H, 6.01; N, 16.70; found: C, 65.69; H, 5.88; N, 16.52.

##### *N*-(4-Isopropylphenyl)-7-(3,4,5-trimethoxyphenyl)-[1,2,4]triazolo[1,5-*a*]pyrimidin-2-amine (**3j**) 

Following general procedure C, the crude residue crystallized with diethyl ether furnished **3j** as a yellow solid. Yield: 53%, mp 212–214 °C; ^1^H-NMR (DMSO-*d_6_*) δ: 1.12 (d, J = 6.8 Hz, 6H), 2.46–2.48 (m, 1H), 3.79 (s, 3H), 3.91 (s, 6H), 7.12 (d, J = 8.4 Hz, 2H), 7.50 (d, J = 4.8 Hz, 1H), 7.61 (d, J = 8.4 Hz, 2H), 7.69 (s, 2H), 8.64 (d, J = 4.8 Hz, 1H), 9.76 (s, 1H); ^13^C-NMR (DMSO-*d_6_*) δ: 24.52 (2C), 33.17, 56.59 (2C), 60.71, 107.69 (2C), 108.08, 115.95, 117.63 (2C), 125.38, 126.84 (2C), 138.95, 140.65, 141.25, 145.38, 153.16 (2C), 155.77, 163.73. MS (ESI): [M+1]^+^ = 420.5. Anal. calcd for C_23_H_25_N_5_O_3_. C, 65.85; H, 6.01; N, 16.70; found: C, 65.70; H, 5.88; N, 16.51.

##### *N*-(4-Methoxyphenyl)-7-(3,4,5-trimethoxyphenyl)-[1,2,4]triazolo[1,5-*a*]pyrimidin-2-amine (**3k**)

Following general procedure C, the crude residue was purified by crystallization with diethyl ether to furnish **3k** as a yellow solid. Yield: 62%, mp 213–215 °C; ^1^H-NMR (DMSO-*d_6_*) δ: 3.70 (s, 3H), 3.78 (s, 3H), 3.90 (s, 6H), 7.48 (d, J = 4.8 Hz, 1H), 6.85 (d, J = 8.8 Hz, 2H), 7.61 (d, J = 8.8 Hz, 2H), 7.68 (s, 2H), 8.62 (d, J = 4.8 Hz, 1H), 9.65 (s, 1H); ^13^C-NMR (DMSO-*d_6_*) δ: 55.69, 56.60 (2C), 60.71, 107.65 (2C), 107.98, 114.45 (2C), 118.90 (2C), 125.40, 134.54, 140.63, 145.30, 153.08, 153.16 (2C), 154.15, 155.82, 163.85. MS (ESI): [M+1]^+^ = 408.4. Anal. calcd for C_21_H_21_N_5_O_4_. C, 61.91; H, 5.20; N, 17.19; found: C, 61.77; H, 5.02; N, 17.01.

##### *N*-(3-Methoxyphenyl)-7-(3,4,5-trimethoxyphenyl)-[1,2,4]triazolo[1,5-*a*]pyrimidin-2-amine (**3l**) 

Following general procedure C, the crude residue was purified by crystallization with diethyl ether to furnish **3l** as a yellow solid. Yield: 54%, mp 165–166 °C; ^1^H-NMR (DMSO-*d_6_*) δ: 3.70 (s, 3H), 3.78 (s, 3H), 3.90 (s, 6H), 6.54 (dd, J = 8.0 and 2.0 Hz, 1H), 7.19 (t, J = 8.4 Hz, 1H), 7.24 (dd, J = 8.0 and 2.0 Hz, 1H), 7.40 (d, J = 2.0 Hz, 1H), 7.49 (d, J = 4.8 Hz, 1H), 7.65 (s, 2H), 8.66 (d, J = 4.8 Hz, 1H), 9.88 (s, 1H); ^13^C-NMR (DMSO-*d_6_*) δ: 55.35, 56.59 (2C), 60.70, 103.88, 105.92, 107.69 (2C), 108.31, 110.17, 125.42, 129.92, 140.66, 142.29, 145.69, 153.19 (2C), 153.43, 155.61, 160.33, 163.48. MS (ESI): [M+1]^+^ = 408.3. Anal. calcd for C_21_H_21_N_5_O_4_. C, 61.91; H, 5.20; N, 17.19; found: C, 61.72; H, 5.10; N, 17.02.

##### *N*-(4-Ethoxyphenyl)-7-(3,4,5-trimethoxyphenyl)-[1,2,4]triazolo[1,5-*a*]pyrimidin-2-amine (**3m**)

Following general procedure C, the crude residue was purified by crystallization with diethyl ether to furnish **3m** as a yellow solid. Yield: 61%, mp 205–207 °C; ^1^H-NMR (DMSO-*d_6_*) δ: 1.28 (t, J = 6.8 Hz, 3H), 3.78 (s, 3H), 3.90 (s, 6H), 3.96 (q, J = 6.8 Hz, 2H), 6.83 (d, J = 9.2 Hz, 2H), 7.48 (d, J = 4.8 Hz, 1H), 7.60 (d, J = 8.8 Hz, 2H), 7.68 (s, 2H), 8.62 (d, J = 4.8 Hz, 1H), 9.65 (s, 1H); ^13^C-NMR (DMSO-*d_6_*) δ: 15.28, 56.59 (2C), 60.70, 63.60, 107.65 (2C), 107.97, 114.92, 115.03, 117.04, 118.88 (2C), 125.41, 134.45, 140.63, 145.29, 153.08, 153.16, 153.39, 155.82, 163.85. MS (ESI): [M+1]^+^ = 422.4. Anal. calcd for C_22_H_23_N_5_O_4_. C, 62.70; H, 5.50; N, 16.62; found: C, 62.58; H, 5.38; N, 16.49.

##### *N*-(Benzo[d][1,3]dioxol-5-yl)-7-(3,4,5-trimethoxyphenyl)-[1,2,4]triazolo[1,5-*a*]pyrimidin-2-amine (**3n**)

Following general procedure C, the crude residue purified by crystallization with diethyl ether furnished **3n** as a white solid. Yield: 63%, mp 234–236 °C; ^1^H-NMR (DMSO-*d_6_*) δ: 3.78 (s, 3H), 3.90 (s, 6H), 5.95 (s, 2H), 6.82 (d, J = 8.4 Hz, 1H), 7.03 (dd, J = 8.4 and 2.0 Hz, 1H), 7.48-7.50 (m, 2H), 7.66 (s, 2H), 8.64 (d, J = 4.8 Hz, 1H), 9.76 (s, 1H); ^13^C-NMR (DMSO-*d_6_*) δ: 56.62 (2C), 60.70, 99.91, 101.24, 107.64 (2C), 108.11, 108.65, 109.97, 125.37, 135.89, 140.65, 141.62, 145.44, 147.78, 153.17 (2C), 153.27, 155.71, 163.64. MS (ESI): [M+1]^+^ = 422.4. Anal. calcd for C_21_H_19_N_5_O_3_. C, 59.85; H, 4.54; N, 16.62; found: C, 59.72; H, 4.33; N, 16.48.

##### *N*-(2,3-Dihydrobenzo[b][1,4]dioxin-6-yl)-7-(3,4,5-trimethoxyphenyl)-[1,2,4]triazolo[1,5-*a*]pyrimidin-2-amine (**3o**)

Following general procedure C, the crude residue purified by crystallization with diethyl ether furnished **3o** as a white solid. Yield: 58%, mp 212–214 °C; ^1^H-NMR (DMSO-*d_6_*) δ: 3.78 (s, 3H), 3.91 (s, 6H), 4.16–4.22 (m, 4H), 6.74 (d, J = 8.4 Hz, 1H), 7.02 (dd, J = 8.4 and 2.4 Hz, 1H), 7.39 (d, J = 2.8 Hz, 1H), 7.48 (d, J = 4.8 Hz, 1H), 7.66 (s, 2H), 8.63 (d, J = 4.8 Hz, 1H), 9.67 (s, 1H); ^13^C-NMR (DMSO-*d_6_*) δ: 56.58 (2C), 60.69, 64.32, 64.74, 86.40, 106.42, 107.59 (2C), 108.06, 110.89, 117.31, 125.42, 135.04, 138.01, 140.62, 143.63, 145.44, 153.19 (2C), 155.71, 163.63. MS (ESI): [M+1]^+^ = 436.4. Anal. calcd for C_22_H_21_N_5_O_5_. C, 60.68; H, 4.86; N, 16.08; found: C, 60.54; H, 4.72; N, 15.98. 

##### *N*-Benzyl-7-(3,4,5-trimethoxyphenyl)-[1,2,4]triazolo[1,5-*a*]pyrimidin-2-amine (**3p**)

Following general procedure C, the crude residue was purified by flash chromatography, using ethyl acetate:methanol 9.5:0.5 as eluent, to furnish **3p** as a white solid. Yield: 58%, mp 144–146 °C; ^1^H-NMR (DMSO-*d_6_*) δ: 3.73 (s, 3H), 3.76 (s, 6H), 4.48 (d, J = 6.0 Hz, 2H), 7.22 (t, J = 8.4 Hz, 1H), 7.29 (t, J = 8.4 Hz, 2H), 7.34 (d, J = 8.4 Hz, 2H), 7.38 (d, J = 5.2 Hz, 1H), 7.59 (s, 2H), 7.61 (t, J = 6.4 Hz, 1H), 8.51 (d, J = 5.2 Hz, 1H); ^13^C-NMR (DMSO-*d_6_*) δ: 45.85, 56.49 (2C), 60.63, 107.38, 107.64 (2C), 125.45, 127.03, 127.29 (2C), 128.66 (2C), 140.44, 140.64, 144.61, 152.04, 153.02 (2C), 156.73, 167.43. MS (ESI): [M+1]^+^ = 392.4. Anal. calcd for C_21_H_21_N_5_O_3_. C, 64.44; H, 5.41; N, 17.89; found: C, 64.24; H, 5.16; N, 17.67.

##### *N*-(4-Chlorobenzyl)-7-(3,4,5-trimethoxyphenyl)-[1,2,4]triazolo[1,5-*a*]pyrimidin-2-amine (**3q**)

Following general procedure C, the crude residue was purified by flash chromatography, using ethyl acetate:methanol 9:1 as eluent, to furnish compound **3q** as a yellow solid. Yield: 58%, mp 171–173 °C; ^1^H-NMR (DMSO-*d_6_*) δ: 3.76 (s, 6H), 3.81 (s, 3H), 4.47 (d, J = 6.4 Hz, 2H), 7.33–7.35 (m, 4H), 7.38 (d, J = 5.2 Hz, 1H), 7.57 (s, 2H), 7.68 (t, J = 6.4 Hz, 1H), 8.52 (d, J = 5.2 Hz, 1H); ^13^C-NMR (DMSO-*d_6_*) δ: 45.22, 56.48 (2C), 60.64, 107.48, 107.64 (2C), 125.42, 128.60 (2C), 129.14 (2C), 131.55, 139.73, 140,12, 144.70, 152.13 (2C), 153.03, 156.72, 167.30. MS (ESI): [M+1]^+^ = 426.4. Anal. calcd for C_21_H_20_ClN_5_O_3_. C, 59.23; H, 4.73; N, 16.44; found: C, 59.11; H, 4.60; N, 16.33.

##### *N*-(4-Methylbenzyl)-7-(3,4,5-trimethoxyphenyl)-[1,2,4]triazolo[1,5-*a*]pyrimidin-2-amine (**3r**)

Following general procedure C, using ethyl acetate:methanol 9.5:0.5 as eluent, compound **3r** was obtained as a white solid. Yield: 61%, mp 156–158 °C; ^1^H-NMR (DMSO-*d_6_*) δ: 2.23 (s, 3H), 3.74 (s, 3H), 3.77 (s, 6H), 4.43 (d, J = 6.0 Hz, 2H), 7.07 (d, J = 8.0 Hz, 2H), 7.21 (d, J = 8.0 Hz, 2H), 7.38 (d, J = 4.8 Hz, 1H), 7.54 (t, J = 6.4 Hz, 1H), 7.59 (s, 2H), 8.51 (d, J = 4.8 Hz, 1H); ^13^C-NMR (DMSO-*d_6_*) δ: 20.61, 45.15, 56.02 (2C), 60.17, 106.88, 107.17 (2C), 125.00, 126.83 (2C), 128.72 (2C), 135.56, 137.09, 139.96, 144.11, 151.54, 152.57 (2C), 156.25, 166.95. MS (ESI): [M+1]^+^ = 406.3. Anal. calcd for C_22_H_23_N_5_O_3_. C, 65.17; H, 5.72; N, 17.27; found: C, 65.03; H, 5.58; N, 17.18.

##### *N*-(4-Methoxybenzyl)-7-(3,4,5-trimethoxyphenyl)-[1,2,4]triazolo[1,5-*a*]pyrimidin-2-amine (**3s**)

Following general procedure C, the crude residue was purified by flash chromatography, using ethyl acetate:methanol 9.5:0.5 as eluent, to furnish **3s** as a white solid. Yield: 66%, mp 190–192 °C; ^1^H-NMR (DMSO-*d_6_*) δ: 3.69 (s, 3H), 3.74 (s, 3H), 3.79 (s, 6H), 4.40 (d, J = 6.4 Hz, 2H), 6.83 (d, J = 8.4 Hz, 2H), 7.26 (d, J = 8.4 Hz, 2H), 7.38 (d, J = 4.8 Hz, 1H), 7.53 (t, J = 6.4 Hz, 1H), 7.61 (s, 2H), 8.30 (d, J = 4.8 Hz, 1H); ^13^C-NMR (DMSO-*d_6_*) δ: 45.33, 55.46, 56.52 (2C), 60.64, 107.35, 107.67 (2C), 114.06 (2C), 125.48, 128.65 (2C), 132.53, 140.44, 144.57, 151.98, 153.03 (2C), 156.71, 158.56, 167.41. MS (ESI): [M+1]^+^ = 422.4. Anal. calcd for C_22_H_23_N_5_O_4_. C, 62.70; H, 5.50; N, 16.62; found: C, 62.55; H, 5.36; N, 16.44.

##### *N*-(Benzo[d][1,3]dioxol-5-ylmethyl)-7-(3,4,5-trimethoxyphenyl)-[1,2,4]triazolo[1,5-*a*]pyrimidin-2-amine (**3t**)

Following general procedure C, the crude residue purified by flash chromatography, using ethyl acetate:methanol 9.5:0.5 as eluent, furnished **3t** as a white solid. Yield: 61%, mp 233–235 °C; ^1^H-NMR (DMSO-*d_6_*) δ: 3.74 (s, 3H), 3.79 (s, 6H), 4.38 (d, J = 6.0 Hz, 2H), 5.94 (s, 2H), 6.81 (s, 2H), 6.92 (s, 1H), 7.37 (d, J = 5.2 Hz, 1H), 7.57 (t, J = 8.4 Hz, 1H), 7.60 (s, 2H), 8.51 (d, J = 5.2 Hz, 1H); ^13^C-NMR (DMSO-*d_6_*) δ: 45.68, 56.51 (2C), 60.64, 101.19, 107.42, 107.65 (2C), 108.02, 108.41, 120.44, 125.49, 134.59, 140.45, 144.64, 146.37, 147.62, 152.03, 153.04 (2C), 156.70, 167.33. MS (ESI): [M+1]^+^ = 436.4. Anal. calcd for C_22_H_21_N_5_O_5_. C, 60.68; H, 4.86; N, 16.08; found: C, 60.60; H, 4.77; N, 15.88.

##### *N*-Phenethyl-7-(3,4,5-trimethoxyphenyl)-[1,2,4]triazolo[1,5-*a*]pyrimidin-2-amine (**3u**)

Following general procedure C, the crude residue was purified by flash chromatography, using ethyl acetate:methanol 9.5:0.5 as eluent, to furnish **3u** as a white solid. Yield: 59%, mp 138–139 °C; ^1^H-NMR (DMSO-*d_6_*) δ: 2.44 (t, J = 7.6 Hz, 2H), 3.42-3.46 (m, 2H), 3.76 (s, 3H), 3.84 (s, 6H), 7.14 (t, J = 8.4 Hz, 1H), 7.23–7.27 (m, 5H), 7.38 (d, J = 4.8 Hz, 1H), 7.66 (s, 2H), 8.51 (d, J = 4.8 Hz, 1H); ^13^C-NMR (DMSO-*d_6_*) δ: 35.60, 44.23, 56.56 (2C), 60.66, 107.28, 107.73 (2C), 125.56, 126.48, 128.72 (2C), 129.07 (2C), 140.16, 144.61, 147.72, 151.96, 153.06 (2C), 156.83, 167.26. MS (ESI): [M+1]^+^ = 406.3. Anal. calcd for C_22_H_23_N_5_O_3_. C, 65.17; H, 5.72; N, 17.27; found: C, 64.98; H, 5.59; N, 17.11.

##### *N*-(3-Phenylpropyl)-7-(3,4,5-trimethoxyphenyl)-[1,2,4]triazolo[1,5-*a*]pyrimidin-2-amine (**3v**)

Following general procedure C, the crude residue was purified by flash chromatography, using ethyl acetate:methanol 9:1 as eluent, to furnish **3v** as a light yellow solid. Yield: 58%, mp 98–100 °C; ^1^H-NMR (DMSO-*d_6_*) δ: 1.86–1.92 (m, 2H), 2.64 (t, J = 7.6 Hz, 2H), 3.25–3.30 (m, 2H), 3.75 (s, 3H), 3.83 (s, 6H), 7.09 (t, J = 8.4 Hz, 1H), 7.12–7.14 (m, 1H), 7.18-7.24 (m, 4H), 7.37 (d, J = 5.2 Hz, 1H), 7.64 (s, 2H), 8.50 (d, J = 5.2 Hz, 1H); ^13^C-NMR (DMSO-*d_6_*) δ: 31.27, 32.99, 42.12, 56.53 (2C), 60.64, 107.20, 107.65 (2C), 125.57, 126.15 (2C), 128.70 (2C), 128.74, 140.39, 142.22, 144.53, 151.89, 153.04 (2C), 156.69, 167.45. MS (ESI): [M+1]^+^ = 419.9. Anal. calcd for C_23_H_25_N_5_O_3_. C, 65.85; H, 6.01; N, 16.70; found: C, 65.72; H, 5.89; N, 16.60. 

### 3.2. Biological Assays and Computational Studies

#### 3.2.1. Cell Cultures and Viability Assay

All the cell lines used in this paper were of human origin and purchased from the American Type Culture Collection (ATCC, Manassas, VA, USA). Colon adenocarcinoma (HT-29), non-small cell lung carcinoma (A549), cervix carcinoma (HeLa) and breast adenocarcinoma (MDA-MB-231) cells were grown in RPMI (HT29) or DMEM (A549, HeLa, MDA-MB-231) medium (Gibco, Milano, Italy). Both media were supplemented with 115 units/mL of penicillin G (Gibco, Milano, Italy), 115 μg/mL of streptomycin (Invitrogen, Milano, Italy) and 10% fetal bovine serum (Invitrogen, Milano, Italy). All cell lines were grown at 37 °C in a humidified 5% CO_2_ atmosphere. The cells were grown in 2 mL of complete medium.

Stock solutions were prepared for each compound by dissolving in DMSO at the final concentration of 10 mM.

The cells were seeded in 96-well plates at the appropriate density for each cell line. The respective cell line densities used for the antiproliferative assay were HeLa and HT29 5000 cells/well, A549 4000 cells/well and MDA-MB-231 7000 cells/well. The total volume of medium was 100 µL. After 24 h, the cells were treated by performing serial 5-fold dilutions of the tested compounds starting from a concentration of 10 μM. All experimental conditions were tested in triplicate for statistical analysis. After 72 h of incubation, 10 μL of 100 μg/mL resazurin solution was added to each well and the plate was re-incubated for 3–4 h. The fluorescence of the wells in each plate was monitored using a Spark 10M spectrophotometer (Tecan Group Ltd., Mannedorf, Switzerland) with a 535 nm excitation wavelength and a 600 nm emission wavelength.

The IC_50_ was defined as the compound concentration required to inhibit cell proliferation by 50%, in comparison with cells treated with the maximum amount of DMSO (0.25%), which was considered 100% viability.

#### 3.2.2. Effects on Tubulin Polymerization and on Colchicine Binding to Tubulin

Bovine brain tubulin was purified as described previously [79]. To evaluate the effects of the compounds on tubulin assembly in vitro [80], varying concentrations were preincubated with 10 μM tubulin in 0.8 M monosodium glutamate (pH 6.6) at 30 °C and the reaction mixtures then cooled to 0 °C. After addition of GTP, the mixtures were transferred to 0 °C cuvettes in Beckman Coulter (Brea, CA, USA) DU-7400/DU-7500 recording spectrophotometers equipped with electronic temperature controllers and warmed to 30 °C, and the assembly of tubulin was observed turbidimetrically. The IC_50_ was defined as the compound concentration that inhibited the extent of assembly by 50% after a 20 min incubation. Inhibition of colchicine binding to tubulin was measured as described before [81], except that the reaction mixtures contained 0.5 μM tubulin and 5 μM each of [^3^H]colchicine and test compound. Only one DEAE-cellulose filter was used per sample, and filtration was by gravity. 

Bovine brain tubulin (see ref. [79] for details of purification) was used to examine effects on tubulin polymerization [80] and colchicine binding [81]. Briefly, in the polymerization experiments, different compound concentrations were preincubated with 10 µM tubulin for 15 min at 30 °C and cooled on ice. After GTP was added to each reaction mixture, the samples were transferred to 0 °C cuvettes in recording spectrophotometers with electronic temperature controllers. The temperature was raised to 30 °C over about 30 s, and turbidity development was followed for 20 min at 350 nm. The IC50 was the compound concentration that inhibited net assembly by 50%. In the current studies, [^3^H]colchicine (from American Radiolabeled Corp., St. Louis, MO) binding was measured after 10 min at 37 °C. Reaction mixtures contained 0.5 µM tubulin and 5.0 µM each of the [^3^H]colchicine and the potential inhibitor, and a single DEAE-cellulose filter was used for each sample, with gravity filtration.

#### 3.2.3. Molecular Modeling

All molecular docking studies were performed on a Viglen Genie Intel^®^CoreTM i7-3770 vPro CPU@ 3.40 GHz x 8 running Ubuntu 18.04. Molecular Operating Environment (MOE) 2022.02 [82] and Maestro (Schrödinger Release 2022-2) [83] were used as molecular modeling software. The tubulin structure was downloaded from the protein data bank, (http://www.rcsb.org/, accessed on 1 July 2022; PDB code 4O2B) and then the dimeric tubulin structure was prepared using the Schrödinger Protein Preparation Wizard by assigning bond orders, adding hydrogens and performing a restrained energy minimization of the added hydrogens using the OPLS_2005 force field. Compounds to be docked were built with MOE and then prepared using the Maestro LigPrep tool by energy minimizing the structures (OPLS_2005 force filed), generating possible ionization states at pH 7 ± 2, tautomers and low-energy ring conformers. A 16 Å docking grid (inner-box 10 Å and outer-box 26 Å) was prepared using as centroid the co-crystallized colchicine. Glide SP precision was adopted for molecular docking studies, using the default parameters, and including 15 output poses per input ligand in the solution. The docking output database was saved as a mol2 file. The docking poses were visually inspected to evaluate their ability to bind in the colchicine binding site. Ligand–protein interactions and ligand–protein clashes were calculated using the MOE contacts tool.

#### 3.2.4. Analysis of Cell Cycle by Flow Cytometry

For these experiments, HeLa cells were used, which were seeded in 6-well plates at a concentration of 5 × 10^5^/well in a final volume of 2 mL culture medium. The cells were then treated with the test compounds for 24 h at the indicated concentrations. After this incubation period, the cells were detached with trypsin-EDTA and harvested by centrifugation. The pellet thus obtained was fixed in 70% ice-cold ethanol.

After this incubation period, the cells were detached with trypsin-EDTA and harvested by centrifugation. The pellet thus obtained was fixed in 70% ice-cold ethanol for at least 1 h. The cells thus fixed were treated with a 0.1% *v*/*v* solution of Triton_X-100 in phosphate buffered saline (PBS) containing RNAseA and propidium iodide (PI) at the final concentration of 0.02 mg/mL. The cells were incubated at room temperature for 30 min and then analyzed on a Cytomic FC500 flow cytometer (Beckman Coulter) in the FL3 channel. DNA histograms were analyzed using MultiCycle for Windows (Phoenix Flow Systems, San Diego, CA, USA).

#### 3.2.5. Apoptosis Assay

The quantification of the apoptosis induced by the test compounds was carried out by flow cytometric analysis using the Annexin-V Fluos kit (Roche Diagnostics, Rotkreuz, Switzerland) following the manufacturer’s instructions. The HeLa cells treated with the test compounds for different incubation times and at the indicated concentrations were then labeled with annexin V/FITC and PI and analyzed with Coulter Cytomics FC500 (Beckman Coulter) in the FL1 and FL3 channel, respectively.

#### 3.2.6. Analysis of Mitochondrial Potential

The analysis of the mitochondrial potential was carried out by flow cytometric analysis. Briefly, the cells treated with the test compound were labeled with the JC-1 dye as previously described [53]. The labeled cells were analyzed using the Coulter Cytomics FC500 (Beckman Coulter) in the FL1 and FL2 channel, respectively.

#### 3.2.7. Western Blot Analysis

Hela cells treated with the test compounds were harvested by centrifugation and then lysed with 0.1% (*v*/*v*) Triton X-100 containing RNase A at 0 °C. The protein content of the solutions was measured and analyzed as described previously [65]. The antibodies directed against cyclin B, cdc2 (Y15), ATR,Bcl-2, cleaved caspase-9 (D330) and actin were purchased from Cell Signaling. The membranes were visualized using ECL select (GE Healthcare, Uppsal, Sweden), and images were acquired using an Uvitec-Alliance imaging system.

#### 3.2.8. In Vivo Experiments on Zebrafish Model

##### Husbandry and Maintenance

Zebrafish (*Danio rerio*) were obtained, staged and raised as described previously [65] and maintained according to the OPBA of the Istituto di Ricerca Pediatrica guidelines. All procedures were conducted following the recommendations and the guidelines of the Animal Use Ethics Committee concerning the protection of animals used for scientific purposes.

##### Drug Toxicity Assessment on Zebrafish Embryos

Wild-type AB zebrafish embryos were treated with chemicals from shield stage (6 hpf) to larval stage (72 hpf) in a 12-well plate, by adding 20 larvae/well for each experimental condition. The zebrafish embryos were treated with compound **3d** at the concentration of 30 nm and 300 nM. These concentrations were selected on the basis of the IC_50_ found above in HeLa cells. The drugs were added directly to the fish water diluted directly from the stock solution in DMSO. Embryos treated with the highest dose of DMSO were used as negative controls and to confirm that this dose of vehicle does not cause any adverse effects towards zebrafish. Subsequently, the treated embryos were kept at a constant temperature (28.5 °C). Embryos are monitored daily through a stereo microscope (Nikon SMZ745T; Nikon, Japan) and the morphological changes as well as the number of dead embryos were evaluated and recorded.

##### Xenograft Model: Injection and Treatment

Prior to microinjections into Tg zebrafish embryos (fli1: EGFP) [84], HeLa tumor cells were labeled with the VybrantTM DiI dye (Invitrogen, ThermoFisher, Carlsbad, CA, USA). The fluorescent cells were loaded into borosilicate glass capillary needles (OD/ID: 1.00/0.75 mm, World Precision Instruments (WPI, Friedberg, Germany) and injected (200 cells), through a pneumatic picopump, into the Cuvier duct of 48 hpf embryos previously anesthetized with tricaine (0.02%, Sigma-Aldrich, Milan, Italy).

After injection, the xenograft-harboring larvae were incubated to recover at 34° C in fish water containing phenylthiourea (PTU) to inhibit the pigmentation process. After 2 h from the injection, the embryos were examined to ensure homogeneity of the xenografts. For drug treatments, only successfully injected xenograft larvae were selected, with approximately 200 HeLa fluorescent cells scattered around the caudal area. 

The injected xenografts were exposed to the doses of **3d** used above with DMSO as control.

For cancer cell imaging and fluorescence quantification, anesthetized embryos were distributed to 96-well plates with one embryo/well. Initially (time 0 h, pre-treatment) and after one-day post-treatment, the tumors were photographed with a Zeiss AxioObserver microscope for live-cell imaging. 

## 4. Conclusions

A series of twenty-two compounds, based on different amines at the 2-position of the 7-(3′,4′,5′-trimethoxyphenyl) [1,2,4]triazolo[1,5-*a*]pyrimidine pharmacophore, was synthesized by a facile and efficient three-step procedure. The modifications were focused at the 2-position of the triazolopyrimidine scaffold by using aromatic amines or arylalkyl amines such as benzylamines, 2-phenylethylamine and 3-phenylpropylamine, with the phenyl ring decorated with electron-releasing or electron-withdrawing groups.

Three of the synthesized aniline derivatives, **3d** (*p*-Me), **3f** (*m*,*p*-diMe) and **3h** (*p*-Et), had the best antiproliferative activities against the HeLa, A549 and HT-29 cell lines. Compounds **3d** and **3f** strongly inhibited tubulin assembly, with inhibitor potency superior to (**3d**) or comparable with (**3f**) that of CA-4. The *p*-toluidino derivative **3d** was the most potent inhibitor of tubulin polymerization and of colchicine binding (IC_50_ = 0.45 μM for assembly, 72% inhibition of the binding of 5 µM colchicine, with tubulin and the inhibitor at 0.5 and 5 μM, respectively), and the antiproliferative activity of this molecule in terms of IC_50_s ranged from 30 to 430 nM in the four tumor cell lines examined, superior to the IC_50_ obtained with CA-4 against the A549 and HeLa lines. 

In comparison with the 1-(3′,4′,5′-trimethoxybenzoyl)-5-amino-1,2,4-triazole **2a,** compound **3d** was almost 2-fold more potent as an inhibitor of tubulin assembly but about 1-2-fold less active as an antiproliferative agent against HeLa, HT-29 and A549 cells, suggesting that the previously published derivative **2a** may exert its potent antiproliferative effect by a mechanism other than inhibition of tubulin polymerization. Alternatively, the reduced potency of **3d** with respect to **2a** on the panel of cancer cell lines can possibly be rationalized by a limited penetration into the cells or any other mechanism limiting the accessibility of this molecule to the cellular tubulin. As a result, the 7-(3′,4′,5′-trimethoxyphenyl)[1,2,4]triazolo[1,5-*a*]pyrimidine nucleus can be regarded as the same pharmacophore skeleton as the 1-(3′,4′,5′-trimethoxybenzoyl)-5-amino-1,2,4-triazole system.

In agreement with this consideration, we found, as expected, that **3d** in vitro led to cell cycle arrest in the G2/M phase. Immunoblot analysis also showed that treatment of HeLa cells induced the activation of ATR signaling, with the consequent increased expression of cyclin B and a reduction in cdc2 phosphorylation. Compound **3d** induced apoptosis associated with the loss of mitochondrial membrane potential. Moreover, we also demonstrated caspase-9 activation and phosphorylation of the anti-apoptotic Bcl-2 protein, two crucial events in the apoptotic cascade induced by antimitotic compounds.

In vivo experiments carried out in the zebrafish model showed that **3d** had significative anticancer activity because it reduced HeLa cell growth in xenografts implanted in zebrafish embryos and, more importantly, this effect occurred at concentrations that did not cause developmental toxicity.

## Figures and Tables

**Table 1:**
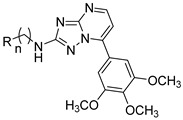
In vitro cell growth inhibitory effects of compounds **3a–v** and CA-4 (**1a**).

Compound	R	n	IC_50_ (μM) ^a^			
			MDA-MB-231	HeLa	A549	HT29
**3a**	C_6_H_5_	0	2.27 ± 0.31	0.80 ± 0.09	1.56 ± 0.06	1.02 ± 0.67
**3b**	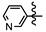	0	>10	>10	>10	3.42 ± 0.41
**3c**	4′-F-C_6_H_4_	0	>10	5.44 ± 1.05	>10	>10
**3d**	4′-CH_3_-C_6_H_4_	0	0.43 ± 0.11	0.038 ± 0.009	0.043 ± 0.024	0.030 ± 0.003
**3e**	3′-CH_3_-C_6_H_4_	0	>10	0.39 ± 0.02	1.87 ± 0.17	1.38 ± 0.09
**3f**	3′,4′-(CH_3_)_2_-C_6_H_3_	0	>10	0.067 ± 0.0023	0.16 ± 0.045	0.16 ± 0.041
**3g**	3′,5′-(CH_3_)_2_-C_6_H_3_	0	>10	>10	>10	>10
**3h**	4′-C_2_H_5_-C_6_H_4_	0	>10	0.24 ± 0.074	0.2 ± 0.044	0.16 ± 0.033
**3i**	4′-*n*-C_3_H_7_-C_6_H_4_	0	>10	>10	>10	>10
**3j**	4′-*i*-C_3_H_7_-C_6_H_4_	0	>10	4.7 ± 0.59	>10	>10
**3k**	4′-OCH_3_-C_6_H_4_	0	5.2 ± 1.8	1.27 ± 0.08	5.32 ± 1.43	2.8 ± 0.20
**3l**	3′-OCH_3_-C_6_H_4_	0	1.76 ± 0.9	0.44 ± 0.08	0.76 ± 0.18	0.76 ± 0.18
**3m**	4′-OC_2_H_5_-C_6_H_4_	0	>10	>10	>10	6.16 ± 0.97
**3n**	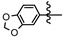	0	6.1 ± 1.72	0.84 ± 0,19	1.44 ± 0.26	1.39 ± 0.06
**3o**	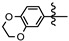	0	>10	0.29 ± 0.031	2.42 ± 0.12	0.67 ± 0.12
**3p**	C_6_H_5_	1	9.3 ± 0.22	>10	>10	>10
**3q**	4′-Cl-C_6_H_4_	1	>10	>10	>10	>10
**3r**	4′-CH_3_-C_6_H_4_	1	>10	>10	>10	>10
**3s**	4′-OCH_3_-C_6_H_4_	1	>10	>10	>10	>10
**3t**	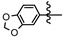	1	>10	>10	>10	>10
**3u**	C_6_H_5_	2	8.7 ± 1.1	>10	>10	6.74 ± 0.50
**3v**	C_6_H_5_	3	>10	>10	>10	>10
CA-4 (**1a**)	-	-	0.005 ± 0.002	0.004 ± 0.001	0.18 ± 0.05	3.10 ± 0.03

^a^ IC_50_ = compound concentration required to inhibit tumor cell proliferation by 50% determined by a resazurin viability assay. Values are the mean ± SE from the dose–response curves of at least three independent experiments carried out in triplicate.

**Table 2:** Inhibition of tubulin polymerization and colchicine binding by compounds **3d**, **3f**, **3h**, **3l** and CA-4 (**1a**).

Compounds	Tubulin Assembly ^a^ IC_50_ ± SD (µM)	Colchicine Binding ^b^ % Inhibition ± SD
**3d**	0.45 ± 0.1	72 ± 5
**3f**	0.80 ± 0.1	18 ± 5
**3h**	1.9 ± 0.2	21 ± 0.9
**3l**	2.2 ± 0.2	39 ± 5
CA-4 (**1a**)	0.75 ± 0.06	98 ± 2

^a^ Inhibition of tubulin polymerization. Tubulin was at 10 μM. ^b^ Inhibition of [^3^H]colchicine binding. Tubulin, [^3^H]colchicine and tested compound were at 0.5, 5 and 5 µM concentrations, respectively.

## Data Availability

Data is contained within the article and Supplementary Material.

## Supplementary Materials

The following supporting information can be downloaded at: https://www.mdpi.com/article/10.3390/ph15081031/s1. ^1^H-NMR and ^13^C-NMR spectra of compounds **3a–v**. Supplementary data associated with this article can be found in the online version.

## References

[1] Akhmanova A., Steinmetz M.O. (2015). Control of microtubule organization and dynamics: Two ends in the limelight. Nat. Rev. Mol. Cell. Biol..

[2] Ludueña R.F., Jeon K.W. (2013). A hypothesis on the origin and evolution of tubulin. International Review of Cell and Molecular Biology.

[3] Knossow M., Campanacci V., Khodja L.A., Gigant B. (2020). The mechanism of tubulin assembly into microtubules: Insights from structural studies. iScience.

[4] Mukhtar E., Adhami V.M., Mukhtar H. (2014). Targeting microtubules by natural agents for cancer therapy. Mol. Cancer Ther..

[5] Karahalil B., Yardım-Akaydin S., Nacak Baytas S. (2019). An overview of microtubule targeting agents for cancer therapy. Arh. Hig. Rada Toksikol..

[6] Čermák V., Dostál V., Jelínek M., Libusová L., Kovář J., Rösel D., Brábek J. (2020). Microtubule-targeting agents and their impact on cancer treatment. Eur. J. Cell Biol..

[7] Field J.J., Kanakkanthara A., Miller J.H. (2014). Microtubule-targeting agents are clinically successful due to both mitotic and interphase impairment of microtubule function. Bioorg. Med. Chem..

[8] Cao Y.N., Zheng L.L., Wang D., Liang X.X., Gao F., Zhou X.L. (2018). Recent advances in microtubule-stabilizing agents. Eur. J. Med. Chem..

[9] Seligmann J., Twelves C. (2013). Tubulin: An example of targeted chemotherapy. Future Med. Chem..

[10] Gigant B., Wang C., Ravelli R.B.G., Roussi F., Steinmetz M.O., Curmi P.A., Sobel A., Knossow M. (2005). Structural basis for the regulation of tubulin by vinblastine. Nature.

[11] Ranaivoson F.M., Gigant B., Berritt S., Joullié M., Knossow M. (2012). Structural plasticity of tubulin assembly probed by vinca-domain ligands. Acta Crystallogr. Sect. D Biol. Crystallogr..

[12] Coderch C., Morreale A., Gago F. (2012). Tubulin-based structure-affinity relationships for antimitotic vinca alkaloids. Anticancer Agents Med. Chem..

[13] Parness J., Horwitz S. (1981). Taxol binds to polymerized tubulin in vitro. J. Cell Biol..

[14] Prota A.E., Bargsten K., Zurwerra D., Field J.J., Diaz J.F., Altmann K.H., Steinmetz M.O. (2013). Molecular mechanism of action of microtubule-stabilizing anticancer agents. Science.

[15] Yang C.H., Horwitz S.B. (2017). Taxol(®): The First Microtubule Stabilizing Agent. Int. J. Mol. Sci..

[16] Katsetos C.D., Dráber P. (2012). Tubulins as therapeutic targets in cancer: From bench to bedside. Curr. Pharm. Des..

[17] Steinmetz M.O., Prota A.E. (2018). Microtubule-targeting agents: Strategies to hijack the cytoskeleton. Trends Cell Biol..

[18] Dumontet C., Jordan M.A. (2010). Microtubule-binding agents: A dynamic field of cancer therapeutics. Nat. Rev. Drug Discov..

[19] Stanton R.A., Gernert K.M., Nettles J.H., Aneja R. (2011). Drugs that target dynamic microtubules: A new molecular perspective. Med. Res. Rev..

[20] Arnst K.E., Banerjee S., Chen H., Deng S., Hwang D.J., Li W., Miller D.D. (2019). Current advances of tubulin inhibitors as dual acting small molecules for cancer therapy. Med. Res. Rev..

[21] Florian S., Mitchison T.J. (2016). Anti-microtubule drugs. Methods Mol. Biol..

[22] Vindya N.G., Sharma N., Yadav M., Ethiraj K.R. (2015). Tubulins-the target for anticancer therapy. Curr. Top. Med. Chem..

[23] van Vuuren R.J., Visagie M.H., Theron A.E., Joubert A.M. (2015). Antimitotic drugs in the treatment of cancer. Cancer Chemother. Pharmacol..

[24] Bukhari S.N.A., Kumar G.B., Revankar H.M., Qin H.-L. (2017). Development of combretastatins as potent tubulin polymerization inhibitors. Bioorg. Chem..

[25] Li W., Sun H., Xu S., Zhu Z., Xu J. (2017). Tubulin inhibitors targeting the colchicine binding site: A perspective of privileged structures. Future Med. Chem..

[26] Lu Y., Chen J.J., Xiao M., Li W., Miller D.D. (2012). An overview of tubulin inhibitors that interact with the colchicine binding site. Pharm. Res..

[27] Kaur R., Kaur G., Gill R.K., Soni R., Bariwal J. (2014). Recent developments in tubulin polymerization inhibitors: An overview. Eur. J. Med. Chem..

[28] Pettit G.R., Cragg G.M., Singh S.B. (1987). Antineoplastic agents, 122. Constituents of Combretum caffrum. J. Nat. Prod..

[29] Lin C.M., Ho H.H., Pettit G.R., Hamel E. (1989). Antimitotic natural products combretastatin A-4 and combretastatin A-2: Studies on the mechanism of their inhibition of the binding of colchicine to tubulin. Biochemistry.

[30] Griggs J., Metcalfe J.C., Hesketh R. (2001). Targeting tumour vasculature: The development of combretastatin A4. Lancet Oncol..

[31] Nagaiah G., Remick S.C. (2010). Combretastatin A4 phosphate: A novel vascular disrupting agent. Future Oncol..

[32] Tewari K.S., Sill M.W., Coleman R.L., Aghajanian C., Mannel R., DiSilvestro P.A., Powell M., Randall L.M., Farley J., Rubin S.C. (2020). Bevacizumab plus fosbretabulin in recurrent ovarian cancer: Overall survival and exploratory analyses of a randomized phase II NRG oncology/gynecologic oncology group study. Gynecol. Oncol..

[33] Chauhan A., Arnold S., Slone S.A., Flynn H., Weiss H., Anthony L.B. (2018). A phase I/II study of fosbretabulin in combination with everolimus in neuroendocrine tumors that have progressed after at least one prior regimen for metastatic disease. J. Clin. Oncol..

[34] Patil P.O., Patil A.G., Rane R.A., Patil P.C., Deshmukh P.K., Bari S.B., Patil D.A., Naphade S.S. (2015). Recent advancement in discovery and development of natural product combretastatin-inspired anticancer agents. Anticancer Agents Med. Chem..

[35] Greene L.M., Meegan M.J., Zisterer D.M. (2015). Combretastatins: More than just vascular targeting agents?. J. Pharmacol. Exp. Ther..

[36] Mikstacka R., Stefański T., Różański J. (2013). Tubulin-interactive stilbene derivatives as anticancer agents. Cell Mol. Biol. Lett..

[37] Niu L., Yang J., Yan W., Yu Y., Zheng Y., Ye H., Chen X.Q., Chen L. (2019). Reversible binding of the anticancer drug KXO1(tirbanibulin) to the colchicine-binding site of tubulin explains KXO1’s low clinical toxicity. J. Biol. Chem..

[38] Amirall Almirall Announces FDA Approval of Klisyri® (Tirbanibulin), A New Innovative Topical Treatment for Actinic Keratosis. https://www.almirall.com/newsroom/news/almirall-announces-fda-approval-of-klisyri®-tirbanibulin-a-new-innovative-topical-treatment-for-actinic-keratosis.

[39] Mosca L., Ilari A., Fazi F., Assaraf Y.G., Colotti G. (2021). Taxanes in cancer treatment: Activity, chemoresistance and its overcoming. Drug Resist. Updates.

[40] Martino E., Casamassima G., Castiglione S., Cellupica E., Pantalone S., Papagni F., Rui M., Siciliano A.M., Collina S. (2018). Vinca alkaloids and analogues as anti-cancer agents: Looking back, peering ahead. Bioorg. Med. Chem. Lett..

[41] Kavallaris M., Tait A.S., Walsh B.J., He L.F., Horwitz S.B., Norris M.D., Haber M. (2001). Multiple microtubule alterations are associated with Vinca alkaloid resistance in human leukemia cells. Cancer Res..

[42] Verrills N.M., Kavallaris M. (2005). Improving the targeting of tubulin-binding agents: Lessons from drug resistance studies. Curr. Pharm. Des..

[43] Fojo A.T., Menefee M. (2005). Microtubule targeting agents: Basic mechanisms of multidrug resistance (MDR). Semin. Oncol..

[44] Maloney S.M., Hoover C.A., Morejon-Lasso L.V., Prosperi J.R. (2020). Mechanisms of taxane resistance. Cancers.

[45] Kavallaris M. (2010). Microtubules and resistance to tubulin-binding agents. Nat. Rev. Cancer.

[46] Stengel C., Newman S.P., Leese M.P., Potter B.V., Reed M.J., Purohit A. (2010). Class III beta-tubulin expression and in vitro resistance to microtubule targeting agents. Br. J. Cancer.

[47] Sève P., Dumontet C. (2008). Is class III β-tubulin a predictive factor in patients receiving tubulin-binding agents?. Lancet Oncol..

[48] Haider K., Rahaman S., Yar M.S., Kamal A. (2019). Tubulin inhibitors as novel anticancer agents: An overview on patents (2013–2018). Expert Opin. Ther. Pat..

[49] Bates D., Eastman A. (2017). Microtubule destabilising agents: Far more than just antimitotic anticancer drugs. Br. J. Clin. Pharmacol..

[50] McLoughlin E.C., O’Boyle N.M. (2020). Colchicine-binding site inhibitors from chemistry to clinic: A review. Pharmaceuticals.

[51] Wang Z., Chen J., Wang J., Ahn S., Li C.M., Lu Y., Loveless V.S., Dalton J.T., Miller D.D., Li W. (2012). Novel tubulin polymerization inhibitors overcome multidrug resistance and reduce melanoma lung metastasis. Pharm. Res..

[52] Nainwal L.M., Alam M.M., Shaquiquzzaman M., Marella A., Kamal A. (2019). Combretastatin-based compounds with therapeutic characteristics: A patent review. Expert Opin. Ther. Pat..

[53] Romagnoli R., Baraldi P.G., Kimatrai Salvador M., Prencipe F., Bertolasi V., Cancellieri M., Brancale A., Hamel E., Castagliuolo I., Consolaro F. (2014). Synthesis, antimitotic and antivascular activity of 1-(3’,4’,5’-trimethoxybenzoyl)-3-arylamino-5-amino-1,2,4-triazoles. J. Med. Chem..

[54] Sun H., Tawa G., Wallqvist A. (2012). Classification of scaffold-hopping approaches. Drug Discov. Today.

[55] Lazzara P.R., Moore T.W. (2020). Scaffold-hopping as a strategy to address metabolic liabilities of aromatic compounds. RSC Med. Chem..

[56] Li C.M., Lu Y., Narayanan R., Miller D.D., Dalton J.T. (2010). Drug metabolism and pharmacokinetics of 4-substituted methoxybenzoyl-aryl-thiazoles. Drug Metab. Dispos..

[57] Lu Y., Chen J., Wang J., Li C.M., Ahn S., Barrett C.M., Dalton J.T., Li W., Miller D.D. (2014). Design, synthesis, and biological evaluation of stable colchicine binding site tubulin inhibitors as potential anticancer agents. J. Med. Chem..

[58] Hwang D.-J., Wang J., Li W., Miller D.D. (2015). Structural optimization of indole derivatives acting at colchicine binding site as potential anticancer agents. ACS Med. Chem. Lett..

[59] Ren Y., Wang Y., Li G., Zhang Z., Ma L., Cheng B., Chen J. (2021). Discovery of novel benzimidazole and indazole analogues as tubulin polymerization inhibitors with potent anticancer activities. J. Med. Chem..

[60] Chen H., Deng S., Albadari N., Yun M.-K., Zhang S., Li Y., Ma D., Parke D.N., Yang L., Seagroves T.N. (2021). Design, synthesis, and biological evaluation of stable colchicine-binding site tubulin inhibitors 6-aryl-2-benzoyl-pyridines as potential anticancer agents. J. Med. Chem..

[61] Zhang N., Ayral-Kaloustian S., Nguyen T., Afragola J., Hernandez R., Lucas J., Gibbons J., Beyer C. (2007). Synthesis and SAR of [1,2,4]triazolo[1,5-*a*]pyrimidines, a class of anticancer agents with a unique mechanism of tubulin inhibition. J. Med. Chem..

[62] Yang F., Yu L.Z., Diao P.C., Jian X.E., Zhou M.F., Jiang C.S., You W.W., Ma V., Zhao P.L. (2019). Novel[1,2,4]triazolo[1,5-*a*]pyrimidine derivatives as potent antitubulin agents: Design, multicomponent synthesis and antiproliferative activities. Bioorg. Chem..

[63] Huo X.-S., Jian X.-E., Ou-Yang J., Chen L., Yang F., Lv D.-X., You W.-W., Rao J.-J., Zhao P.-L. (2021). Discovery of highly potent tubulin polymerization inhibitors: Design, synthesis, and structure-activity relationships of novel 2,7-diaryl-[1,2,4]triazolo[1,5-*a*]pyrimidines. Eur. J. Med. Chem..

[64] Mohamed H.S., Amin N.H., El-Saadi M.T., Abdel-Rahman H.M. (2022). Design, synthesis, biological assessment, and in-silico studies of 1,2,4-triazolo[1,5-*a*]pyrimidine derivatives as tubulin polymerization inhibitors. Bioorg. Chem..

[65] Oliva P., Romagnoli R., Cacciari B., Manfredini S., Padroni C., Brancale A., Ferla S., Hamel E., Corallo D., Aveic S. (2022). Synthesis and biological evaluation of highly active 7-anilino triazolopyrimidines as potent antimicrotubule agents. Pharmaceutics.

[66] Kamal A., Dastagiri D., Ramaiah M.J., Reddy J.S., Bharathi E.V., Reddy M.K., Sagar M.V.P., Reddy T.L., Pushpavalli S.N.C.V.L., Pal-Bhadra M. (2011). Synthesis and apoptosis inducing ability of new anilino substituted pyrimidine sulfonamides as potential anticancer agents. Eur. J. Med. Chem..

[67] Donzelli M., Draetta G.F. (2003). Regulating mammalian checkpoints through cdc25 inactivation. EMBO Rep..

[68] Liu K., Zheng M., Lu R., Du J., Zhao Q., Li Z., Li Y., Zhang S. (2020). The role of CDC25C in cell cycle regulation and clinical cancer therapy: A systematic review. Cancer Cell Int..

[69] Mollinedo F., Gajate C. (2003). Microtubules, microtubule-interfering agents and apoptosis. Apoptosis.

[70] Rovini A., Savry A., Braguer D., Carré M. (2011). Microtubule-targeted agents: When mitochondria become essential to chemotherapy. Biochim. Biophys. Acta-Bioenerg..

[71] Romagnoli R., Baraldi P.G., Lopez Cara C., Hamel E., Basso G., Bortolozzi R., Viola G. (2010). Synthesis and biological evaluation of 2-(3’,4’,5’-trimethoxybenzoyl)-3-aryl/arylaminobenzo[*b*]thiophene derivatives as a novel class of antiproliferative agents. Eur. J. Med. Chem..

[72] Romagnoli R., Baraldi P.G., Kimatrai Salvador M., Preti D., Tabrizi M.A., Brancale A., Fu X.-H., Li J., Zhang S.-Z., Hamel E. (2012). Discovery and optimization of a series of 2-aryl-4-amino-5-(3’,4’,5’-trimethoxybenzoyl)thiazoles as novel anticancer agents. J. Med. Chem..

[73] Romagnoli R., Baraldi P.G., Lopez-Cara C., Preti D., Aghazadeh Tabrizi M., Balzarini J., Bassetto M., Brancale A., Fu X.-H., Gao Y. (2013). Concise synthesis and biological evaluation of 2-aroyl-5-amino benzo[*b*]thiophene derivatives as a novel class of potent antimitotic agents. J. Med. Chem..

[74] Romagnoli R., Baraldi P.G., Lopez-Cara C., Kimatrai Salvador M., Preti D., Aghazadeh Tabrizi M., Balzarini J., Nussbaumer P., Brancale A., Fu X.-H. (2014). Design, synthesis and biological evaluation of 3,5-disubstituted 2-amino thiophene derivatives as a novel class of antitumor agents. Bioorg. Med. Chem..

[75] Singh R., Letai A., Sarosiek K. (2019). Regulation of apoptosis in health and disease: The balancing act of BCL-2 family proteins. Nat. Rev. Mol. Cell Biol..

[76] Eichhorn J.M., Sakurikar N., Alford S.E., Chu R., Chambers T.C. (2013). Critical role of anti-apoptotic Bcl-2 protein phosphorylation in mitotic death. Cell Death Dis..

[77] Whitaker R.H., Placzek W.J. (2019). Regulating the BCL2 family to improve sensitivity to microtubule targeting agents. Cells.

[78] Oliva P., Onnis V., Balboni E., Hamel E., Estévez-Sarmiento F., Quintana J., Estévez F., Brancale A., Ferla S., Manfredini S. (2020). Synthesis and biological evaluation of 2-substituted benzyl/phenylethylamino-4-amino-5-aroyl thiazoles as apoptosis inducing anticancer agents. Molecules.

[79] Hamel E., Lin C.M. (1984). Separation of active tubulin and microtubule-associated proteins by ultracentrifugation and isolation of a component causing the formation of microtubule bundles. Biochemistry.

[80] Hamel E. (2003). Evaluation of antimitotic agents by quantitative comparisons of their effects on the polymerization of purified tubulin. Cell Biochem. Biophys..

[81] Verdier-Pinard P., Lai J.-Y., Yoo H.-D., Yu J., Marquez B., Nagle D.G., Nambu M., White J.D., Falck J.R., Gerwick W.H. (1998). Structure-activity analysis of the interaction of curacin A, the potent colchicine site antimitotic agent, with tubulin and effects of analogs on the growth of MCF-7 breast cancer cells. Mol. Pharmacol..

[82] Molecular Operating Environment (MOE), 2022.02. Chemical Computing Group ULC: Montreal, QC, Canada. http://www.chemcomp.com.

[83] (2022).

[84] Lawson N.D., Weinstein B.M. (2002). In vivo imaging of embryonic vascular development using transgenic zebrafish. Dev. Biol..

