# Supplementary material for: Design, Synthesis and Biological Investigation of 2-Anilino Triazolopyrimidines as Tubulin Polymerization Inhibitors with Anticancer Activities
Source: Pharmaceuticals (Basel). 2022 Aug 21;15(8):1031. doi: 10.3390/ph15081031 (PMC9415608; doi:10.3390/ph15081031)
Supplement: Supplementary file 1 [file pharmaceuticals-15-01031-s001.zip › pharmaceuticals-1863675-supplementary.pdf]

pharmaceuticals

MDPI

Supplementary data: Design, Synthesis and Biological Investigation of 2-Anilino Triazolopyrimidines as Tubulin Polymerization Inhibitors with Anticancer Activities

Romeo Romagnoli, Paola Oliva, Filippo Prencipe, Stefano Manfredini, Federica Budassi, Andrea Brancale, Salvatore Ferla, Ernest Hamel, Diana Corallo, Sanja Aveic, Lorenzo Manfreda, Elena Mariotto, Roberta Bortolozzi and Giampietro Viola

^{1}H-NMR and ^{13}C-NMR spectra of compound **3a** S3

^{1}H-NMR and ^{13}C-NMR spectra of compound **3b** S4

^{1}H-NMR and ^{13}C-NMR spectra of compound **3c** S5

^{1}H-NMR and ^{13}C-NMR spectra of compound **3d** S6

^{1}H-NMR and ^{13}C-NMR spectra of compound **3e** S7

^{1}H-NMR and ^{13}C-NMR spectra of compound **3f** S8

^{1}H-NMR and ^{13}C-NMR spectra of compound **3g** S9

^{1}H-NMR and ^{13}C-NMR spectra of compound **3h** S10

^{1}H-NMR and ^{13}C-NMR spectra of compound **3i** S11

^{1}H-NMR and ^{13}C-NMR spectra of compound **3j** S12

^{1}H-NMR and ^{13}C-NMR spectra of compound **3k** S13

^{1}H-NMR and ^{13}C-NMR spectra of compound **3l** S14

^{1}H-NMR and ^{13}C-NMR spectra of compound **3m** S15

^{1}H-NMR and ^{13}C-NMR spectra of compound **3n** S16

^{1}H-NMR and ^{13}C-NMR spectra of compound **3o** S17

^{1}H-NMR and ^{13}C-NMR spectra of compound **3p** S18

^{1}H-NMR and ^{13}C-NMR spectra of compound **3q** S19

^{1}H-NMR and ^{13}C-NMR spectra of compound **3r** S20

^{1}H-NMR and ^{13}C-NMR spectra of compound **3s** S21

^{1}H-NMR and ^{13}C-NMR spectra of compound **3t** S22

^{1}H-NMR and ^{13}C-NMR spectra of compound **3u** S23

^{1}H-NMR and ^{13}C-NMR spectra of compound **3v** S24

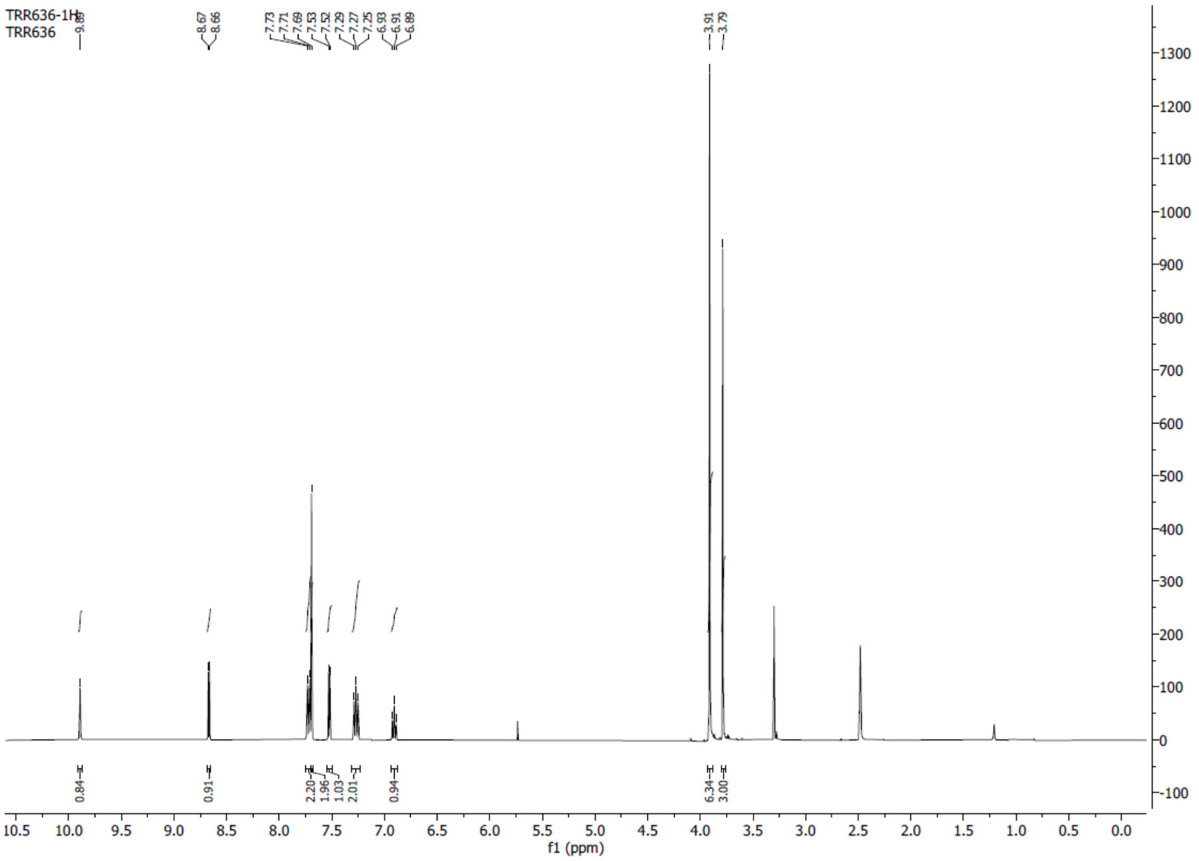

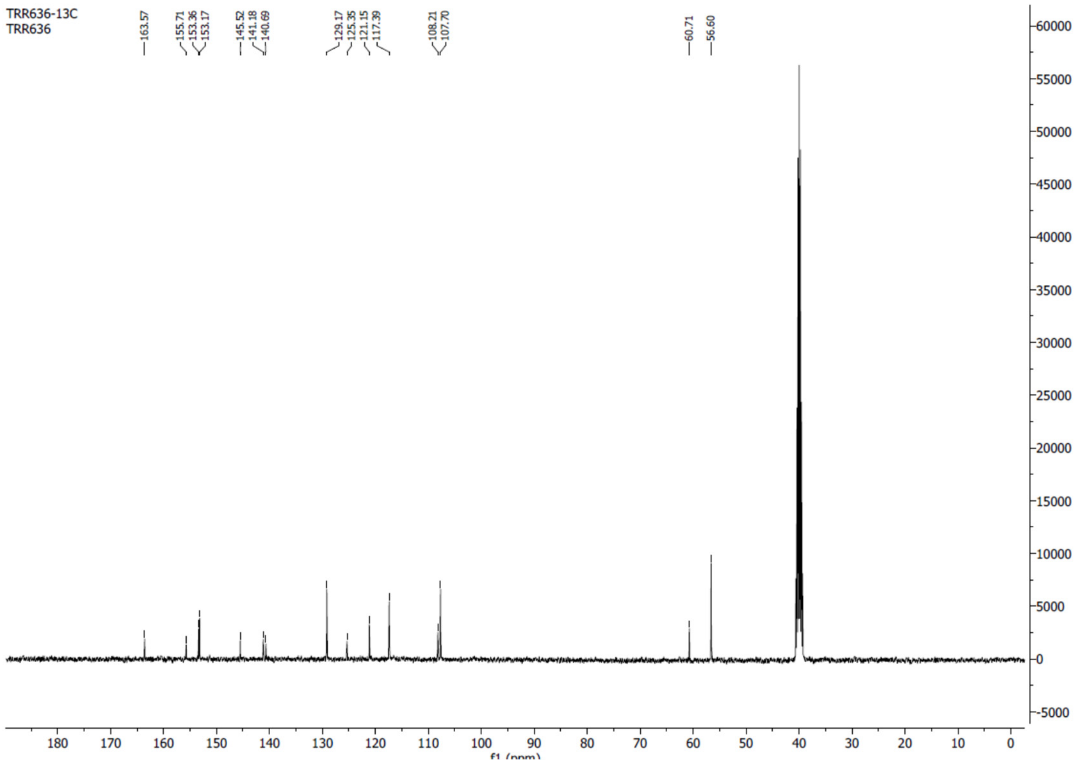

^{1}H-NMR and ^{13}C-NMR spectra of compound **3a**

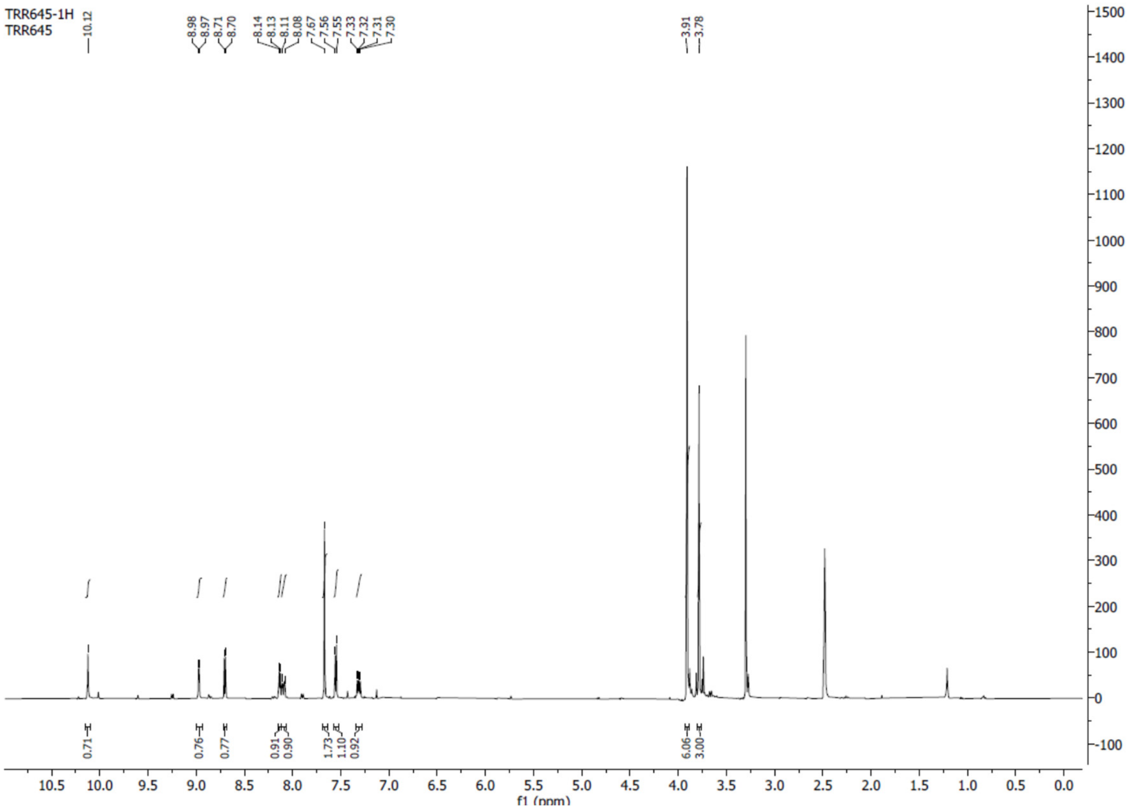

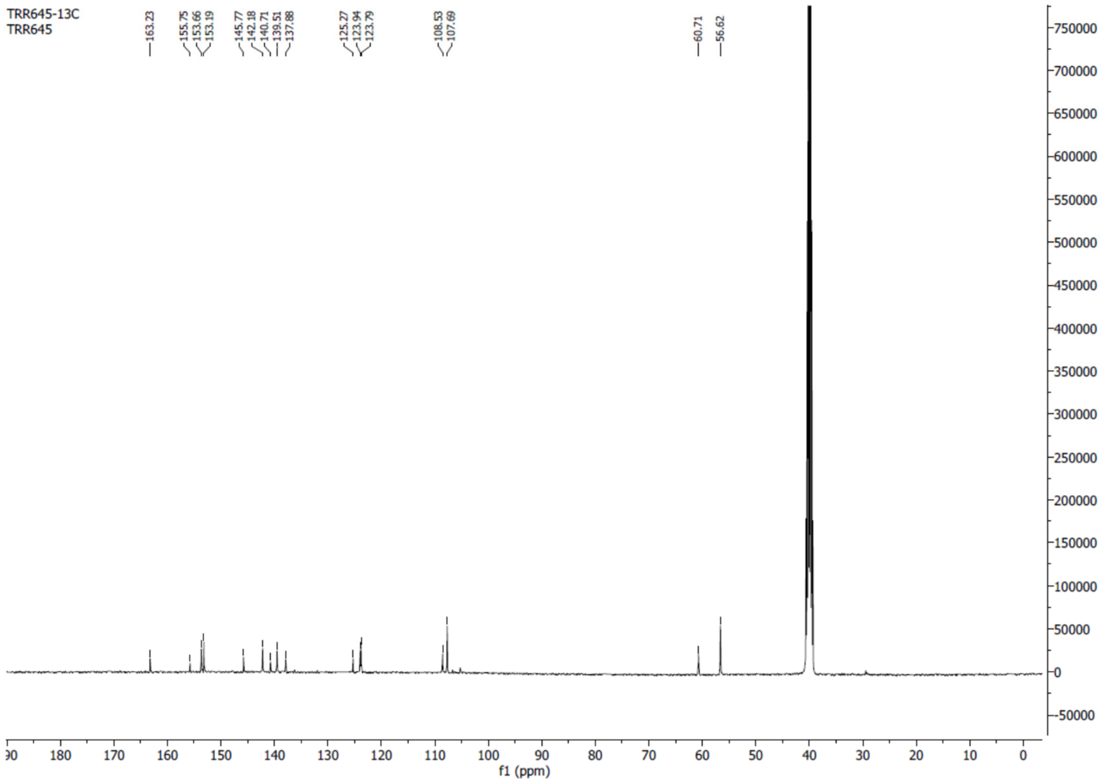

^{1}H-NMR and ^{13}C-NMR spectra of compound **3b**

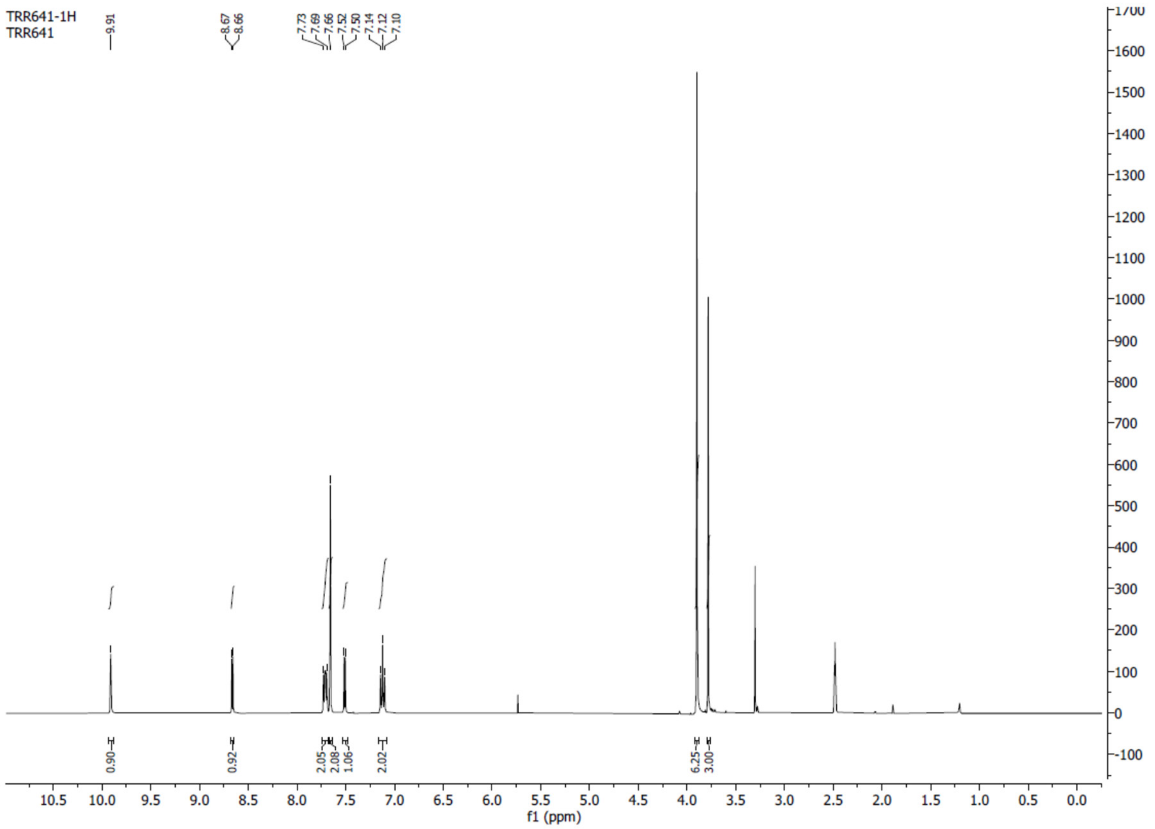

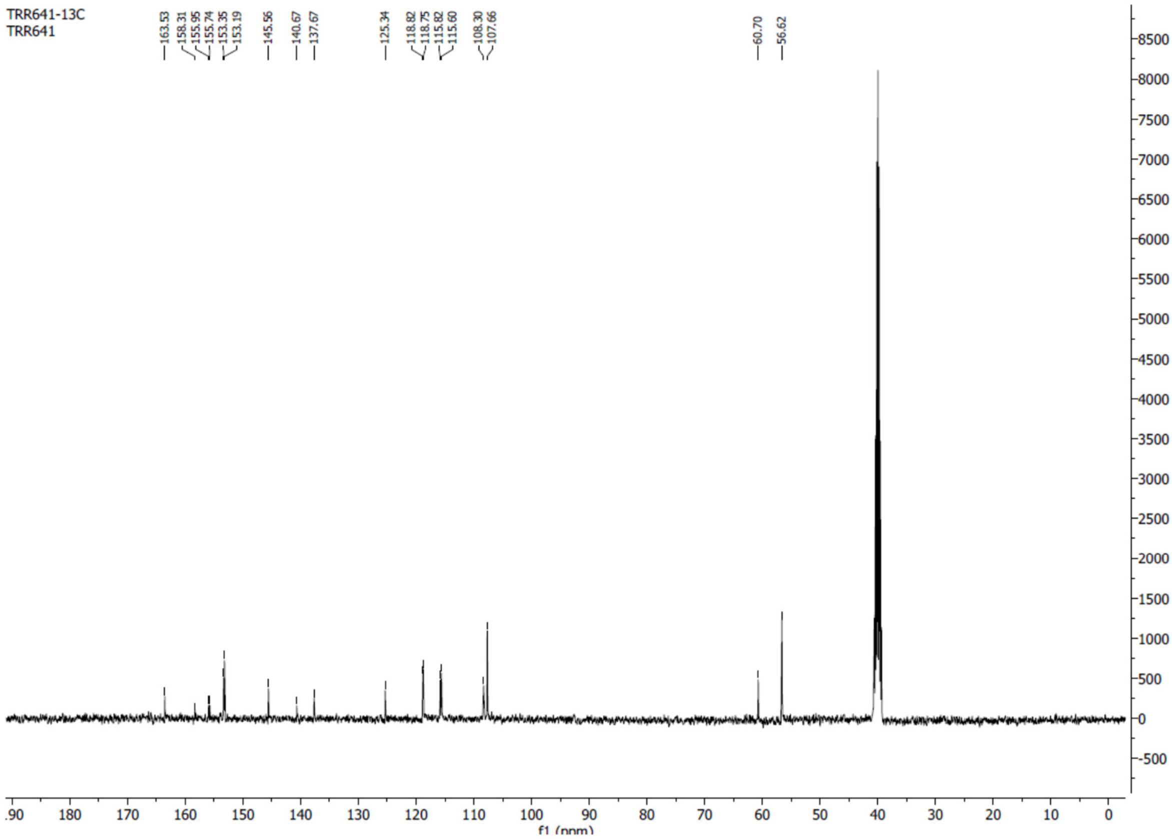

^{1}H-NMR and ^{13}C-NMR spectra of compound **3c**

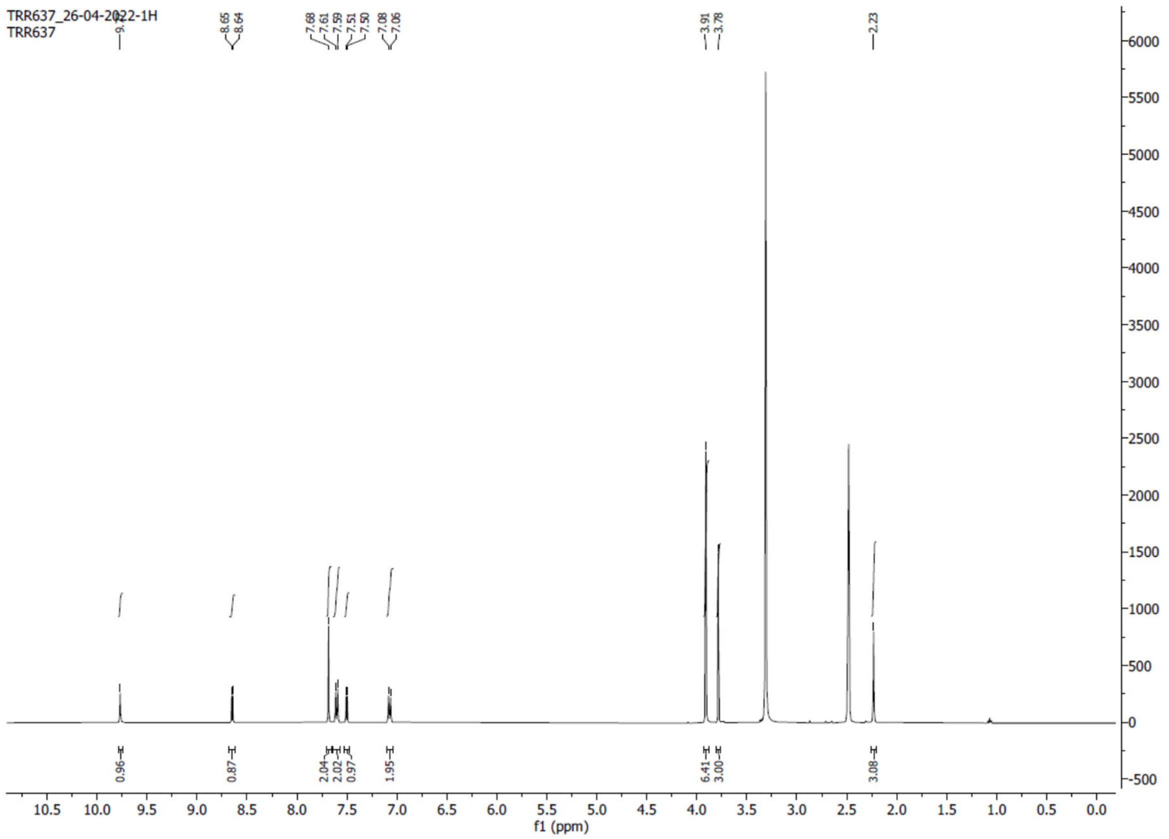

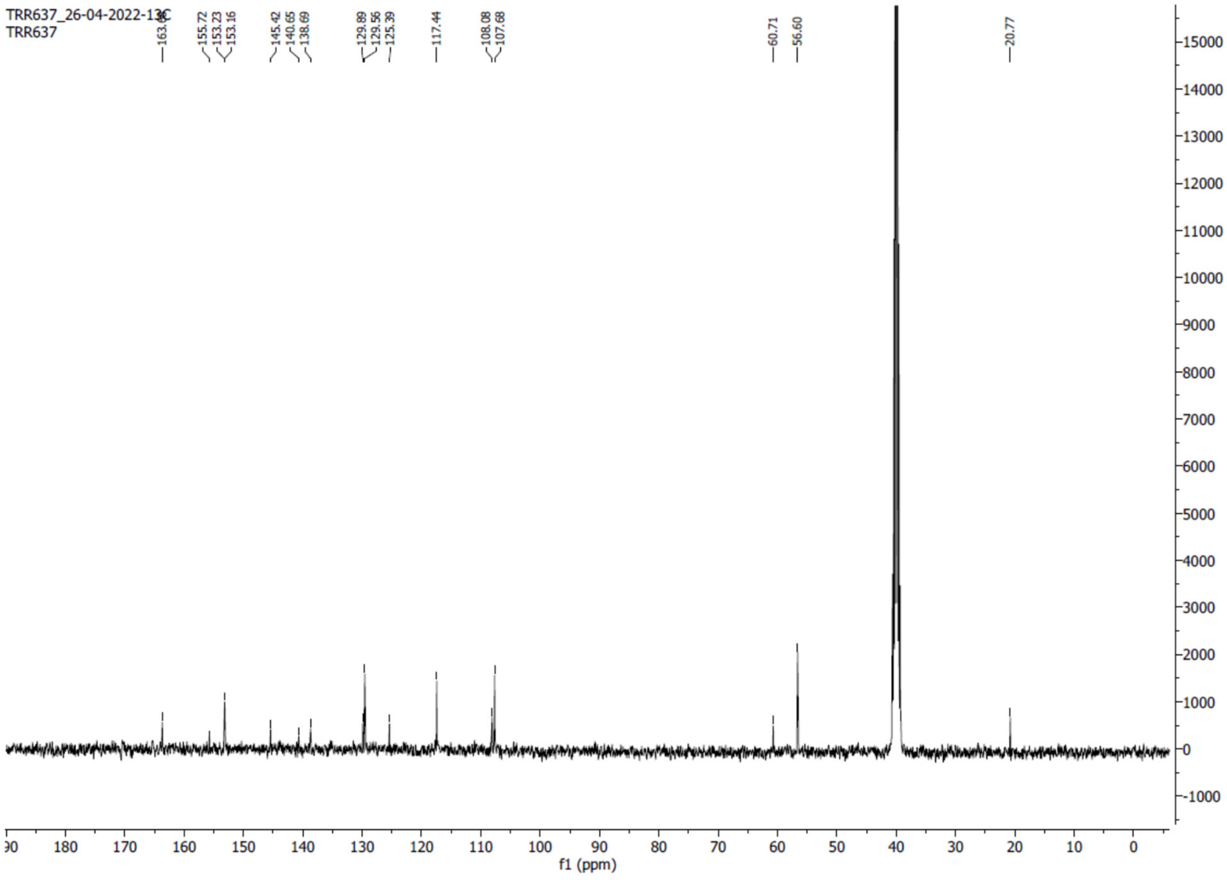

^{1}H-NMR and ^{13}C-NMR spectra of compound **3d**

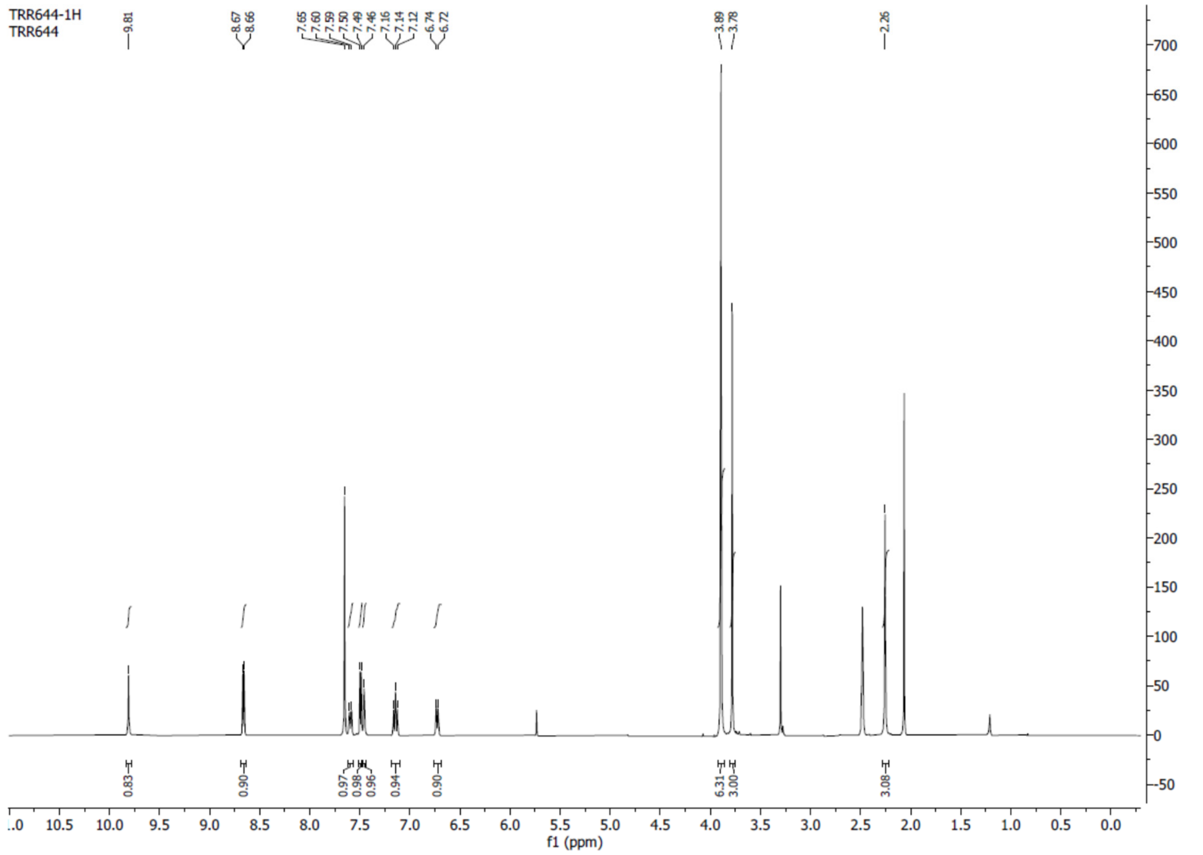

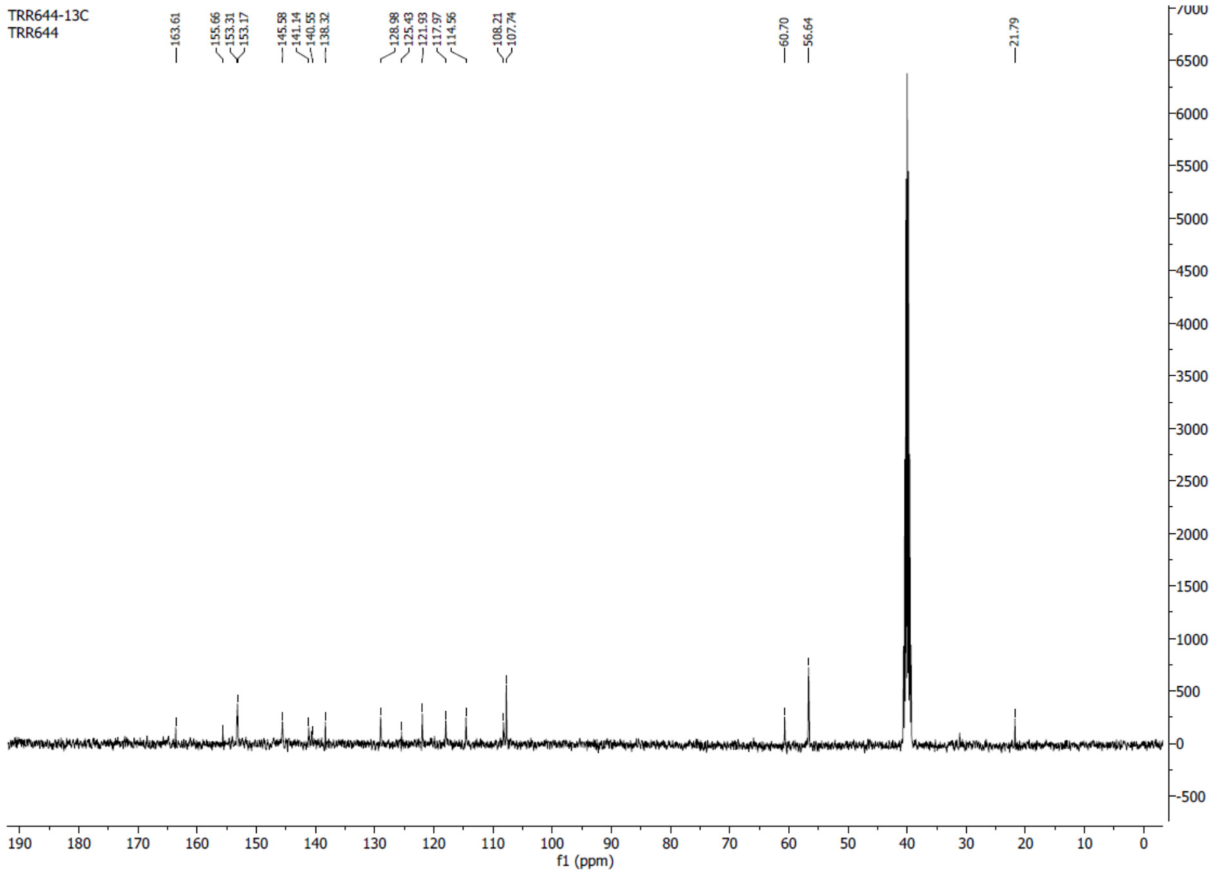

^{1}H-NMR and ^{13}C-NMR spectra of compound **3e**

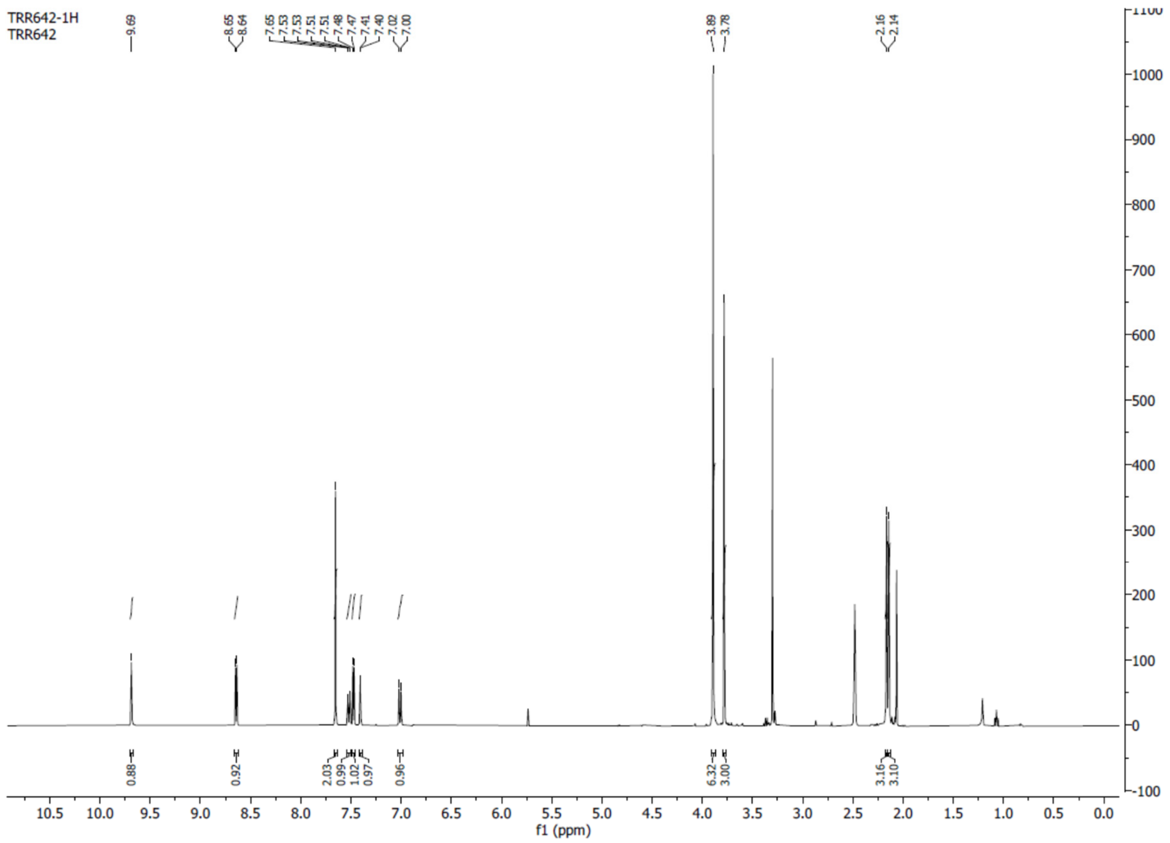

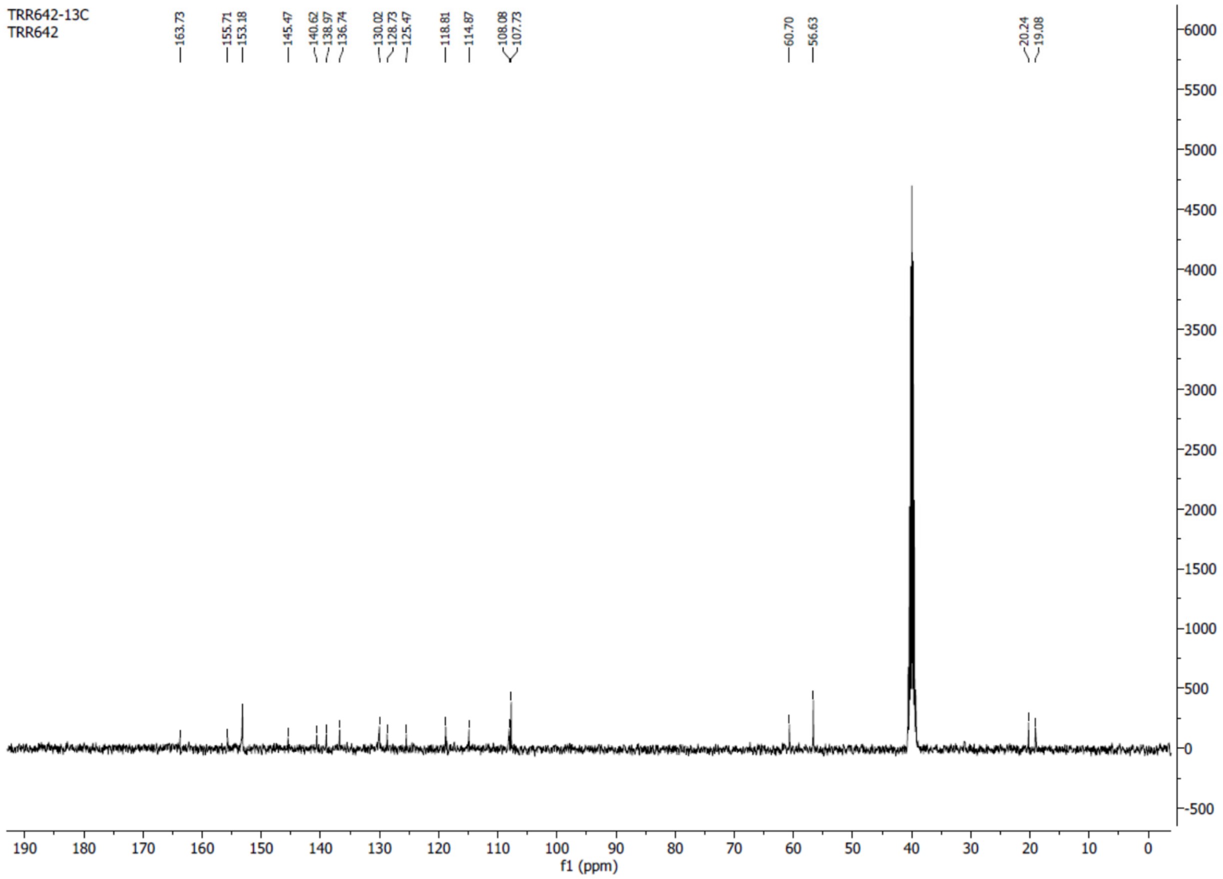

^{1}H-NMR and ^{13}C-NMR spectra of compound **3f**

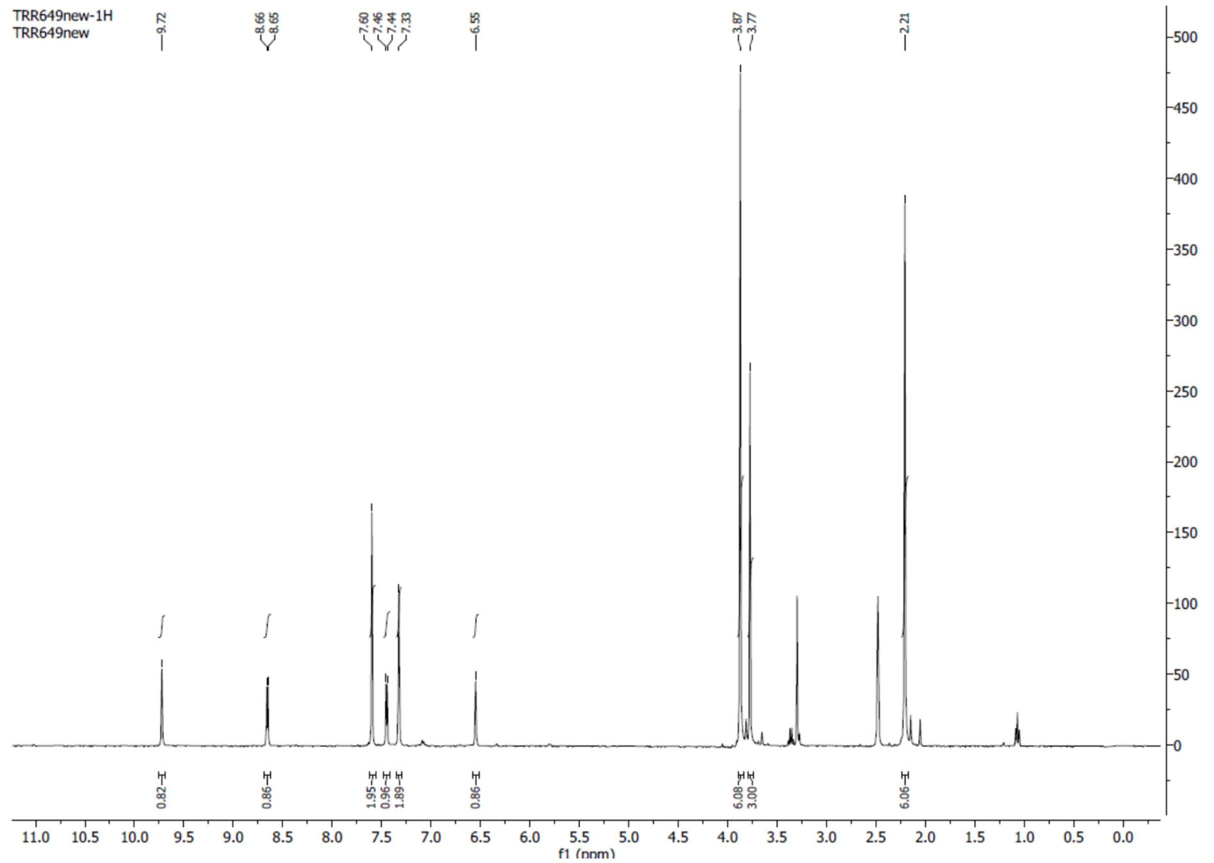

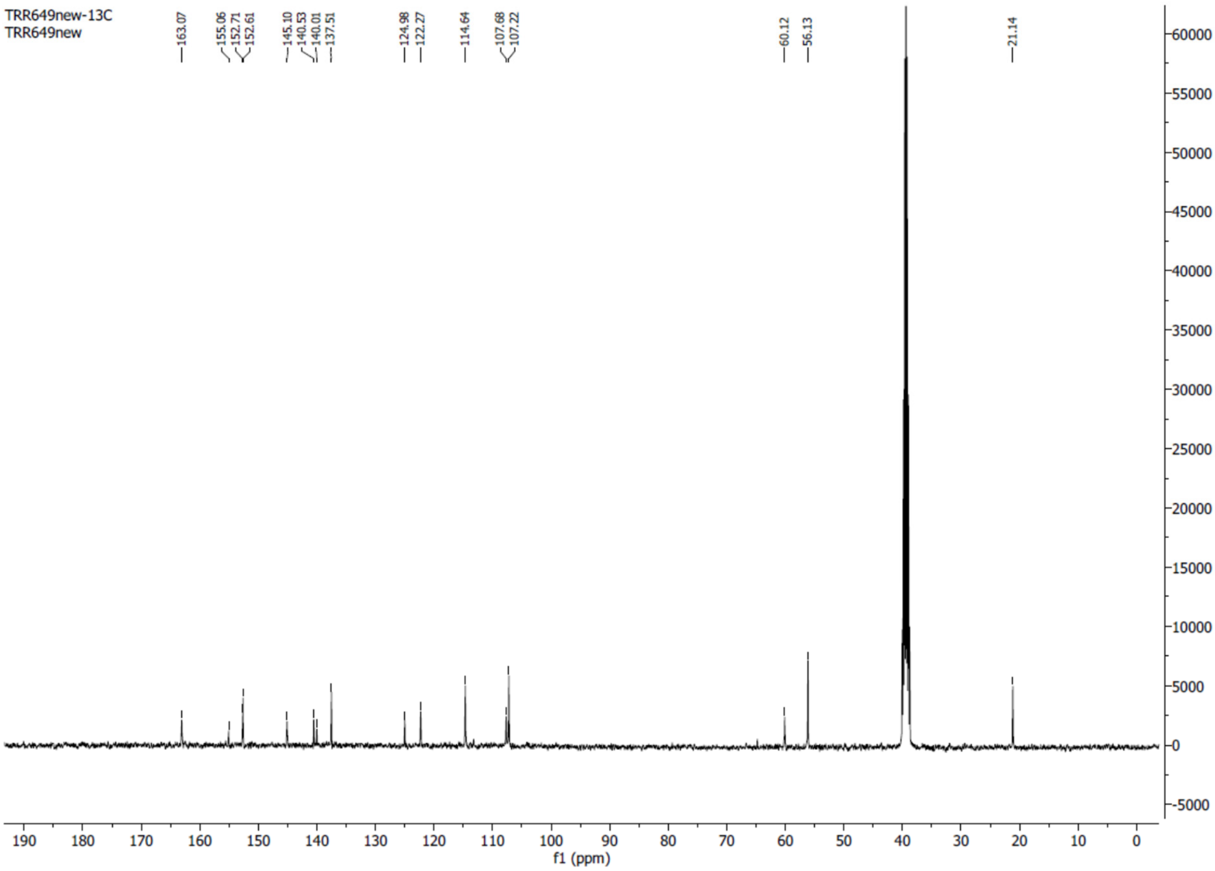

^{1}H-NMR and ^{13}C-NMR spectra of compound **3g**

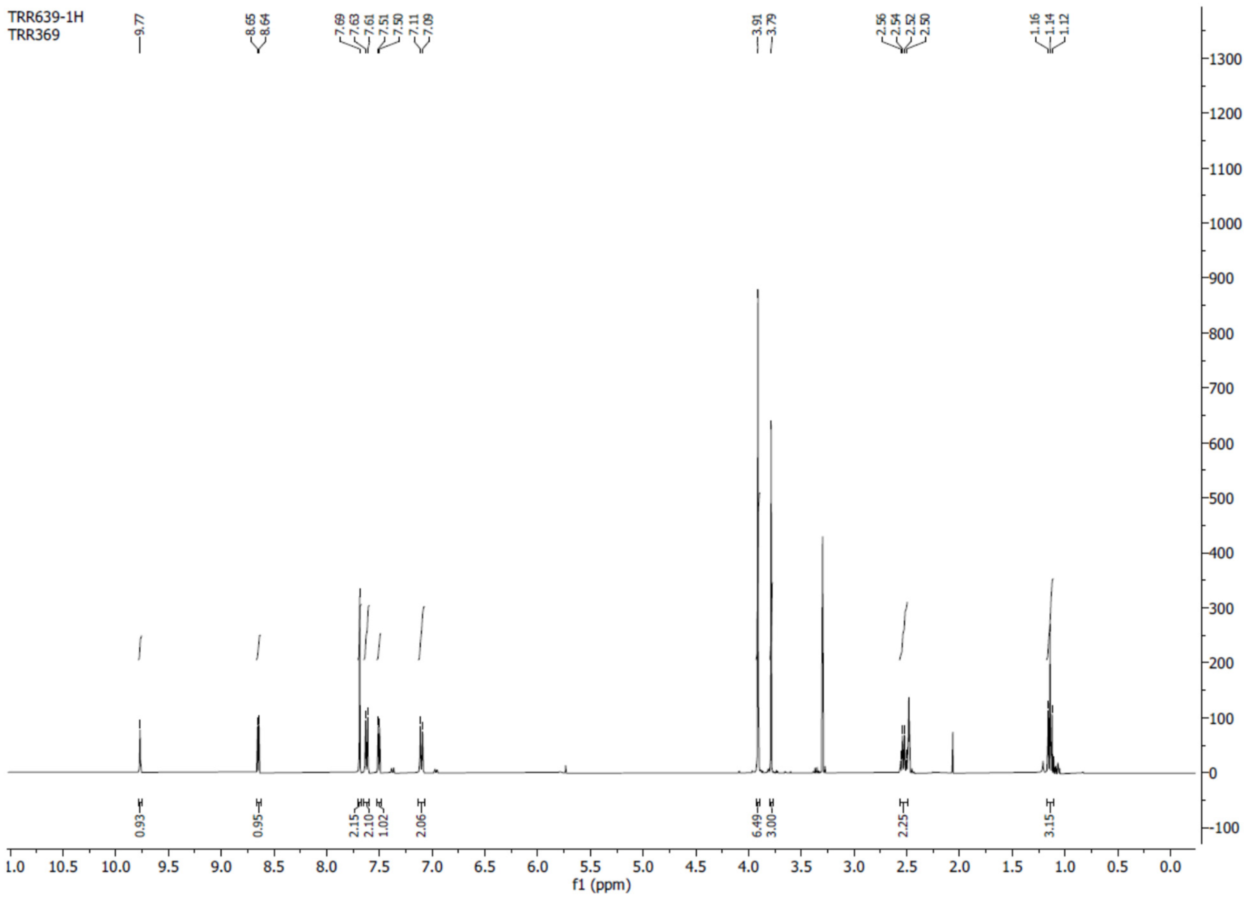

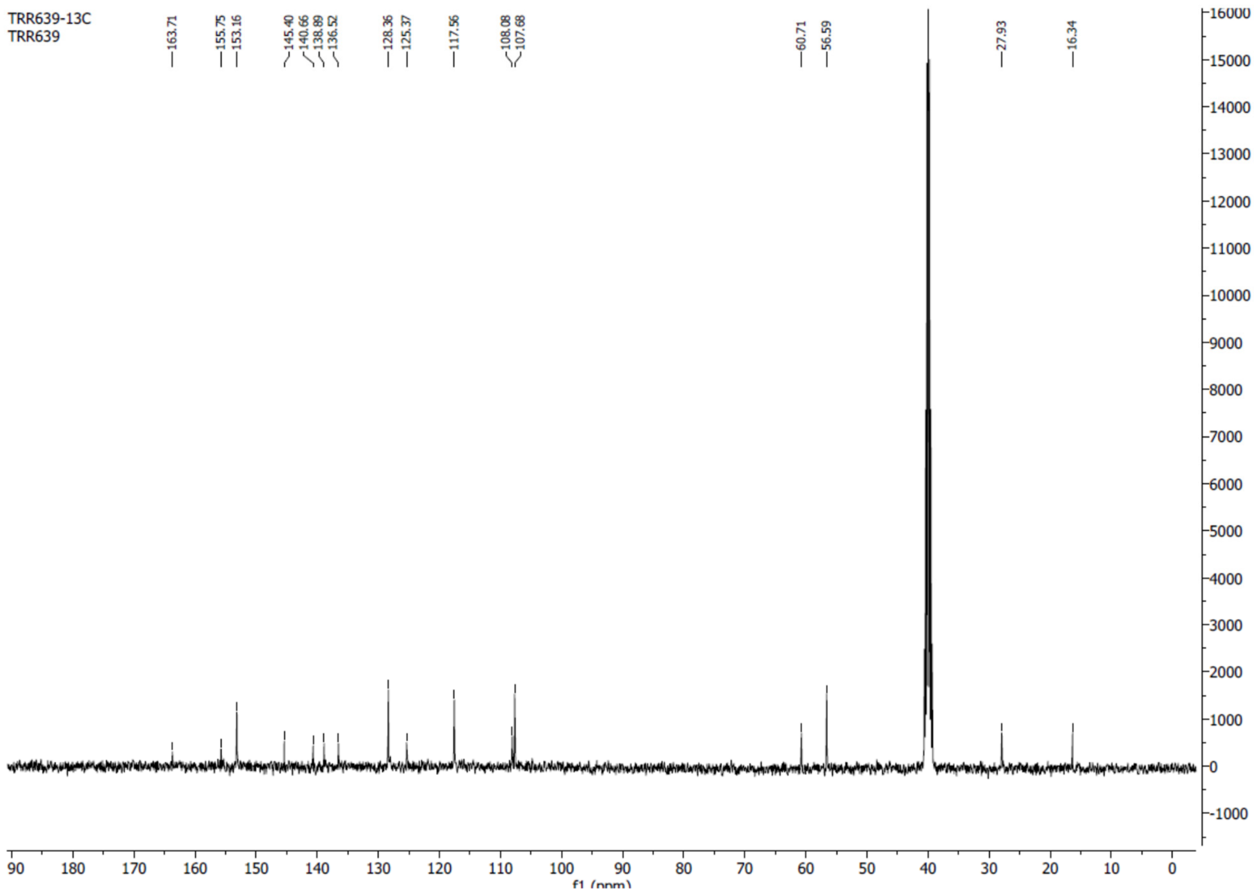

^{1}H-NMR and ^{13}C-NMR spectra of compound **3h**

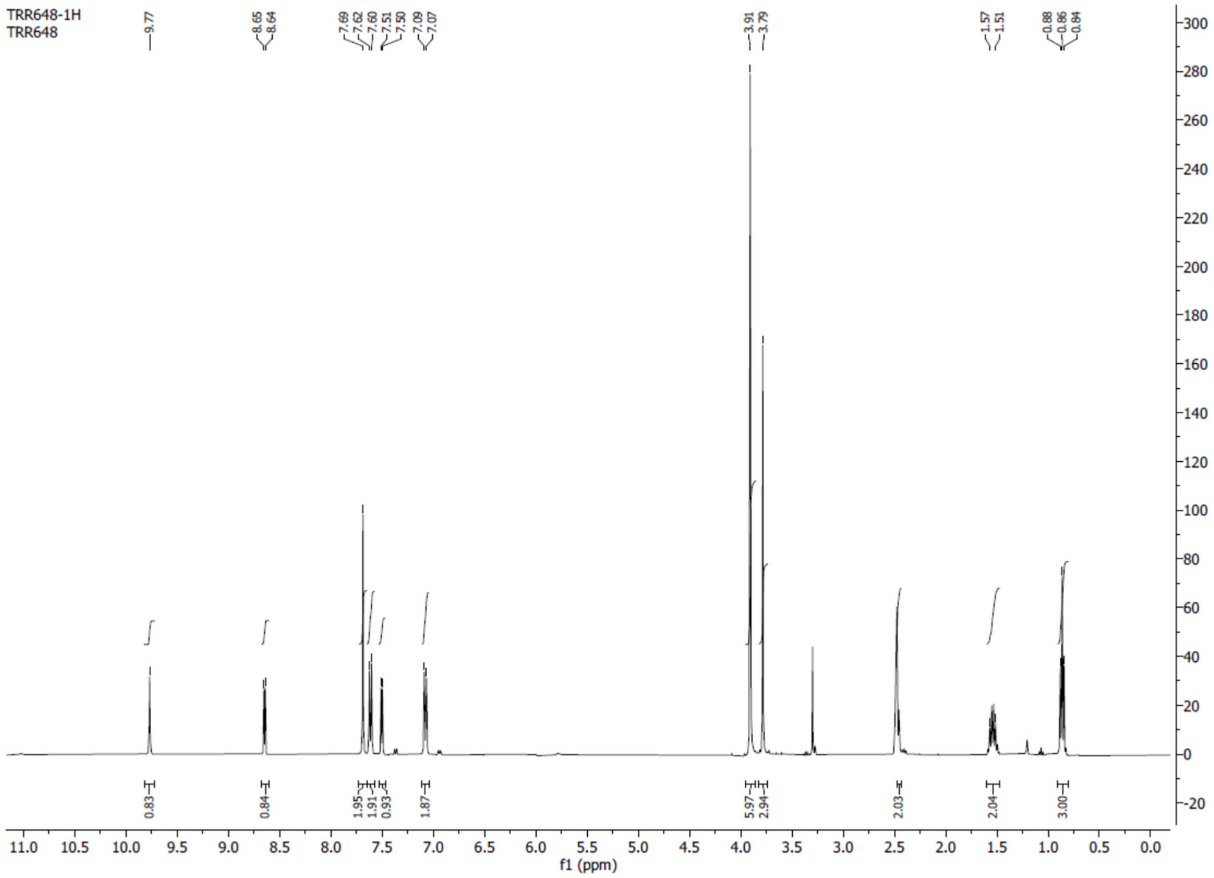

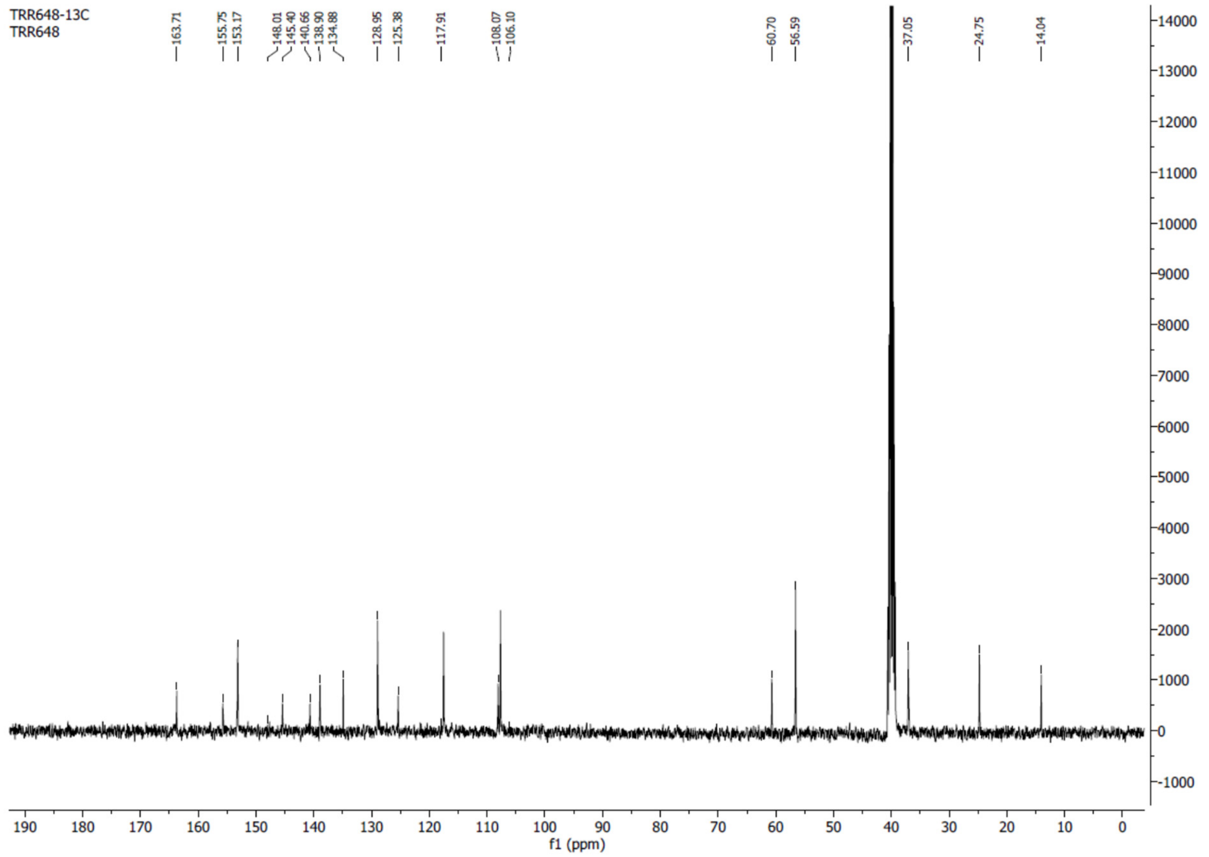

^{1}H-NMR and ^{13}C-NMR spectra of compound **3i**

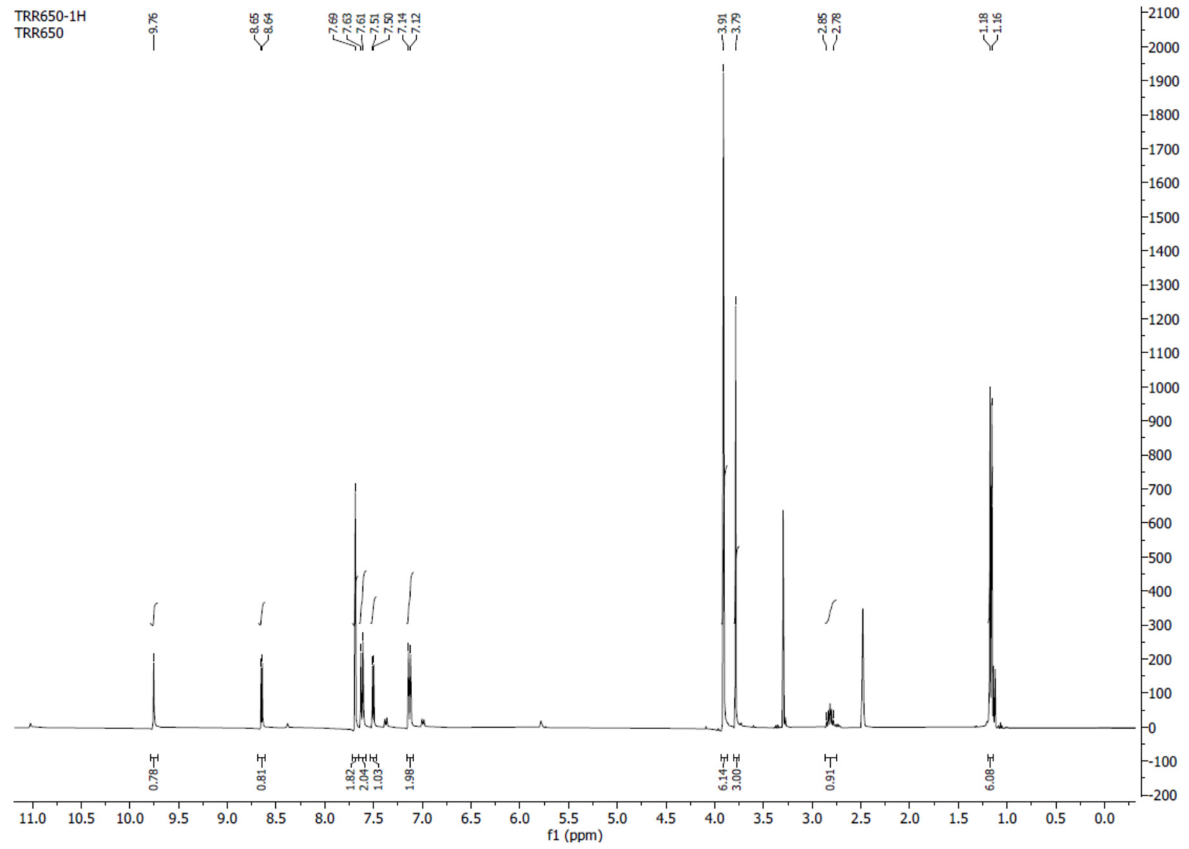

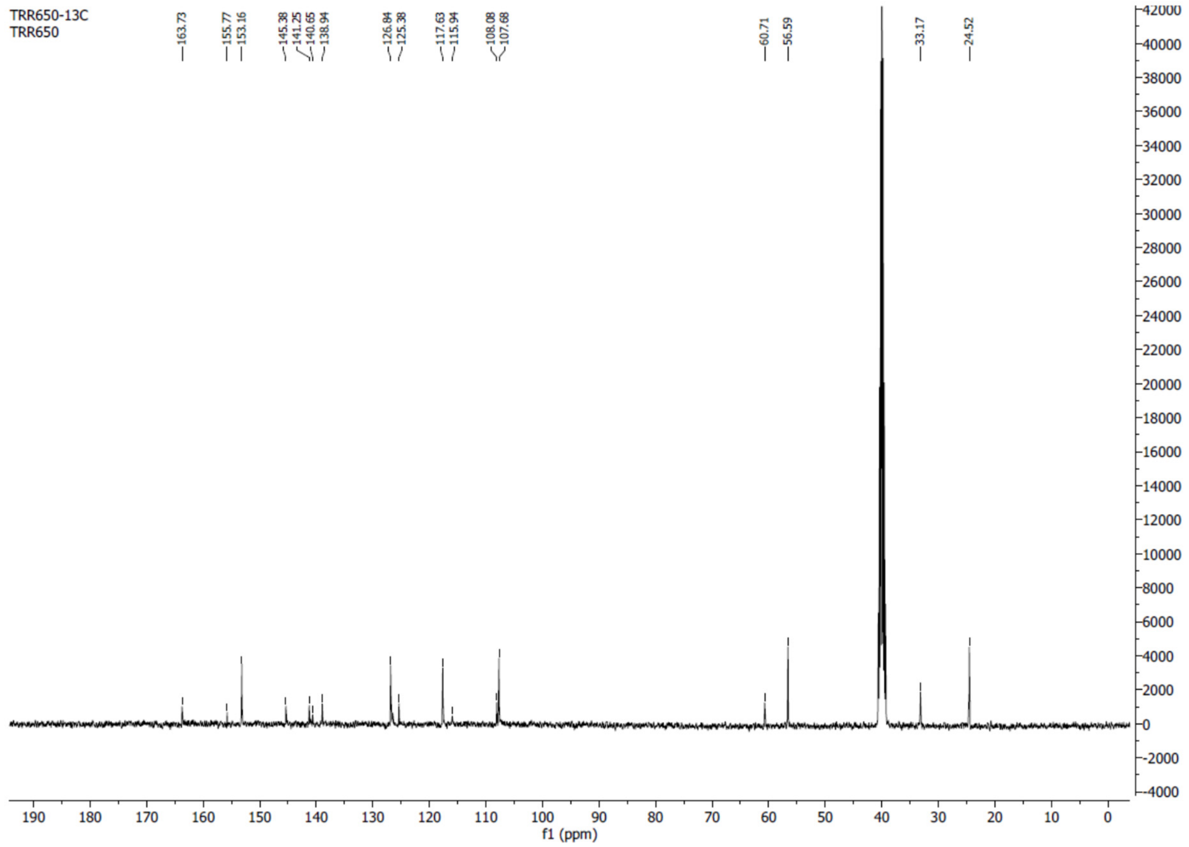

^{1}H-NMR and ^{13}C-NMR spectra of compound **3j**

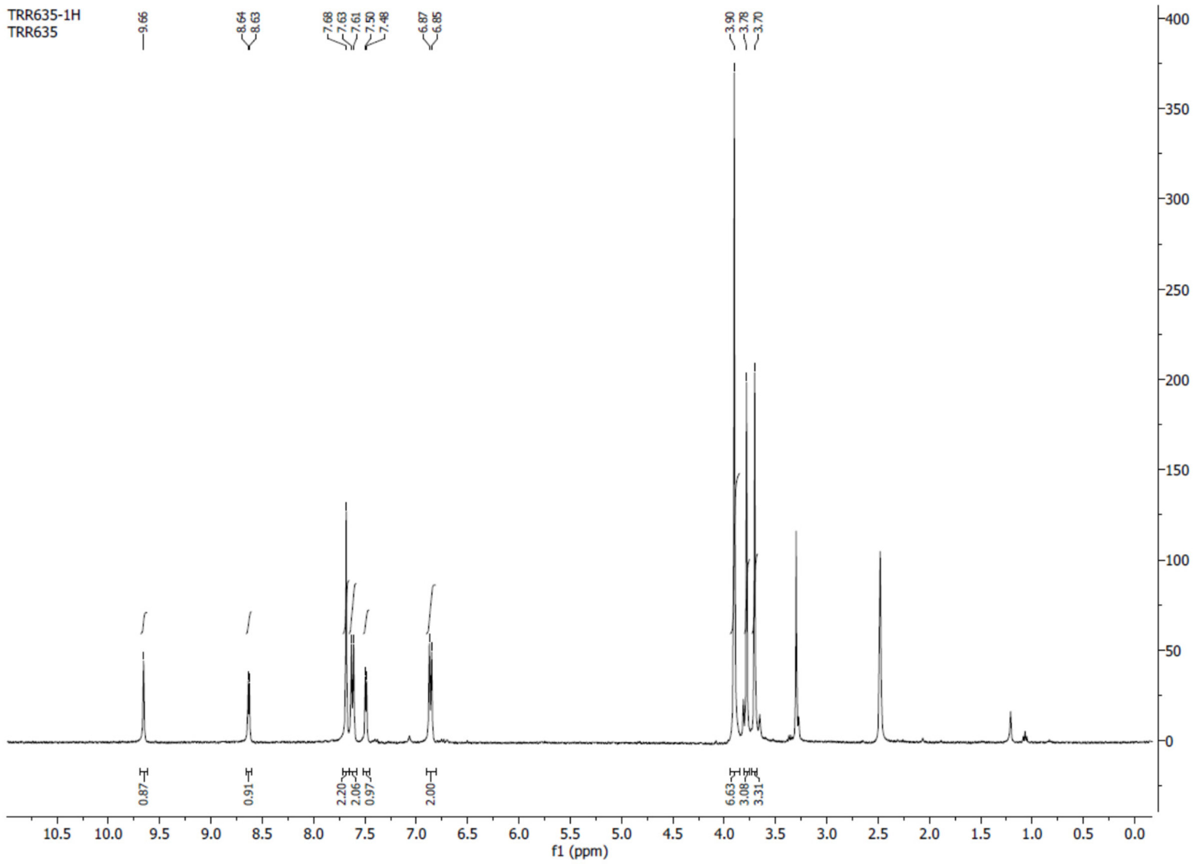

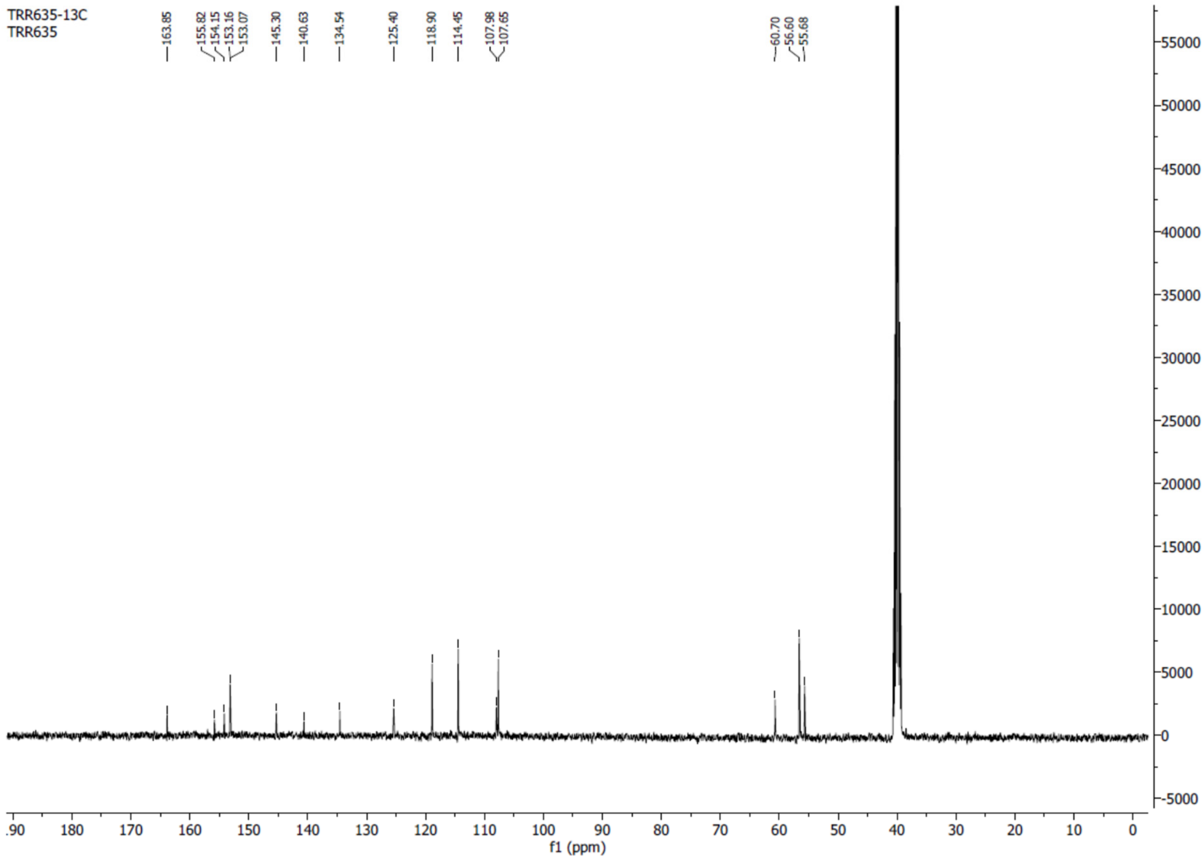

^{1}H-NMR and ^{13}C-NMR spectra of compound **3k**

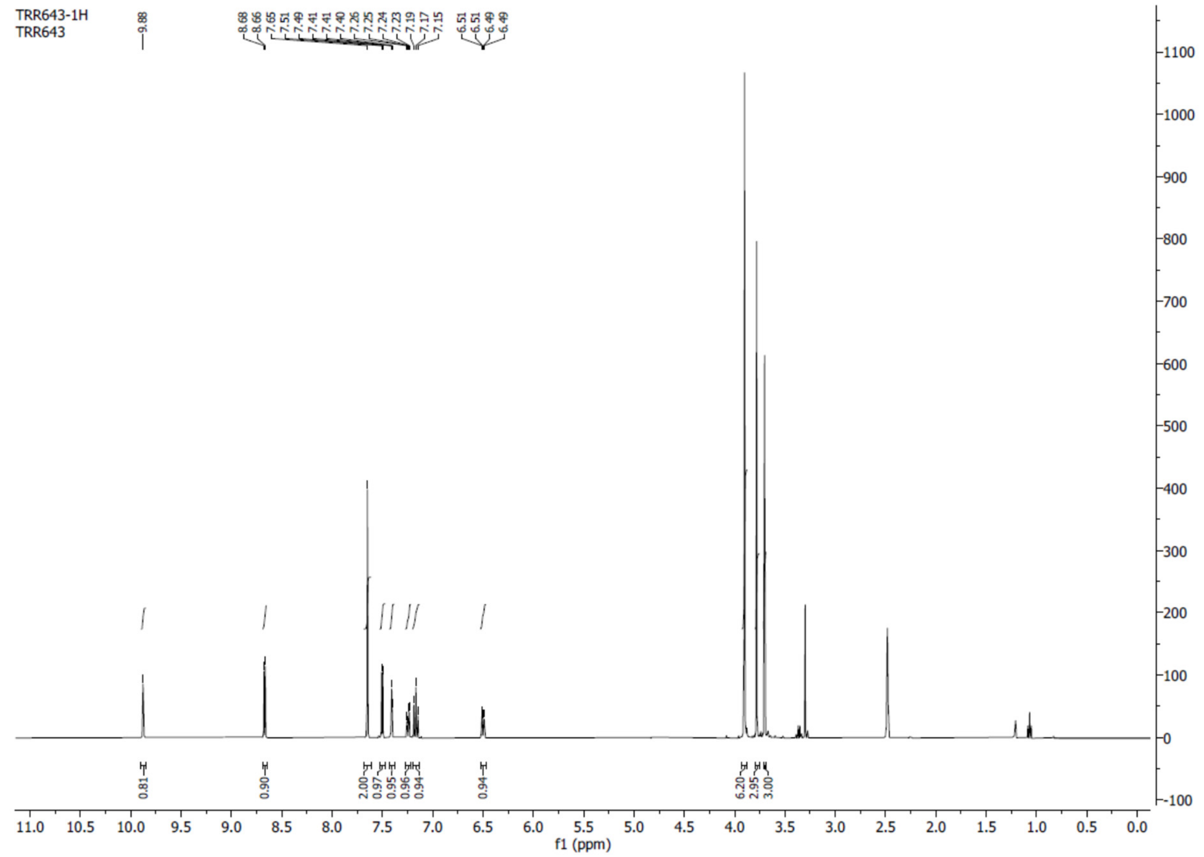

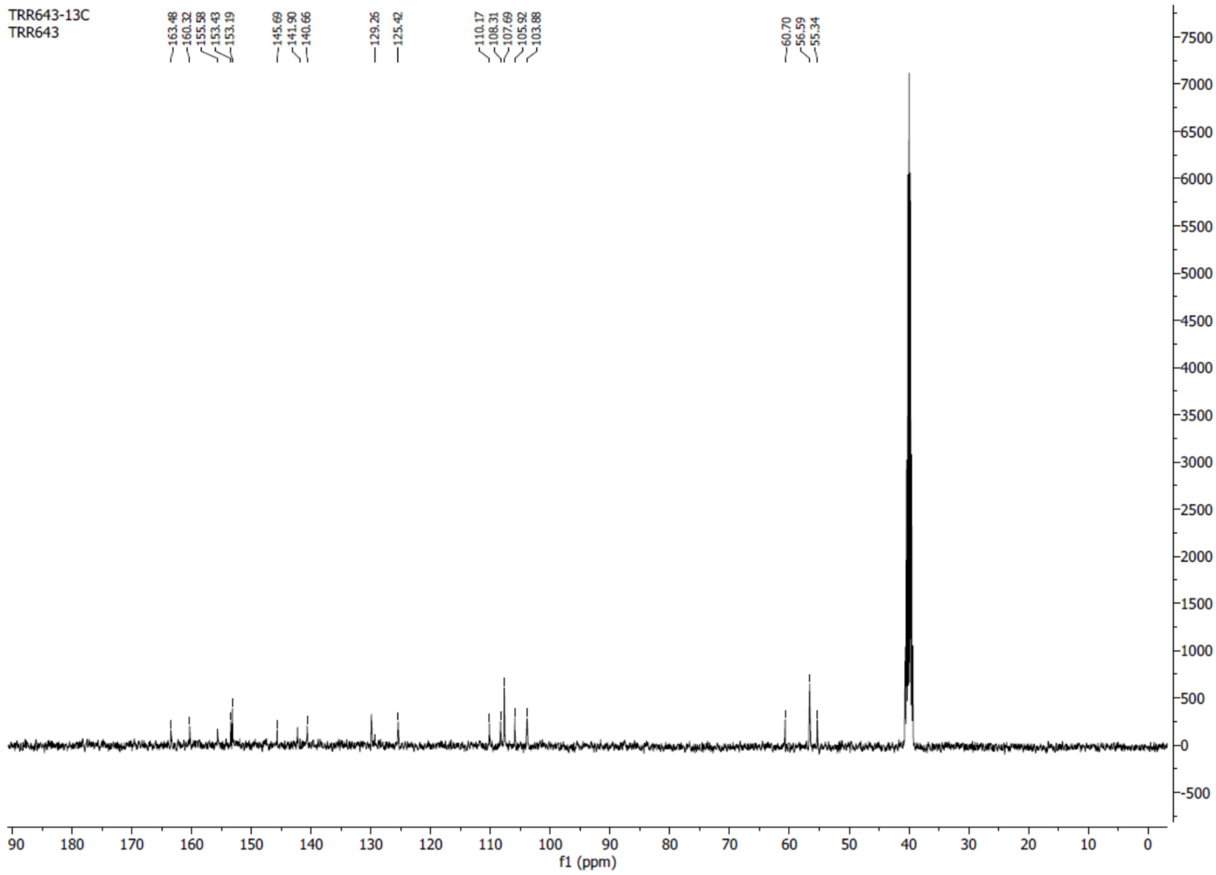

^{1}H-NMR and ^{13}C-NMR spectra of compound **3l**

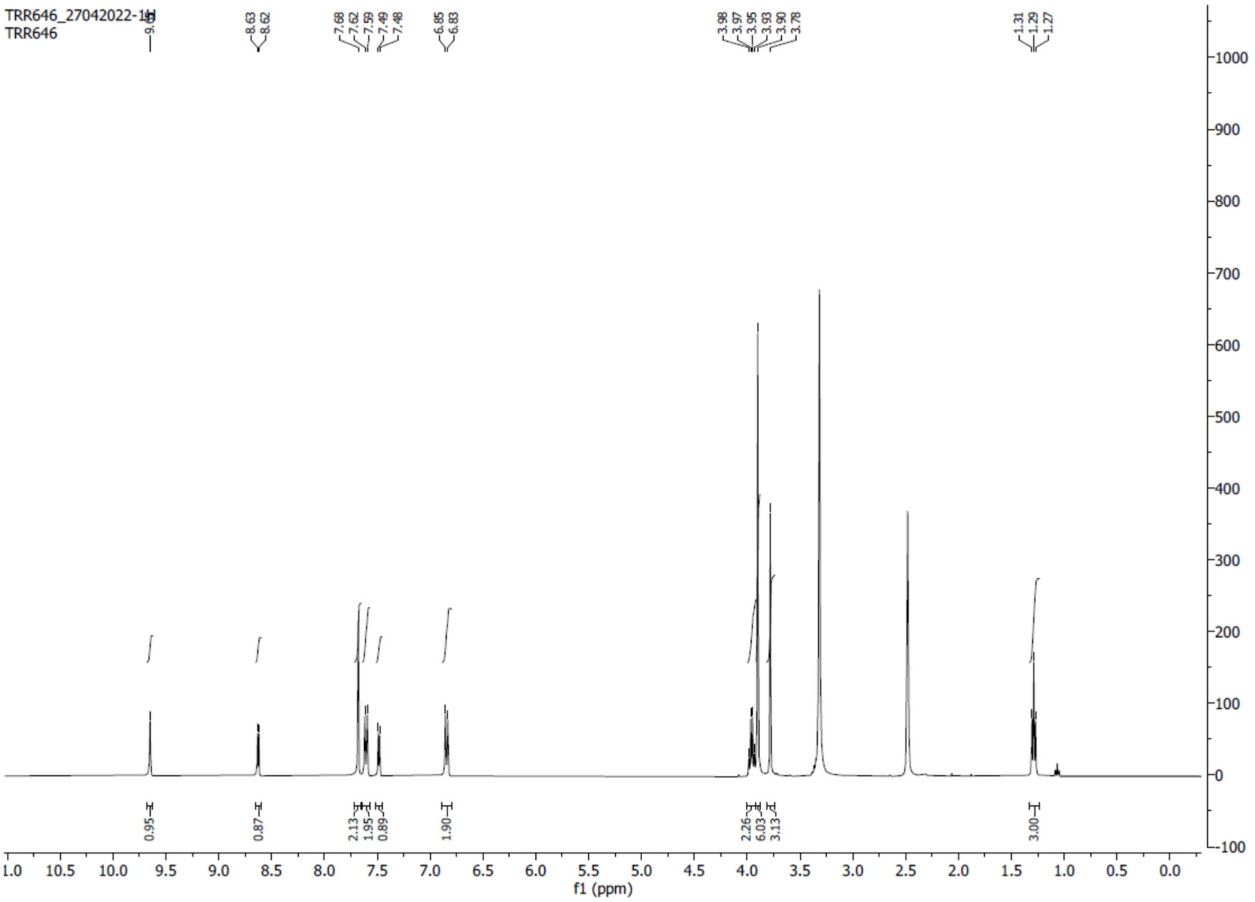

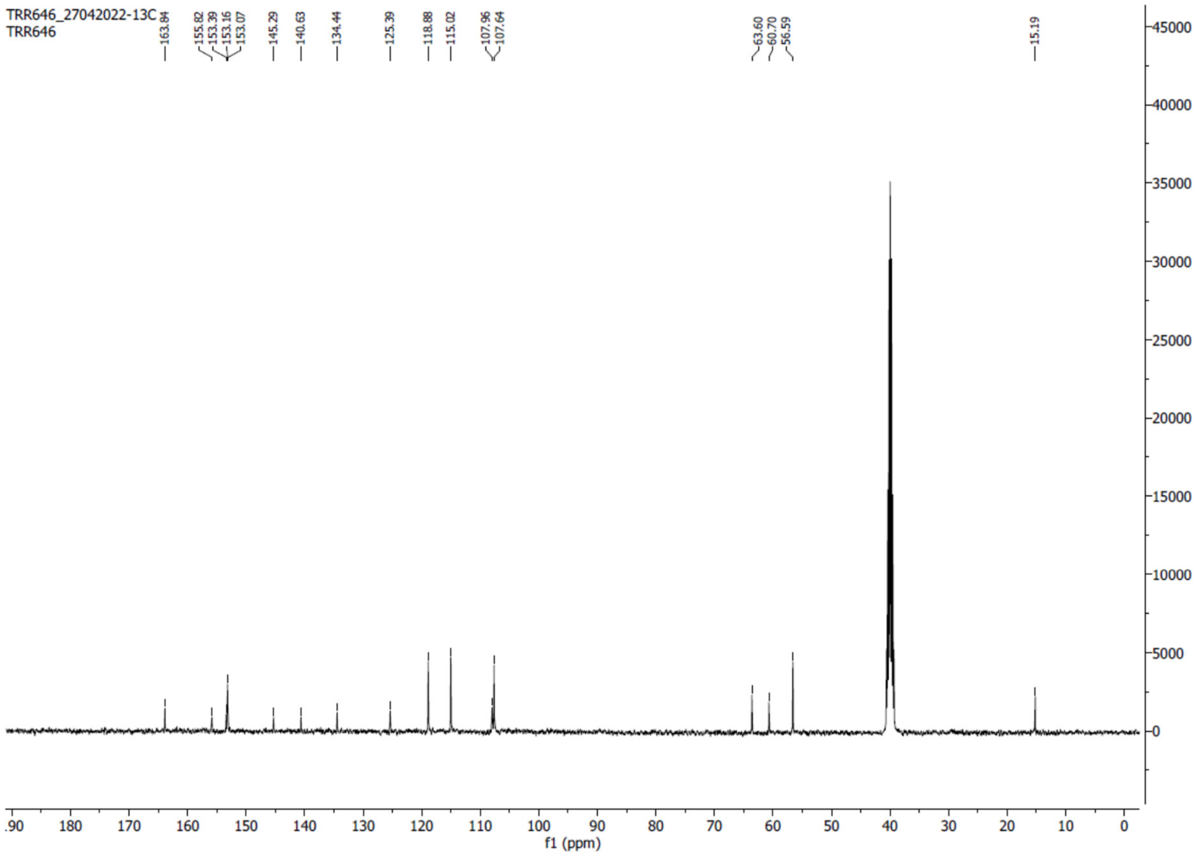

^{1}H-NMR and ^{13}C-NMR spectra of compound **3m**

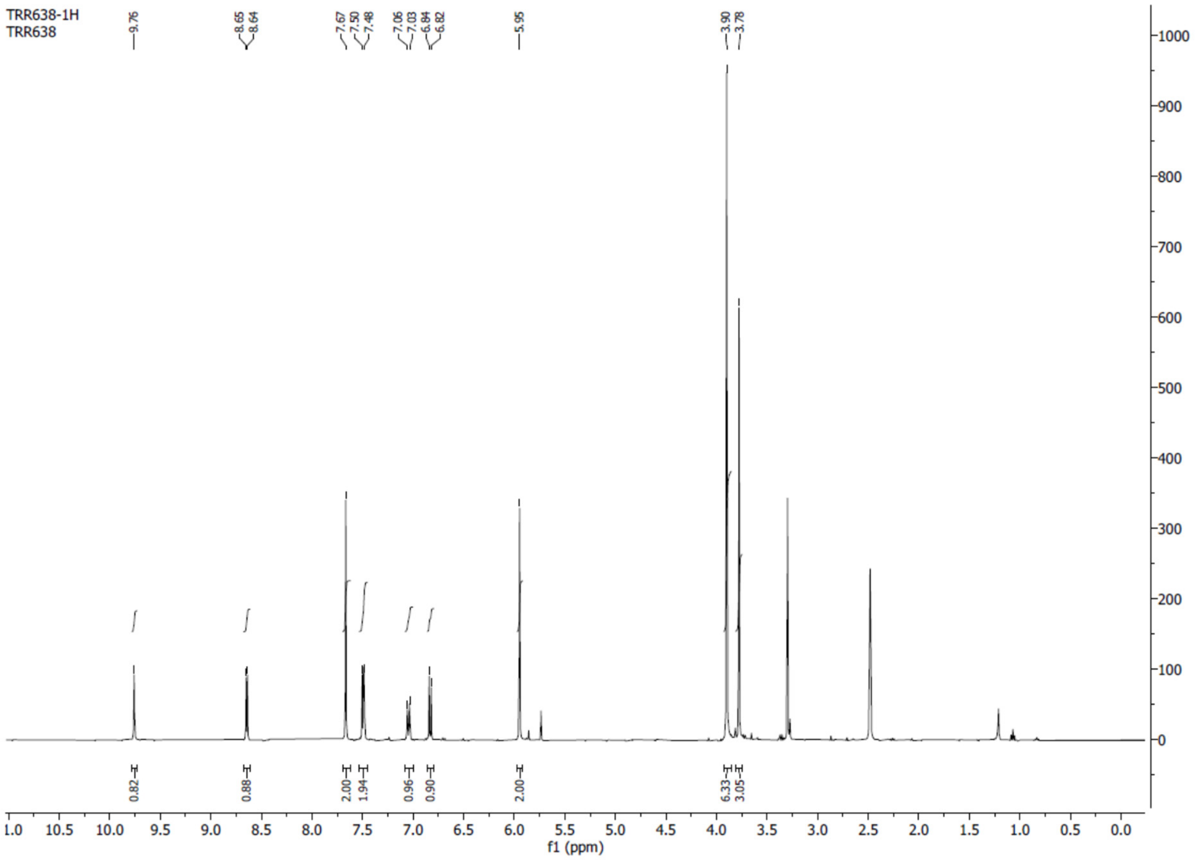

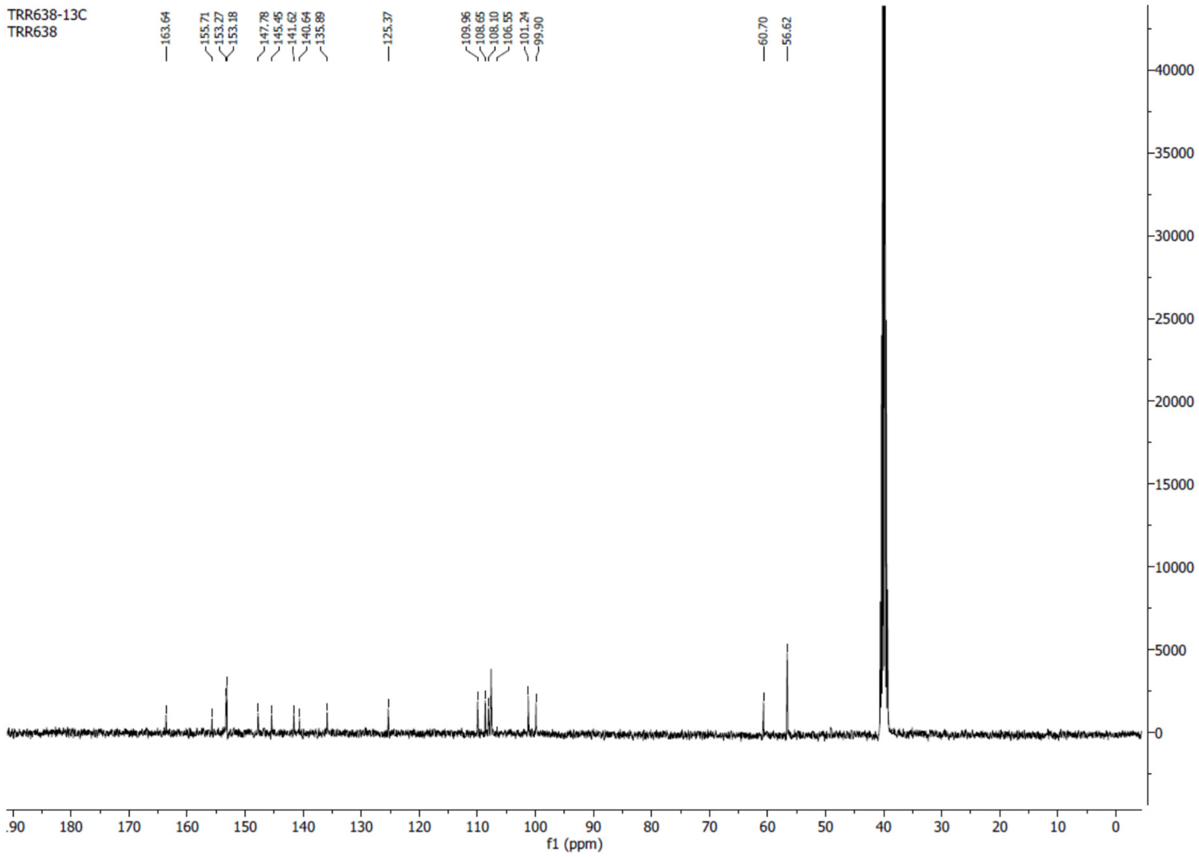

^{1}H-NMR and ^{13}C-NMR spectra of compound **3n**

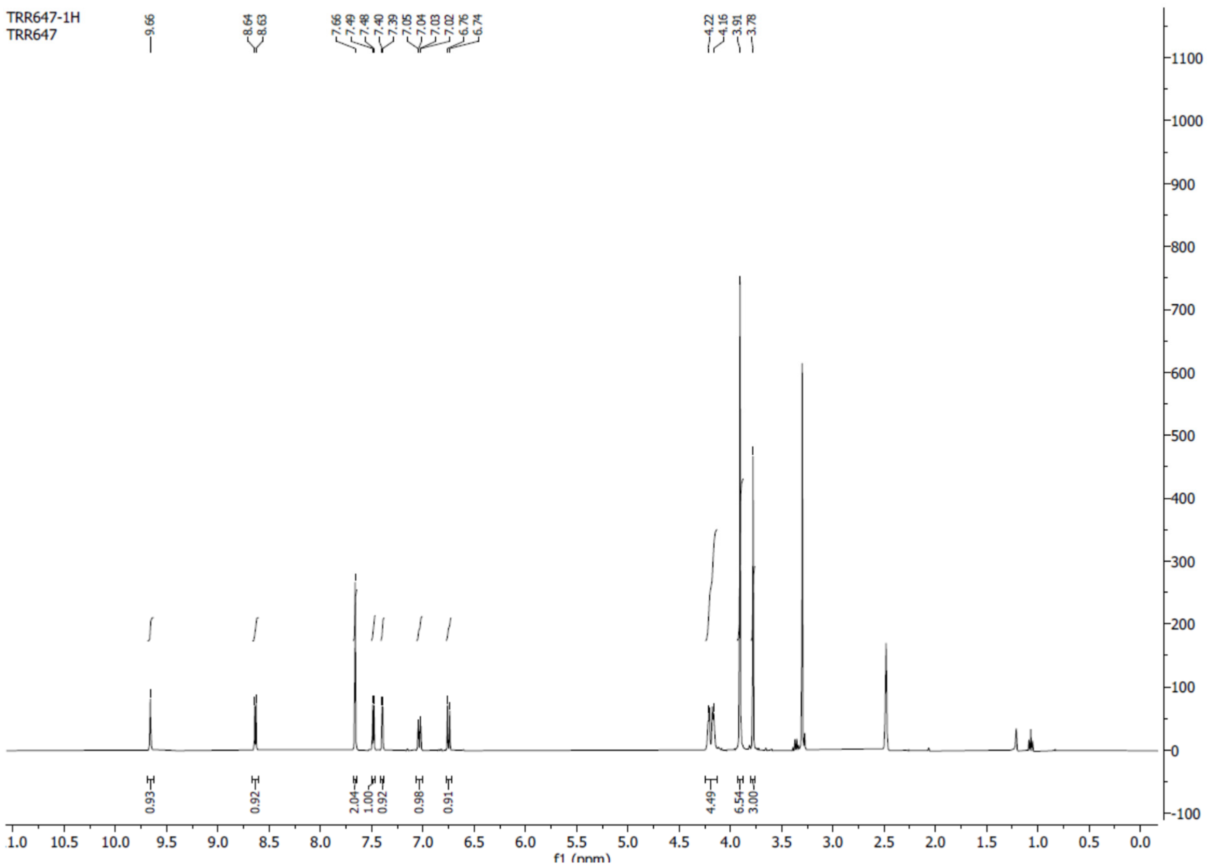

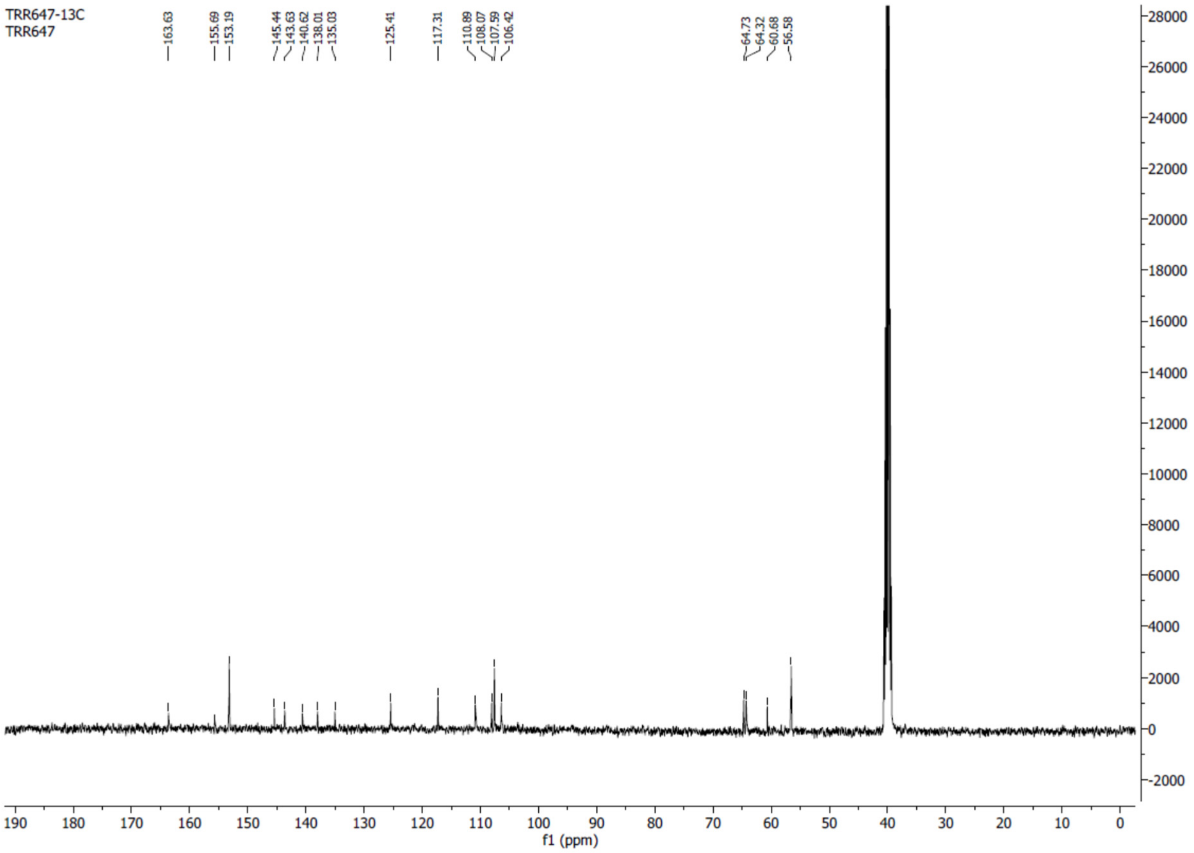

^{1}H-NMR and ^{13}C-NMR spectra of compound **3o**

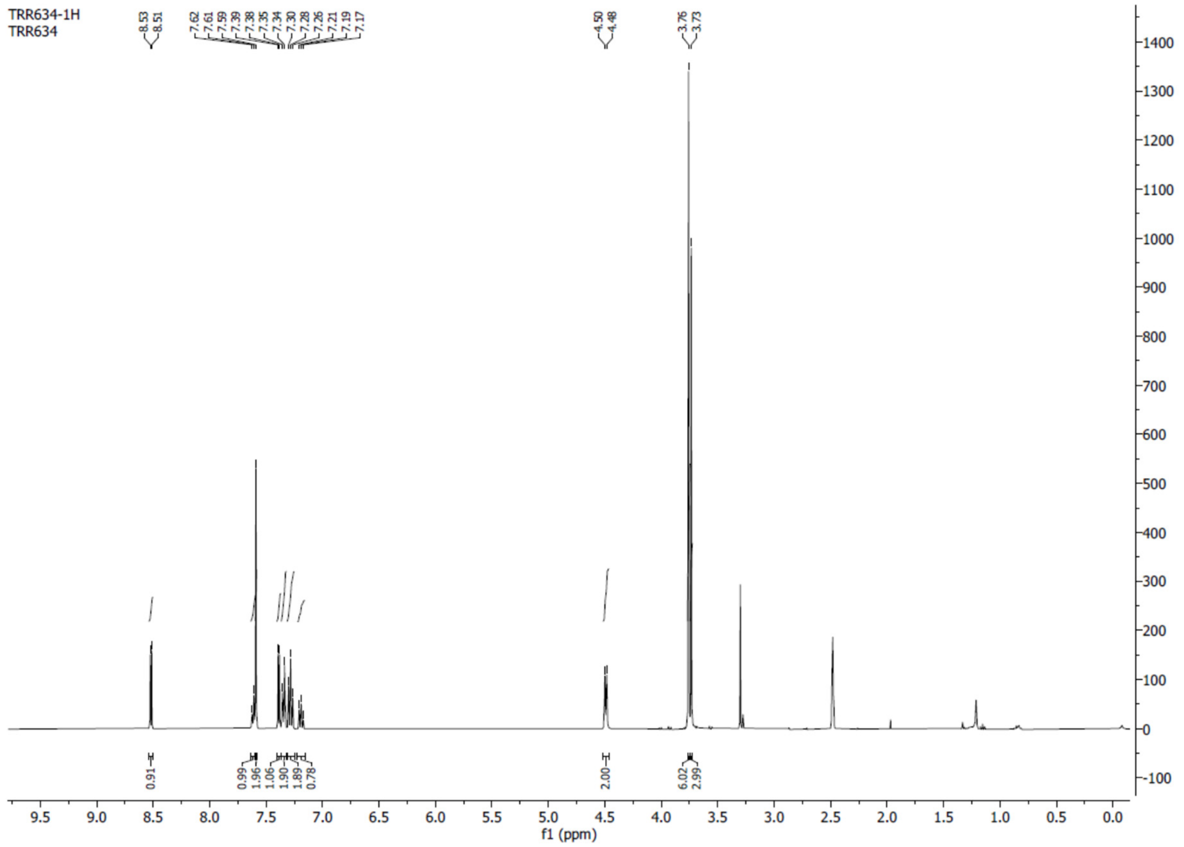

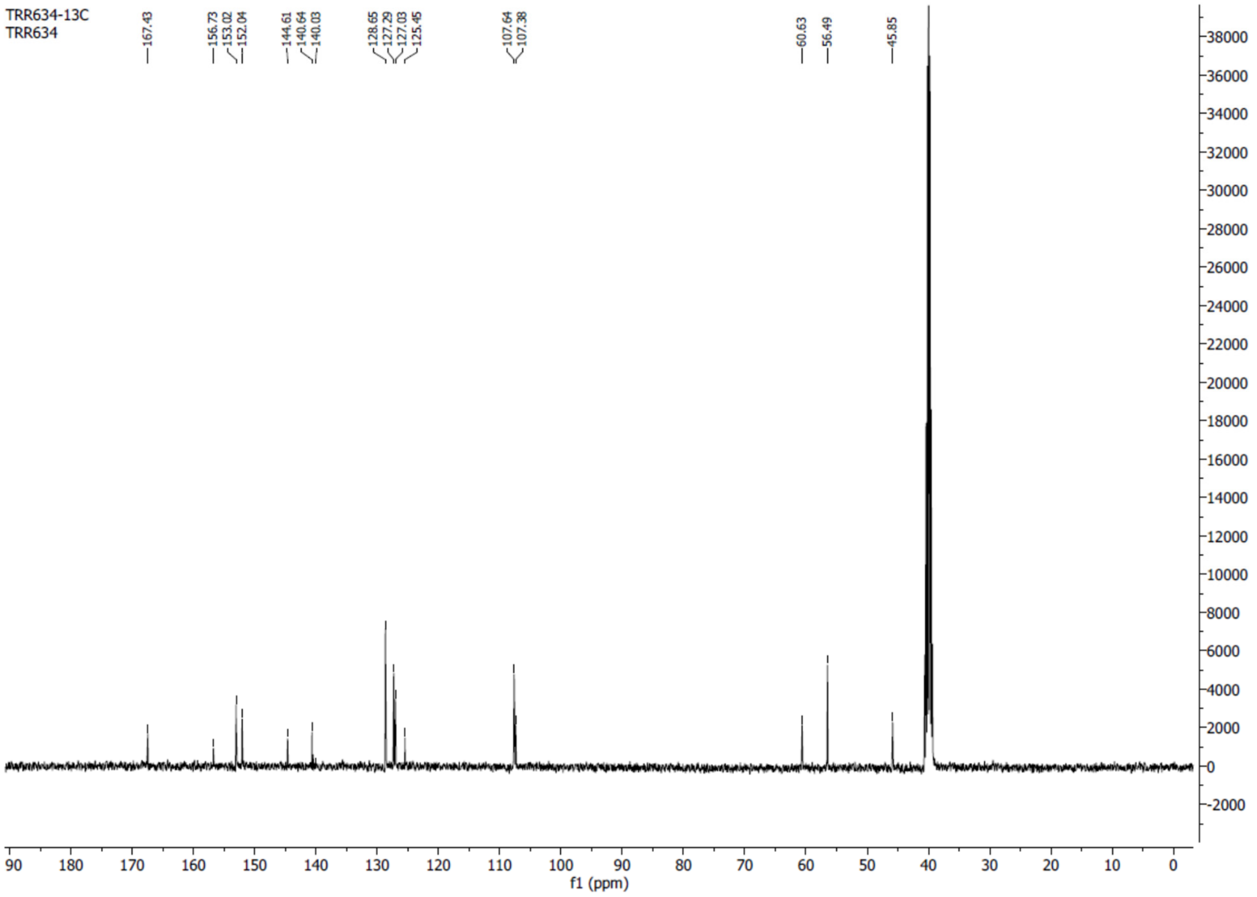

^{1}H-NMR and ^{13}C-NMR spectra of compound **3p**

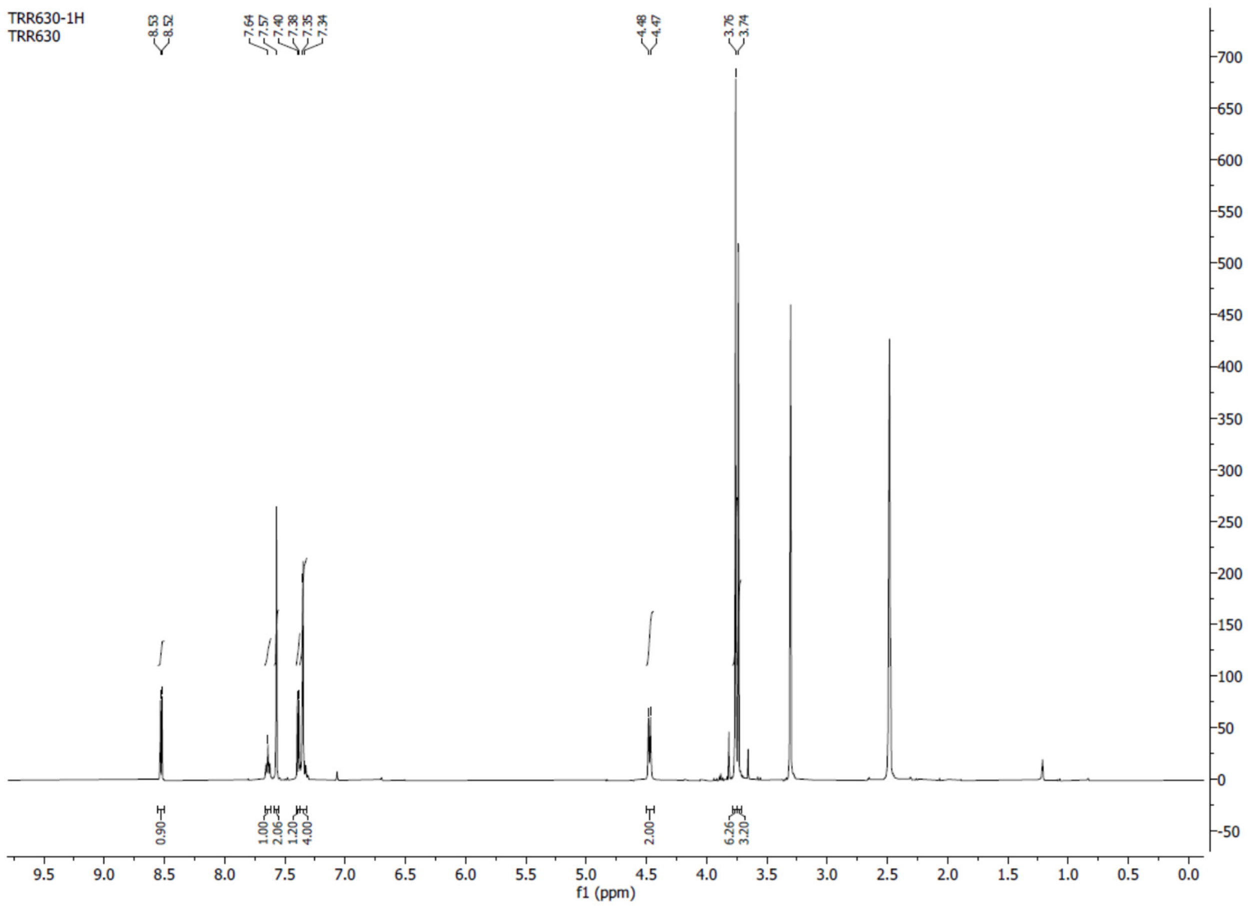

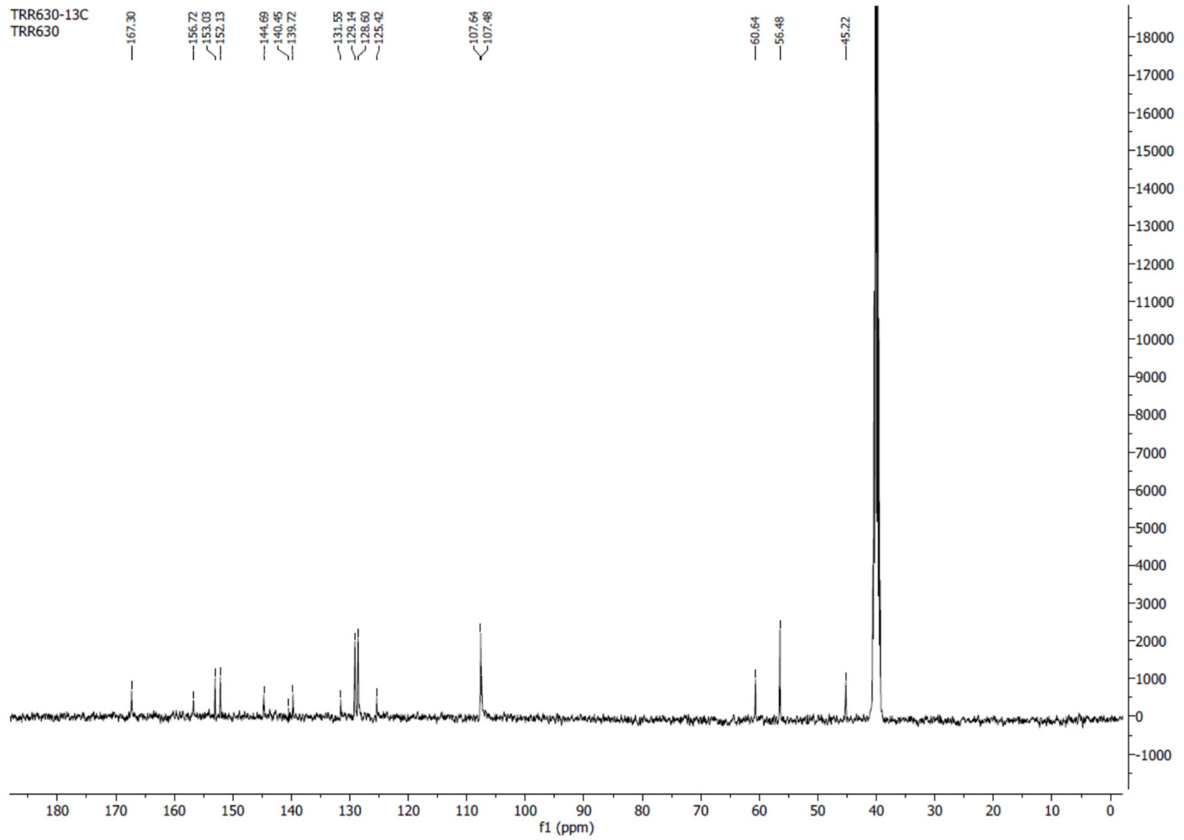

^{1}H-NMR and ^{13}C-NMR spectra of compound **3q**

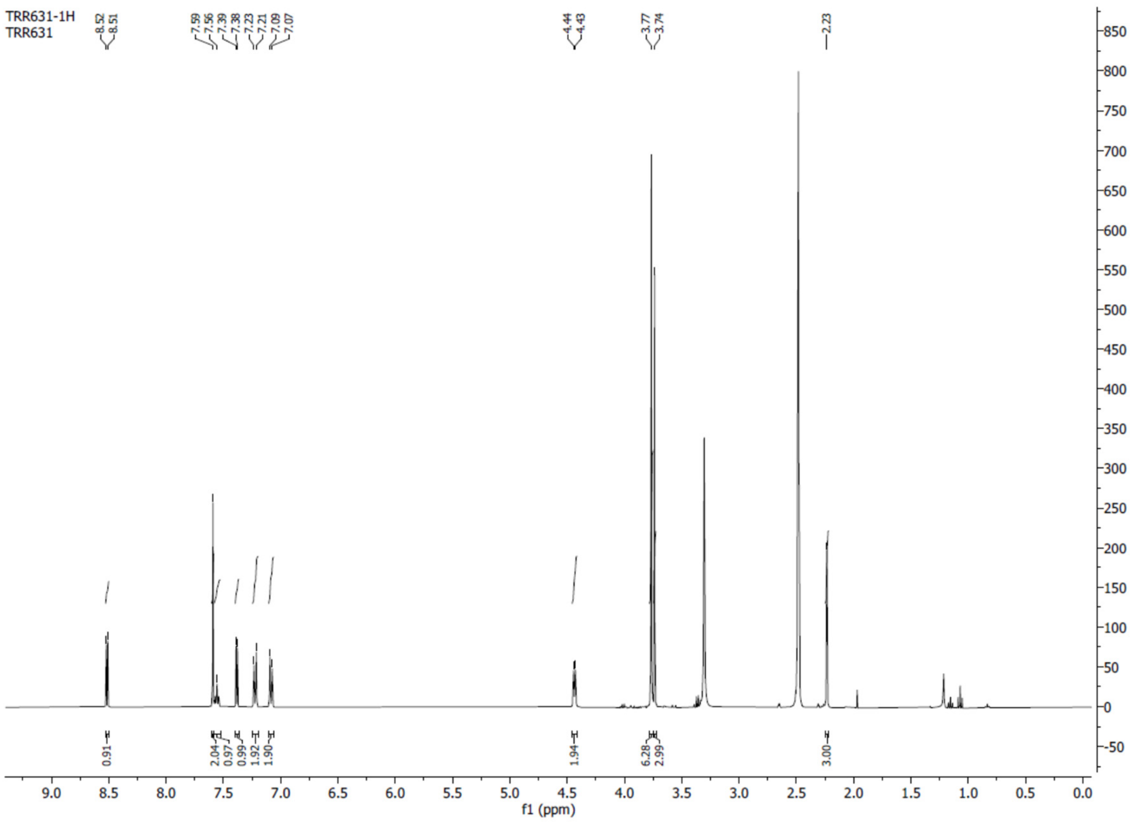

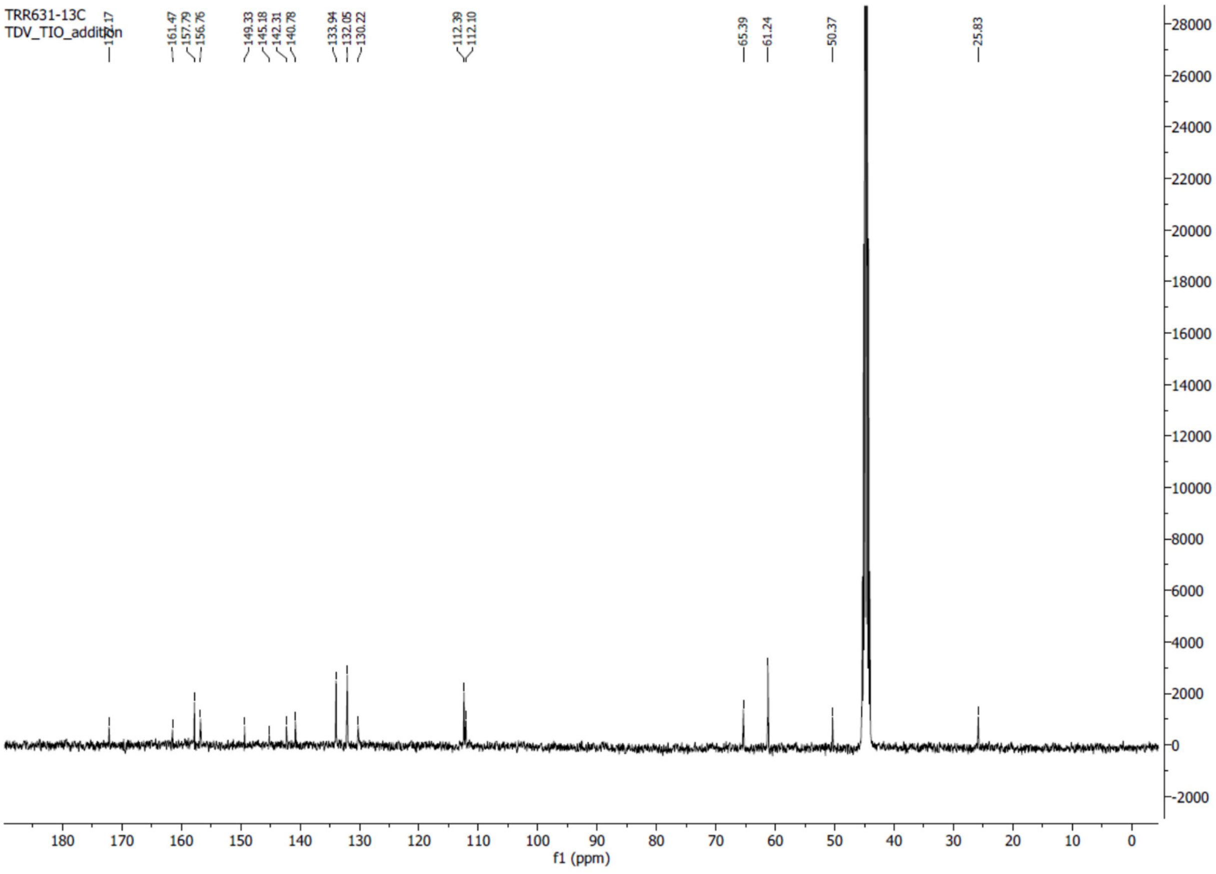

^{1}H-NMR and ^{13}C-NMR spectra of compound **3r**

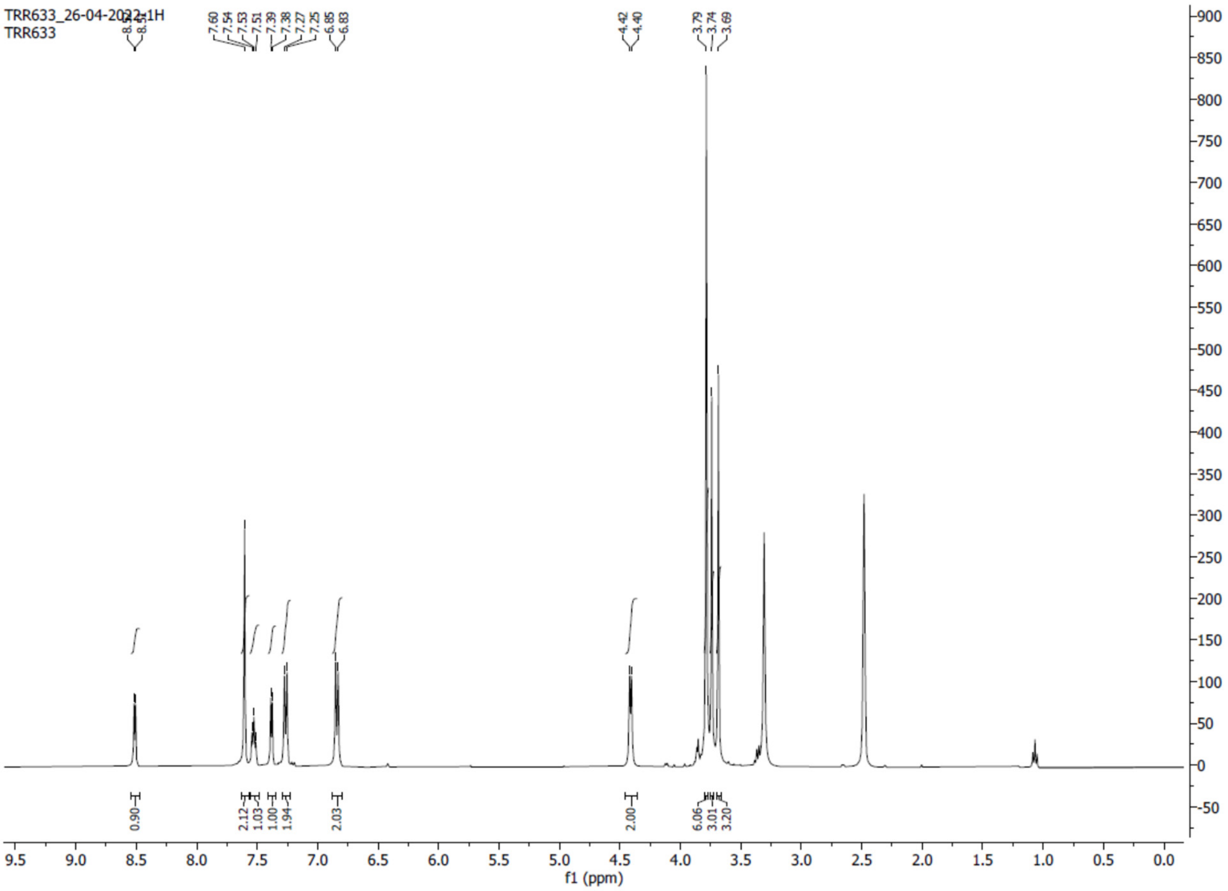

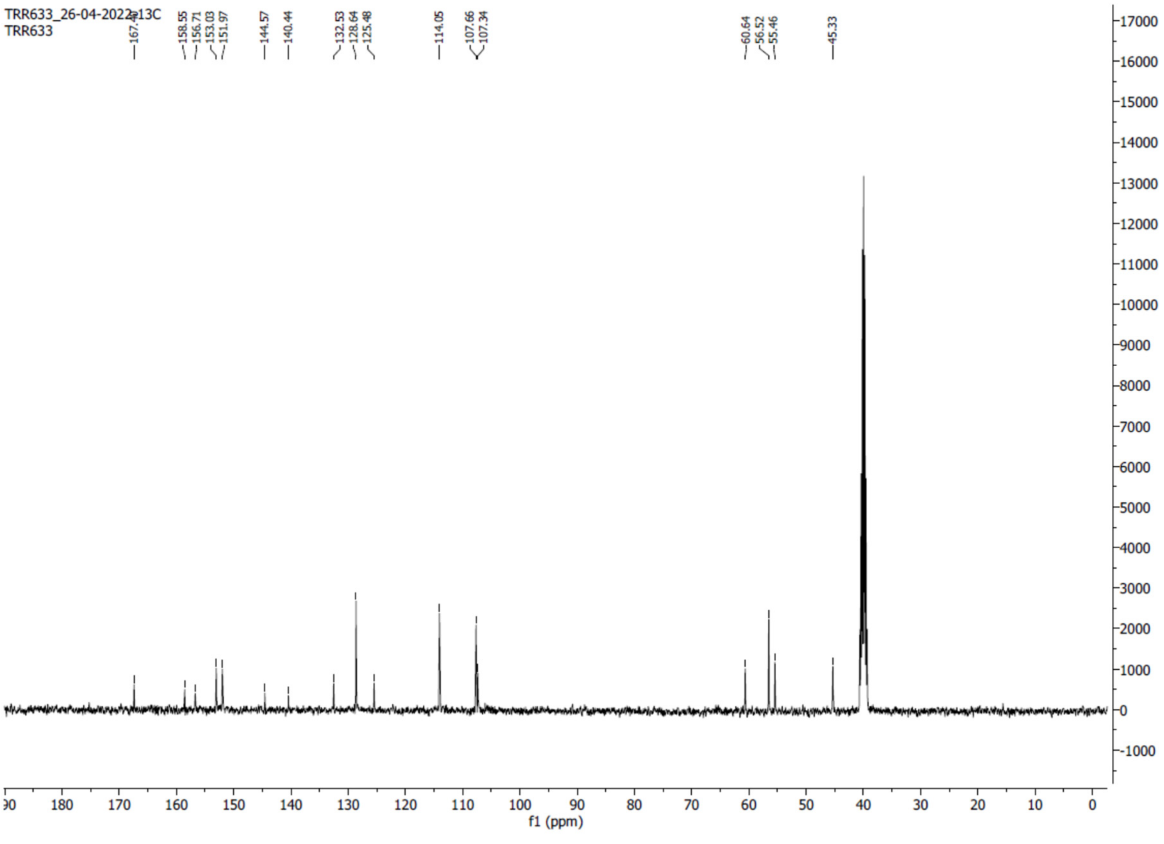

^{1}H-NMR and ^{13}C-NMR spectra of compound **3s**

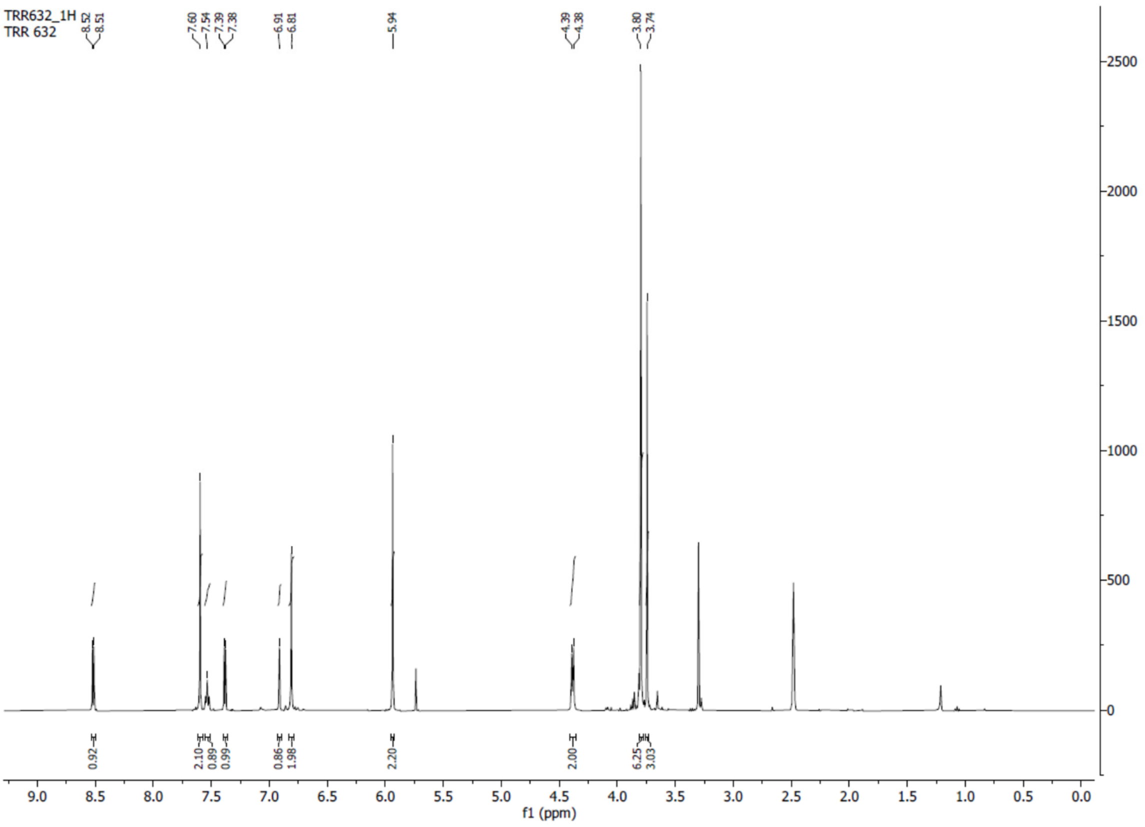

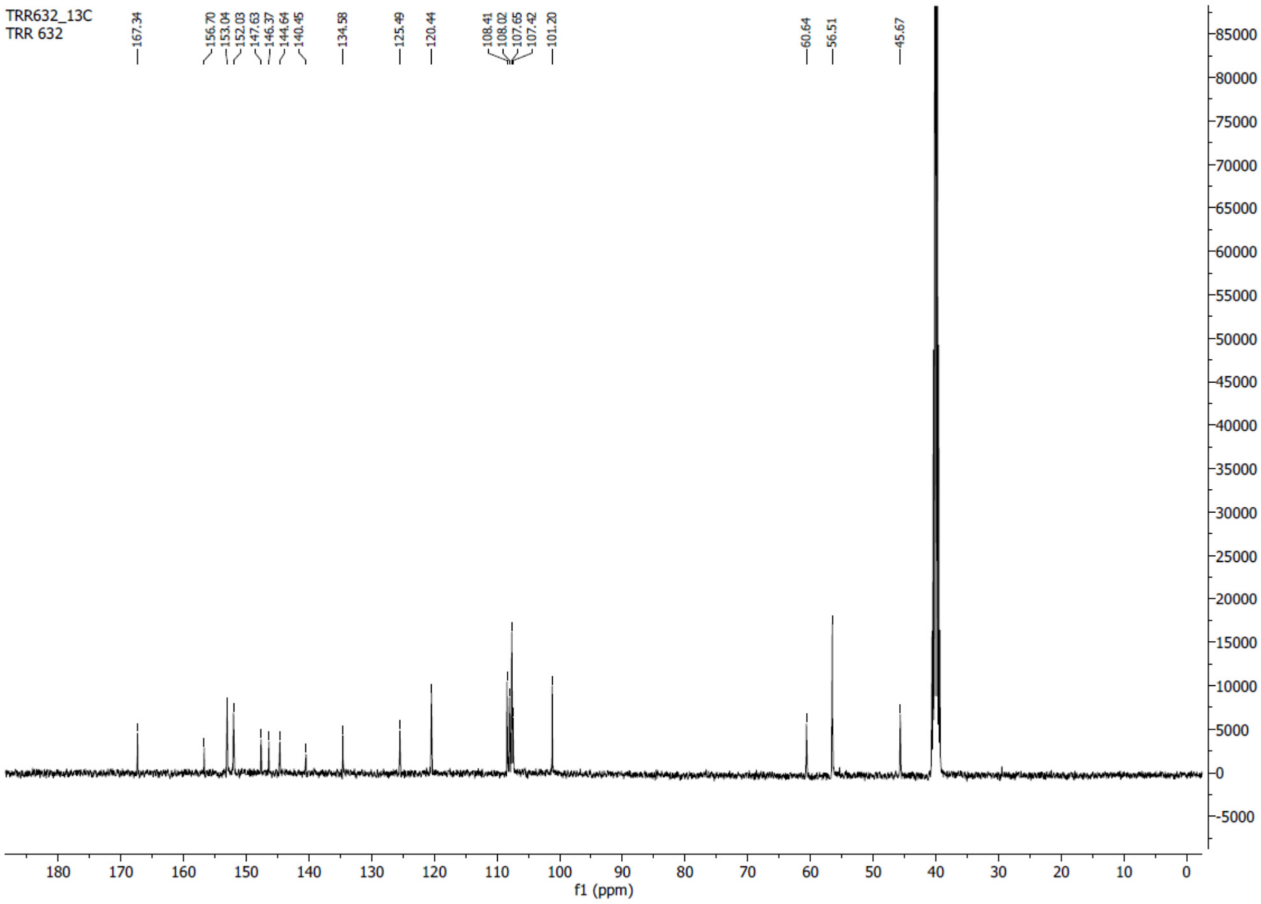

^{1}H-NMR and ^{13}C-NMR spectra of compound **3t**

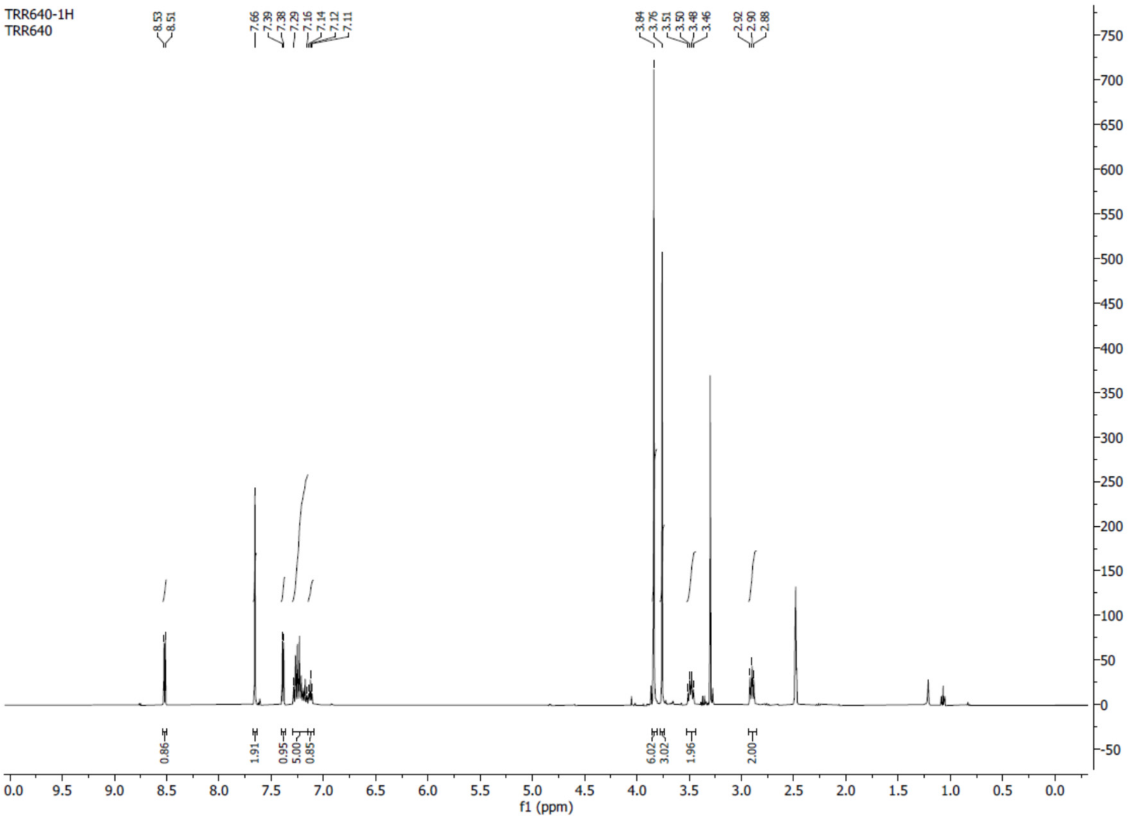

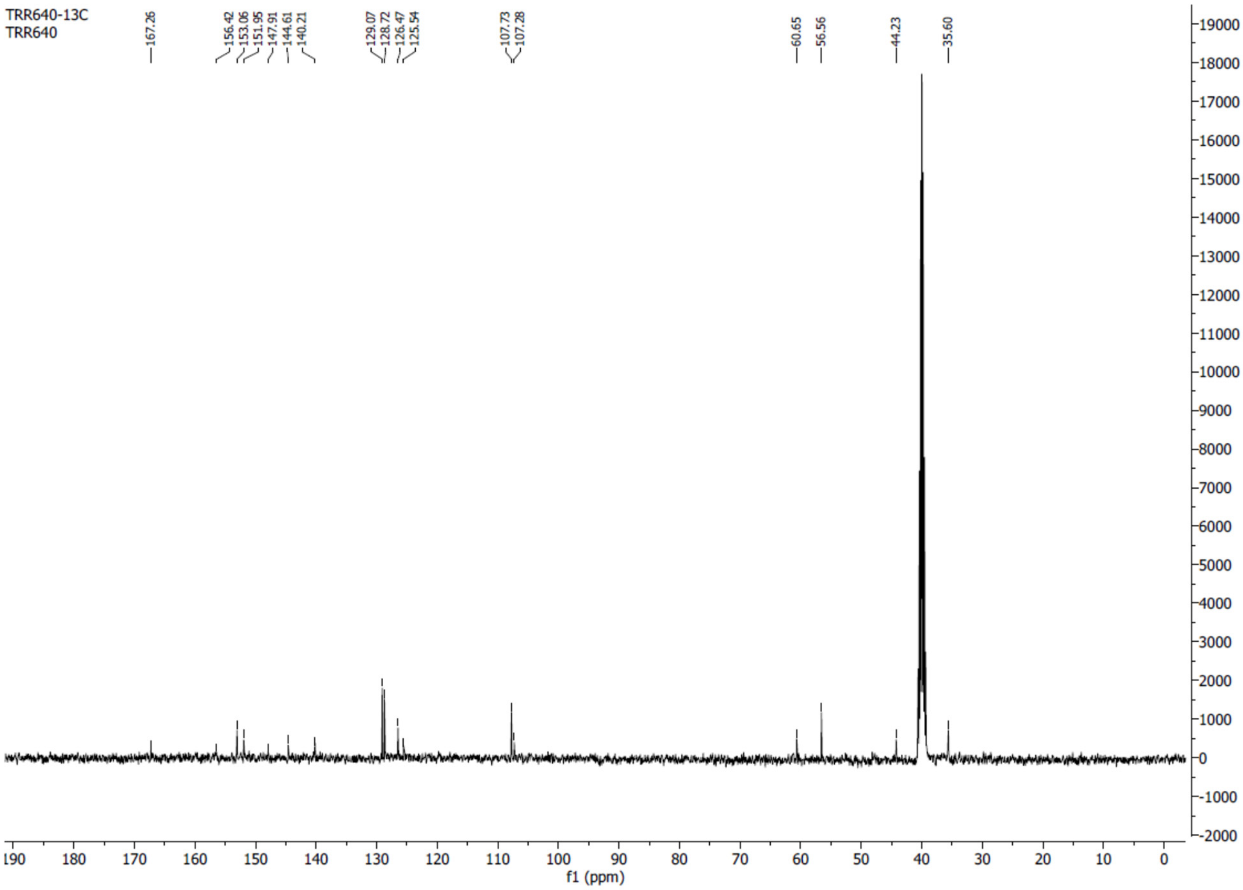

^{1}H-NMR and ^{13}C-NMR spectra of compound **3u**

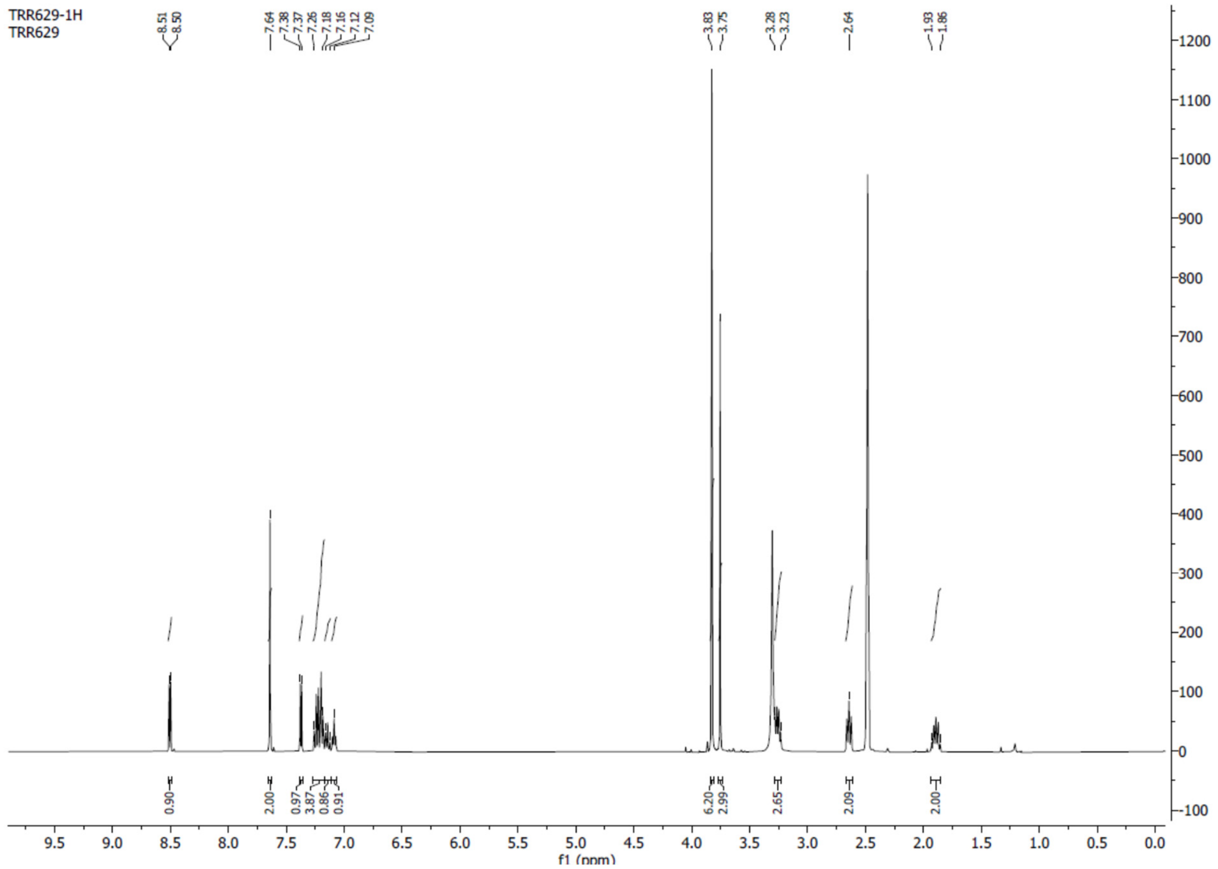

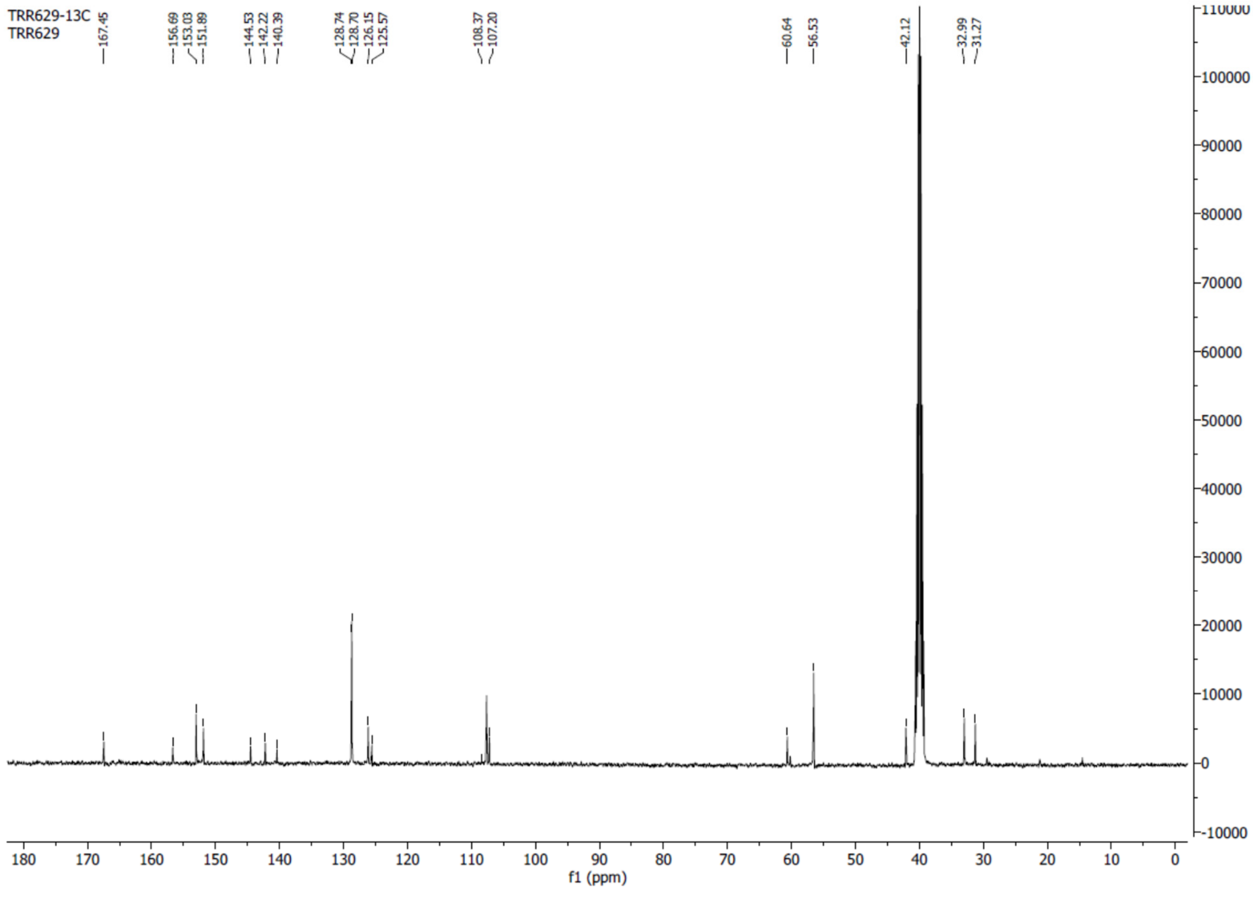

^{1}H-NMR and ^{13}C-NMR spectra of compound **3v**
